# Contrasting distributions and expression characteristics of transcribing repeats in *Setaria viridis*

**DOI:** 10.1002/tpg2.20551

**Published:** 2025-01-09

**Authors:** Ana Luiza Franco, Wenjia Gu, Petr Novák, Ilia J. Leitch, Lyderson F. Viccini, Andrew R. Leitch

**Affiliations:** ^1^ Institute of Biological Sciences, Federal University of Juiz de Fora Juiz de Fora Minas Gerais Brazil; ^2^ School of Biological and Behavioural Sciences Queen Mary University of London London E1 4NS UK; ^3^ Biology Centre Czech Academy of Sciences České Budějovice Czech Republic; ^4^ Royal Botanic Gardens, Kew Richmond UK

## Abstract

Repetitive DNA contributes significantly to plant genome size, adaptation, and evolution. However, little is understood about the transcription of repeats. This is addressed here in the plant green foxtail millet (*Setaria viridis*). First, we used RepeatExplorer2 to calculate the genome proportion (GP) of all repeat types and compared the GP of long terminal repeat (LTR) retroelements against annotated complete and incomplete LTR retroelements (Ty1/copia and Ty3/gypsy) identified by DANTE in a whole genome assembly. We show that DANTE‐identified LTR retroelements can comprise ∼0.75% of the inflorescence poly‐A transcriptome and ∼0.24% of the stem ribo‐depleted transcriptome. In the RNA libraries from inflorescence tissue, both LTR retroelements and DNA transposons identified by RepeatExplorer2 were highly abundant, where they may be taking advantage of the reduced epigenetic silencing in the germ line to amplify. Typically, there was a higher representation of DANTE‐identified LTR retroelements in the transcriptome than RepeatExplorer2‐identified LTR retroelements, potentially reflecting the transcription of elements that have insufficient genomic copy numbers to be detected by RepeatExplorer2. In contrast, for ribo‐depleted libraries of stem tissues, the reverse was observed, with a higher transcriptome representation of RepeatExplorer2‐identified LTR retroelements. For RepeatExplorer2‐identified repeats, we show that the GP of most Ty1/copia and Ty3/gypsy families were positively correlated with their transcript proportion. In addition, guanine‐ and cytosine‐rich repeats with high sequence similarity were also the most abundant in the transcriptome, and these likely represent young elements that are most capable of amplification due to their ability to evade epigenetic silencing.

## Core Ideas

- An alternative tool for analyzing transcriptome repeats using raw reads was performed.
- Different patterns of repeat transcription from different tissues and transcriptome libraries were observed.
- Ribo‐depleted libraries show more expressed repeats than poly‐A libraries.
- Contrasting genomic features of repeats may influence their transcriptome proportion.
- Repeat expression varies according to the tissue type, with germ line tissues having particularly abundant repeat transcripts.

**Abbreviations**
GC: guanine and cytosine
GP: genome proportion
rDNA: ribosomal DNA
TE: transposable element
TP: transcriptome proportion

## 1 INTRODUCTION

Poaceae are one of the five largest flowering plant families in terms of number of species (c. 11,400 species). The family is of great economic importance (Hodkinson, 2018; Kellogg, 2001, 2017) and is fundamental to world food security (Devos, 2010; Gruber, 2017). To enhance crop productivity and resilience, there has been a long‐standing interest in characterizing and utilizing crop wild relatives (Vincent et al., 2019). One such example is *Setaria viridis* (green foxtail millet) in family Poaceae, a partially domesticated relative of the widely cultivated foxtail millet (*Setaria italica*) with significant potential as a crop in its own right (Pant et al., 2016). Here we focus on the transcription of repetitive elements in *S. viridis*. The species is chromosomally diploid (2*n* = 2*x* = 18) with a relatively small genome size (0.78 Gb/1*C*; Bennett et al., 1998) for a species in Poaceae (Poaceae mean 1*C*‐value = 5 Gb/1*C*, Plant DNA *C*‐values database [Pellicer & Leitch, 2020]).

One feature of many plant genomes, including in species of Poaceae, is that they contain an abundance of repetitive sequences, especially transposable elements (TEs). For example, TEs comprise c. 80%–86% of the maize genome (Lin et al., 2021), c. 55% of sorghum (*Sorghum bicolor*) (Kuo et al., 2021), c. 40% of rice (Du et al., 2017), c. 40% of *S. italica* (Bennetzen et al., 2012; Yadav et al., 2015), and c. 28% of the *Brachypodium distachyon* genome (The International *Brachypodium* Initiative, 2010; Stritt et al., 2019). Indeed, the proportion of the genome comprising repeats scales more or less linearly with genome size in plant genomes up to around 10 Gb/1*C*, above which the genome proportion (GP) that is occupied by repeats typically declines with further increases in genome size (Novák, Guignard, et al., 2020).

TEs can broadly be classified into those that utilize an RNA intermediate for mobility and amplification (class I RNA retroelements) and those that do not (class II DNA transposons) (Neumann et al., 2019). Of these, the most abundant elements in angiosperms, including in Poaceae, typically belong to the long terminal repeat (LTR) retroelement superfamilies Ty1/copia and Ty3/gypsy. Many of these repeats are actively transcribed, and their transcripts have been identified among expressed sequence tags (e.g., Meyers et al., 2001) and in short‐read Illumina transcriptome libraries (reviewed in Lanciano & Cristofari, 2020). However, little is known about the overall transcription of all categories of repeats and the distinction between repeats that are actively transcribing for their own “selfish” amplification (identified in poly‐A transcriptome libraries) and those being targeted for silencing (identified in short‐read and ribo‐depleted transcriptome libraries). For example, while Anderson et al. (2019) reported that 15% of all TE lineages in the maize genome had transcripts in poly‐A libraries, such values will miss any transcribed repeats without poly‐A tails, as in those targeted for silencing by the RNA‐directed DNA methylation (RdDM) pathway (Kenchanmane Raju et al., 2019; Matzke et al., 2015).

While advances in the development of high‐throughput sequencing technologies are enabling significant progress in our understanding of TE regulation in plants (Anderson et al., 2019; Jiang & Ramachandran, 2013; Kirov et al., 2020), characterizing the relative abundance of different transcribed repeats remains computationally challenging, mainly due to their diversity (Lanciano & Cristofari, 2020). In order to identify transcribed repeats, different programs and strategies have been developed (Criscione et al., 2014; Jeong et al., 2018; Jin et al., 2015; Kong et al., 2019; Lerat et al., 2017; Yang et al., 2018), typically involving mapping of transcriptome sequence reads against reference genomes, assembled transcriptomes, or TE consensus sequences (Bao et al., 2015; Lerat et al., 2017). However, even with a well‐assembled genome, it remains challenging to accurately assemble long tracks of repeats, and it is difficult to identify novel repeats that can vary from a few bases to megabases in length.

A motivation of this study was to determine if repeat transcription could be studied using repeats identified from genome skimming data and hence applicable to any organism, irrespective of whether a genome assembly is available or not. RepeatExplorer2 is a method that builds a library of all types of genomic repeats (e.g., LTR retroelements, DNA transposons, and satellites) from genome skimming data and was used here to analyze Illumina short read sequences available for *S. viridis* from Mamidi et al. (2020). The method identifies consensus sequences of repeats and cannot distinguish between complete and incomplete repeats in the genome. Default settings of RepeatExplorer2 define “repeats” as sequences with ≥90% sequence similarity spanning at least 55% of Illumina read lengths (Novák, Neumann, & Macas, 2020). This is important because newly amplified repeats are at first identical, or nearly so, but then diverge through a combination of point mutations, indels, rearrangements, and gene conversion events (Lanciano & Cristofari, 2020). Indeed, over long evolutionary timeframes, repeats can mutate so much that they are no longer distinguishable as repeats and instead appear as low‐copy or unique sequences that have been described as “dark matter” (Maumus & Quesneville, 2014). In this continuum between repeat sequences and “dark matter,” RepeatExplorer2 assembles contigs that summarize the repeat content of the *S. viridis* genome, identifying all repeats that occur in c. 200 copies or more. To these repeat contigs and, for comparison to DANTE‐annotated (i.e., domain‐based annotation of TEs) LTR retroelements from assembled genomes, we mapped RNA‐seq reads to characterize the overall repeat transcript proportions (TPs) of the different classes of repeats present in the genome.

Previously, Yadav et al. (2015) studied transcriptionally active retroelements in tissues of *S. italica* and found that Ty1/copia elements were the most highly expressed. Here we extend that analysis to its close relative *S. viridis* to gain an overall picture of repetitive element transcription in two different types of transcriptome libraries (poly‐A and ribo‐depleted libraries) from four tissues, available in transcription databases (Sebastian et al., 2016; Yang et al., 2018) and newly sequenced transcriptomes here.

We analyzed repeats in (i) poly‐A RNA libraries, which comprise transcripts produced by RNA polymerase II and include polyadenylated messenger RNA transcripts from protein‐coding genes, and including those coming from repeat genes that are needed for the repeat's own selfish amplification (e.g., the retrotransposon genes that code for the polyprotein needed for the amplification of LTR retrotransposons). (ii) Ribosomal RNA‐depleted (ribo‐depleted) RNA libraries that should include transcripts of any repeat that is being transcribed, including not only those from (i) but also DNA transposons and any retroelement or other repeat that is being transcribed as part of the repeat silencing pathways via RdDM. Transcripts from the RdDM pathway are produced by the transcriptional activity of RNA polymerase IV and V templating off the repeats themselves (Kenchanmane Raju et al., 2019; Matzke et al., 2015).

**Core Ideas**

- An alternative tool for analyzing transcriptome repeats using raw reads was performed.
- Different patterns of repeat transcription from different tissues and transcriptome libraries were observed.
- Ribo‐depleted libraries show more expressed repeats than poly‐A libraries.
- Contrasting genomic features of repeats may influence their transcriptome proportion.
- Repeat expression varies according to the tissue type, with germ line tissues having particularly abundant repeat transcripts.

Using the estimated proportion of repeat transcripts in the transcriptomes (i.e., TP) of four different tissues, we tested the following hypotheses:

1. The TP of individual repeat types will reflect their abundance in the genome (i.e., their GP), especially in ribo‐depleted libraries, assuming all repeats in the genome are equally likely to be suppressed by RdDM.
2. The highest repeat TP will be from genomic repeats with the highest guanine and cytosine (GC) content and sequence similarities. This is because repeat copies that are GC‐rich and share high levels of sequence similarity are most likely to be those that are young and active. In contrast, more ancient repeats are likely to include mutations (indels, point mutations) and hence have reduced sequence similarity, including mutations arising from the deamination of methylated cytosine to thymine, leading to a lower GC content.
3. Repeat TP will differ between tissues. This might be expected if there is upregulation of repeat transcription associated with their amplification in particular tissues, especially those that are in the germ line. Such activity might be expected in poly‐A RNA libraries. In ribo‐depleted RNA libraries, differences in TP of repeats between tissues may be less pronounced because the silencing of repeats by RdDM may be ubiquitous to all cell types.

## 2 MATERIALS AND METHODS

### 2.1 Datasets used in the analysis

The DNA sequences used to characterize genomic repeats with RepeatExplorer2 were downloaded from NCBI (see Table S1 for details) and comprised paired‐end Illumina NovaSeq 6000 sequence data with reads of 151 bp length from genomic DNA of *S. viridis* ‘A10’ (SRR10051273) (Mamidi et al., 2020).

For the RNA sequencing conducted here, *S. viridis* ‘A10’ seeds were washed with distilled water and sterilized by incubating in a solution of 20% sodium hypochlorite and 0.1% Tween 20. Seeds were germinated in petri dishes in MS medium for 5 days, followed by 7 days in plastic pots. After root system establishment, the seedlings were transferred to pots containing a mixture of substrate and sand (1:1) and grown in a plant chamber in the Federal University of Juiz de Fora (Brazil), under a photoperiod of 16/8 h (light/dark), temperature 25 ± 2°C for at least 20 days. RNA from c. 100 mg of leaf material was extracted with the RNeasy Plant Mini Kit (74904 Qiagen).

Ribo‐depleted RNA libraries were prepared for direct sequencing using Invitrogen's RiboMinus Plant Kit for RNA‐Seq (cat. A10830‐08), which uses labelled oligos against ribosomal sequences to deplete unwanted ribosomal RNA (rRNA) transcripts. Isolated RNA was purified and concentrated using the Monarch RNA Cleanup Kit. Poly‐A RNA libraries were prepared for direct sequencing using New England Biolab's NEBNext poly(A) mRNA magnetic isolation module (NEB E7490; 7 min. fragmentation time). Direct sequencing of the ribo‐depleted and poly‐A libraries, each with three biological replicates, was performed using Illumina NovaSeq 6000 (Genomic Centre, Queen Mary University of London), generating 150 bp paired‐end reads.

Further RNA libraries were downloaded from NCBI, sourced from two previously published experiments, each with three biological replicates (see Table S1 for details). These were: (i) paired‐end Illumina HiSeq 2000 sequence data of ribo‐depleted transcriptome libraries of *S. viridis* ‘A10’ with read lengths of 101 bp. We analyzed the data generated from stem tissue (the region where new tillers are produced) and crown tissue (the region where new roots are produced), sampled 9 days after sowing. The libraries and the experimental conditions are described in Sebastian et al. (2016); and (ii) single‐end Illumina HiSeq 2500 sequence data from poly‐A transcriptome libraries with read lengths of 100 bp prepared from inflorescence primordia of wild‐type material sampled 15 days after sowing. Details are described in Yang et al. (2018).

### 2.2 Analysis of repeats in the genome using RepeatExplorer2

All reads from the sequencing conducted here had adapter sequences removed using Trimmomatic v.0.39 before analysis. Sequence read quality control was evaluated using FastQC. Reads corresponding to 0.4% of the genome that had passed the quality control threshold (Phred score > 33) were trimmed to 151 bp in Trimmomatic v.0.39 (Bolger et al., 2014). Paired‐end reads from Sebastian et al. (2016, 101 bp) and single‐end reads from Yang et al. (2018, 100 bp) were used.

Repeats were characterized using the RepeatExplorer2 pipeline (Novák et al., 2010; Novák, Neumann & Macas, 2020), implemented on the Galaxy server (https://repeatexplorer‐elixir.cerit‐sc.cz). Briefly, using an all‐to‐all BLAST analysis, RepeatExplorer2 (pipeline version: 0.3.8‐451[9d65fb1]) clusters reads that are at least 90% similar over at least 55% of the sequence length to identify, quantify, and de novo annotate repeats (Figure S1). In addition, based on protein domains (protein database Viridiplantae v2.2.fasta) and other DNA databases, including those in RepeatExplorer2, we identified known TEs. TEs were classified according to the REXdb classification system (Neumann et al., 2019).

The GP of each repeat cluster containing more than 150 reads (termed here “repeat top clusters”) was calculated as the total number of reads in the repeat cluster divided by the total number of reads analyzed (excluding reads from clusters identified as being derived from mitochondria or plastids).

### 2.3 Characterizing sequence similarity and GC content of repeats in the genome

The reads of each cluster generated by RepeatExplorer2 were used in all‐to‐all BLAST searches to generate pairwise similarity scores. The frequency distribution of the pairwise similarity scores was used to estimate the repeat similarity scores (=modal value of the scores for reads with ≥80% similarity in each cluster), using a custom Perl script (see examples in Figure S2) as in Wang et al. (2019). The Perl script was used to filter out self‐hits and reciprocal hits from the BLAST results. The final modal value for each cluster and the linear models (Table S2, Figures S3–S5) were calculated in R Studio (http://www.rstudio.com) (R Core Team, 2020). The GC content of all reads in each of the repeat top clusters was estimated using an adapted Python script (Meneghin, 2009).

### 2.4 Pipeline for detecting expressed repeats in the transcriptome

The RNA‐seq data (see Table S1) were evaluated using FastQC, and those that passed the quality control threshold (Phred score > 33) were trimmed to 151 bp in Trimmomatic v.0.39 to remove Illumina adaptors (Bolger et al., 2014). The number of reads in each library that passed thresholds for quality after trimming are given in Table S1.

The pipeline used to identify and quantify reads in the transcriptome using RepeatExplorer2 is summarized in Figure S6. RepeatExplorer2 generates a library of contigs that are consensus sequences for the genomic repeats that comprise each repeat cluster. The files “contigs.fasta” for each cluster from RepeatExplorer2 were merged to produce a library of repeat contigs for mapping. This repeat contig library was used as the reference to map reads from each of the transcriptome libraries using Bowtie2 (Langmead & Salzberg, 2012). The threshold for mapping was 90% similarity over at least 55% of the sequence length. By default, Bowtie2 performs end‐to‐end read alignment. Samtools (Li et al., 2009) was used to save the output files (.bam and .sam formats), and the total number of transcript reads that mapped once to any of the contigs in a cluster was calculated and used for further analysis. This research utilized Queen Mary's Apocrita HPC facility, supported by QMUL Research‐IT (https://doi.org/10.5281/zenodo.43804).

### 2.5 Calculating the transcript proportion of repeats in the transcriptome

To calculate the TP of each repeat, the total number of mapped transcript reads (RNA‐seq) was mapped to the RepeatExplorer2 contigs derived from the DNA sequence reads (genome sequencing), excluding any RNA reads that mapped to repeat clusters comprising ribosomal DNA (rDNA) or organelle sequences. Some repeat clusters contained a proportion of DNA sequences of organellar origin (CL3, CL35, CL56, CL60, CL72, CL75, CL97). While some reads might represent examples of the natural integration of organellar DNA into the nuclear genome, we nevertheless removed them as they are not relevant to this analysis. Some cluster reads with a proportion of tRNA sequences were also removed (CL2, CL7, CL8, CL18, CL19, CL22, CL25, CL26, CL40, CL41, CL60, CL81, CL99). The RNA‐seq reads that mapped to repeat clusters were used to calculate the TP of repeats in the ribo‐depleted and poly‐A RNA libraries. Repeats were classified at the levels of repeat superfamily and individual repeat lineages within these superfamilies (Tables S3–S10). Finally, the total number of mapped RNA‐seq reads for each repeat type (i.e., repeat superfamily, e.g., all Ty3/gypsy elements) or individual repeat lineages (e.g., Athila elements) was summed together and divided by the total number of RNA‐seq reads analyzed in the library (excluding rRNA and organelle reads) and expressed as a percentage.

### 2.6 Mapping repeats and transcribed repeats to the *S. viridis* whole genome assembly

To compare the results obtained using the genome skimming data (see above, = RepeatExplorer‐identified repeats) with those using a whole genome assembly as input to characterize the repeats (see below, = DANTE‐identified LTR retroelements), we took advantage of the platinum‐grade chromosome‐level whole genome assembly available for *S. viridis* ‘A10’ (Genebank: GCF_005286985.1) (Mamidi et al., 2020).

First, DNA sequence reads from the genome assembly were used as input to identify and annotate LTR retroelements (LTR‐RT) using the DANTE v0.1.8 (https://doi.org/10.5281/zenodo.8183566) and DANTE_LTR v0.3.5 pipeline (https://doi.org/10.5281/zenodo.10213785) available on the RepeatExplorer Galaxy server (https://doi.org/10.1038/s41596‐020‐0400‐y).

The sequences of the identified LTR‐RT elements were used to create a custom library of LTR‐RT elements using “he “dante_ltr_to_lib”ary” script from the DANTE_LTR repository and used as a library for RepeatMasker search to annotate the LTR retroelements using a similarity‐based approach. The RepeatMasker search was performed on the RepeatExplorer Galaxy server with opti“ns “‐xsmall ‐no_is ‐e ”cbi”. The output enables both complete and incomplete LTR retroelements to be identified based on the presence or absence of the following three components: LTRs, primer binding site (PBS), and target site duplication (TSD). Complete elements had all three components, while incomplete elements lacked at least one of these. The GFF file following annotation served as a reference for counting reads in the .bam files using HTseqCount. We filtered out repeats with at least 100 mapped reads and inspected each location in the genome assembly using integrative genomic viewer (IGV) to determine their proximity to genes or their location within genes, for example, within introns. Additionally, we identified specific repeats and investigated those that were differentially expressed with Deseq2 (RPM normalization). In the heatmaps, transcript abundance was normalized by row.

In addition, we mapped the repeat RNA‐seq reads identified in the poly‐A and ribo‐depleted RNA libraries to the whole genome assembly using Bowtie2 (Dobin et al., 2013). The .bam files were converted to .bed and .gff3 files using bedtools (Quinlan & Hall, 2010) and genometools (Gremme et al., 2013).

### 2.7 Linear regression models

Multiple linear regression models were tested to analyze the relationships between the TP of each repeat and the following factors: GP, GC content, sequence similarity (%), tissue type, experimental condition, and repeat type. In all models, we analyzed three replicates. To meet statistical assumptions inherent to the models, some variables were log‐transformed to generate a normal distribution of values. In these cases, clusters that had a zero TP value (*y*‐axis) were not included. All linear regressions and statistical analyses were performed using R version 4.0.2 (R Core Team, 2020). Associated figures were generated using RStudio and Photoshop CC version 2012.0.1 (Adobe Systems).

All scripts are stored in GitHub (https://github.com/ana‐franco‐bio/Repeats_transcriptome).

## 3 RESULTS

### 3.1 Characterization of repetitive elements in the genome of *S. viridis*

Of the 74,498,042 paired‐end reads from the leaf genomic DNA library (Table S1), 1,579,633 (i.e., 2.1%) were analyzed by RepeatExplorer2. A total of 208 repeat clusters were generated, and after removing 24 clusters containing plastid and mitochondrial sequences, 184 clusters of repeats made up a GP of 35.25% (Table 1). Discounting nine clusters containing reads of rDNA left 175 repeat clusters comprising a GP of 31.67% (Table 1). Inclusion of rDNA gave a GP of 35.25% (Table 1). Graphically displayed examples of repeat clusters are shown in Figure S1.

**TABLE 1:** Genome proportions (GPs), repeat sequence similarity score ranges, and GC content identified in *Setaria viridis* ‘A10’ using RepeatExplorer2.

Repeat class/superfamily	Repeat lineage	GP (%)	Range of mode sequence similarity scores (%)	GC content range (%)	Number of clusters
**Class I—RNA retroelements**					
**Ty3/gypsy**		9.03	86.86–99.24	25–61	39
	Athila	0.68	89.01–91.98	36–44	6
	CRM	1.12	88.05–99.24	33–48	7
	Ogre	1.76	86.86–91.91	39–50	5
	Reina	0.07	86.95–89.81	25–36	3
	Retand	2.57	86.9–93.07	40–61	11
	Tekay	2.83	91.54–98.79	39–59	7
**Ty1/copia**		4.01	89.95–98.53	27–61	24
	Ale	0.1	89.95–97	44–61	4
	Angela	2.42	91.37–98.53	27–52	9
	Bianca	0.03	90.95	33	1
	Ikeros	0.17	92.37–96.57	37–43	2
	Ivana	0.5	91.92–94.21	47–60	2
	SIRE	0.52	90.7–92.09	40–44	4
	TAR	0.2	96.64	43	1
	Tork	0.06	96.15	44	1
**LINE**		0.05	92.23	43	1
**Unclassified LTR**		5.73	90.25–95.98	38–61	9
**Pararetrovirus**		0.01	94.69	30	1
**Class II—DNA transposons**		3.39	90.06–98.54	33–47	34
	CACTA	2.02	90.34–97.72	38–45	7
	Helitron	0.02	94.4–96.06	41–47	2
	Mariner	0.02	98.42	39	1
	Mutator	0.79	90.25–97.09	33–46	7
	Harbinger	0.31	90.06–98.64	38–45	10
	hAT	0.23	93.72–98.4	34–44	7
**Satellite repeats**		3.86	85.77–96.78	42–54	3
**Unknown repeats**		5.59	87.31–99.83	26–59	64
**Total**		31.67			175
**45S rDNA**		3.52	99.98–100	50–62	8
**5S rDNA**		0.06	99.99	55	1
**Total repeats**		35.25			184

Abbreviations: GC, guanine and cytosine; LINE, long interspersed nuclear element; LTR, long terminal repeat.

**FIGURE 1:**
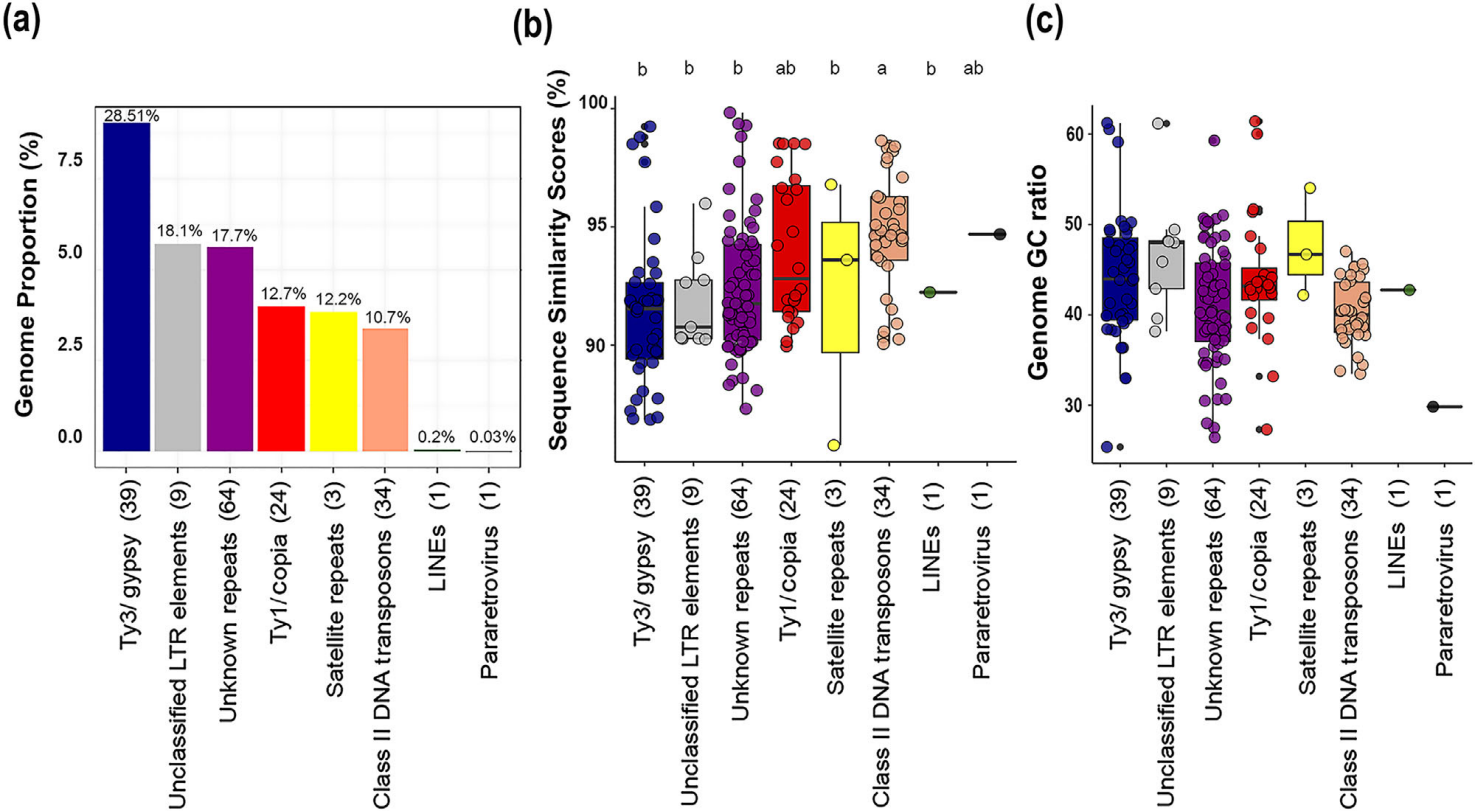
(a) Bar chart showing the genome proportion (GP) of different lineages of repetitive elements in *Setaria viridis* ‘A10’ detected by RepeatExplorer2 (LINEs, long interspersed nuclear elements). The numbers above each bar represent the proportion each repeat type occupies out of the total repeat fraction minus rDNA (i.e., 31.67% GP). Box plots showing: (b) the repeat sequence similarity scores for repeat clusters in each lineage (repeat lineages with the same letter code are not significantly different; Tukey multiple comparison of means, *p* < 0.05), (c) Repeat cluster guanine and cytosine (GC) guanine and cytosine [content (there were no significant differences between the different repeat lineages). The circular points represent individual clusters. The box plots show means, interquartile ranges, and maximum and minimum values. The number in brackets after each repeat family represents the total number of clusters analyzed.

Ty3/gypsy elements were the most abundant repeat superfamily (9.03% GP), representing 28.5% of the total repeat fraction of the genome (minus rDNA). The next most abundant repeats were unclassified LTRs (5.73% GP and 18.1% of the total repeat fraction) and Ty1/copia sequences (4.01% GP and 12.7% of the total repeat fraction, Figure 1a, Table 1). Satellite repeats and DNA transposons were also abundant, comprising GPs of 3.86% and 3.39%, respectively, which represent 12.2% and 10.7% of the total repeat fraction, respectively (Figure 1a). The individual cluster with the highest GP (cluster 1, 3.67% GP) was a satellite repeat, as interpreted from the star‐shaped cluster graph generated by RepeatExplorer2.

Using all‐to‐all BLAST of the reads in each repeat cluster enabled us to calculate the mode sequence similarity score (as in Figure S2). Repeats with high scores are considered to be derived from recently amplified repeats that have few mutations since their amplification, while repeats with lower scores are predicted to correspond to more ancient insertions that have degraded by mutation. Ty3/gypsy, unknown repeats, Ty1/copia and satellites (the latter with just three clusters) all showed considerable variation in repeat similarity scores between clusters (Table 1). Only DNA transposons had significantly higher repeat similarity scores (Tukey multiple comparison of means, *p* < 0.05) compared with other repeat types (Figure 1b).

The GC content varied considerably between repeat clusters, from 25% GC (cluster 151) for a Ty3/gypsy Reina element to 61% for a Ty1/copia Ale element (cluster 110) and a Ty3/gypsy Retand element (cluster 51) (Table 1, Figure 1c). There were no significant differences in the GC content between the broadest categories of repeats, for example, Ty1/copia and Ty3/gypsy (i.e., different members of “Repeat Class/Superfamily” in Table 1 [Tukey multiple comparison of means, *p* > 0.05]).

Using linear regression models, we found that repeat GP was positively correlated with: (i) GC content for Ty3/gypsy elements (Figure S3a, Table S2b, adjusted *R* ^2^ = 0.156, *p* < 0.001) and (ii) modal repeat sequence similarity for Ty1/copia elements (Figure S3b, Table S2f, adjusted *R* ^2^ = 0.417, *p* < 0.005). Thus, the most abundant repeats in the genome were either GC‐rich (Ty3/gypsy) or had high sequence similarity scores (Ty1/copia). No relationships were observed between GC content and repeat similarity (Table S2g), GP and GC content (Table S2a), or GP and repeat similarity (Table S2d) for DNA transposons.

**FIGURE 2:**
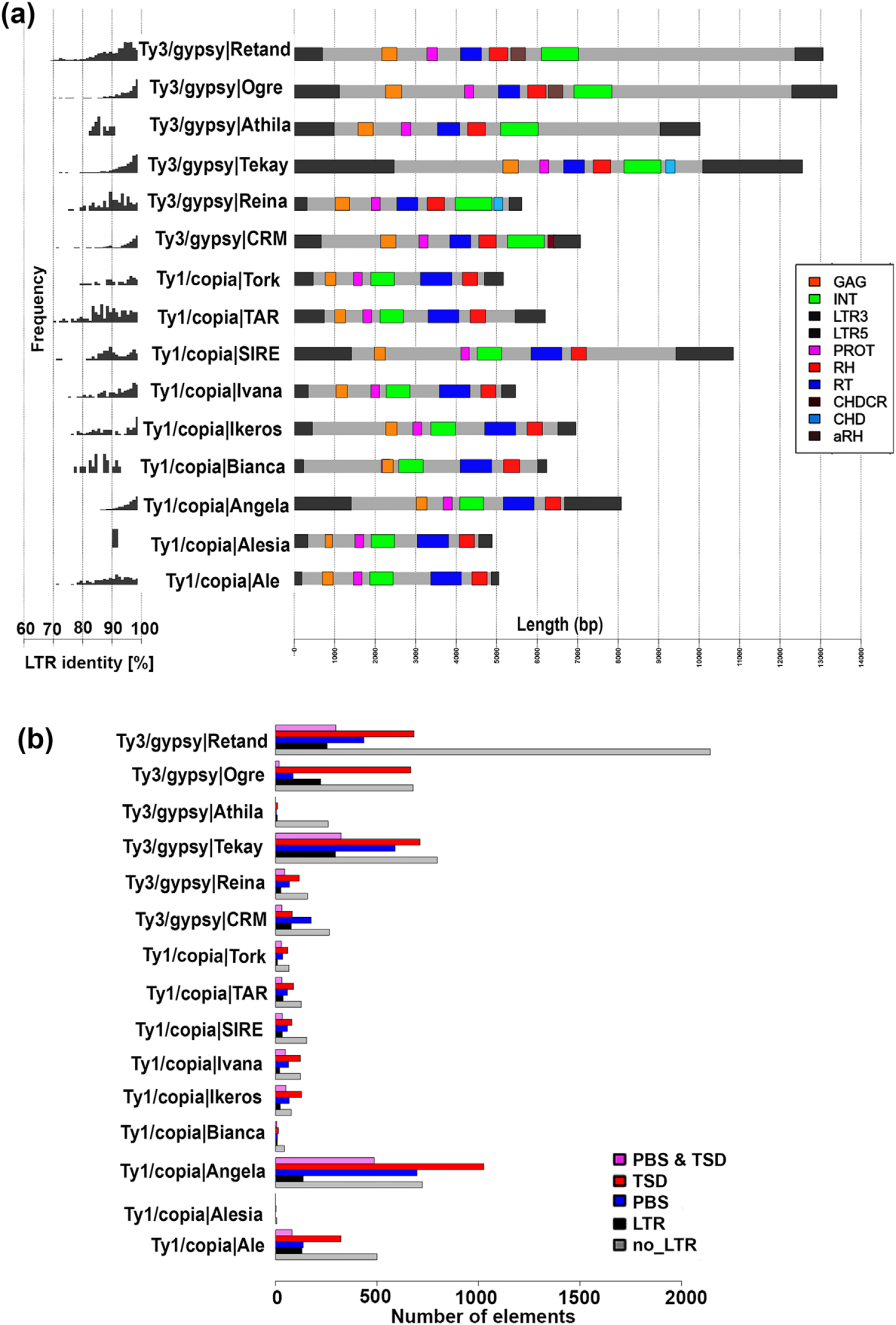
Structure of full‐length long terminal repeat (LTR) retroelements in the *Setaria viridis* genome identified using DANTE annotation. (a) Frequency of sequence identities (%) between the paired LTRs (LTR3’/LTR5’) across the genome together with the consensus structure and length for each identified LTR retroelement. The protein domains identified were GAG: capsid, INT: integrase, PROT: protease, rH: RNaseH, RT: reverse transcriptase, CHDCR: chromodomain of centromeric retrotransposons, CHD: chromodomain, and aRH: archeal ribonuclease H (see key for color code). (b) Copy numbers for the different types of incomplete LTR retroelements present in the genome assembly of *S. viridis* identified using DANTE: PBS: primer binding site, TSD: target site duplication.

In total, 4337 repeats were identified by DANTE in the genome assembly as complete LTR retroelements (i.e., they have both LTRs and a TSD and PBS) and 171,715 repeats as incomplete elements (Table S3), a ratio of ∼40 incomplete to complete elements. Notably, the retroelements Ty3/gypsy Retand, Ty3/gypsy Tekay, and Ty1/copia Angela had the highest numbers of complete elements in the genome (Table S3). These three elements were also the most abundant elements in the RepeatExplorer2 analysis of the genome (i.e., 2.57%, 2.83%, and 2.42% GP, respectively; Table 1). The LTRs with the highest sequence identities were Ty3/gypsy Ogre, Tekay, and CRM and Ty1/copia Angela (Figure 2a, left panel).

### 3.2 Comparison of repeat TP in ribo‐depleted and poly‐A libraries identified by either RepeatExplorer2 or DANTE

**FIGURE 3:**
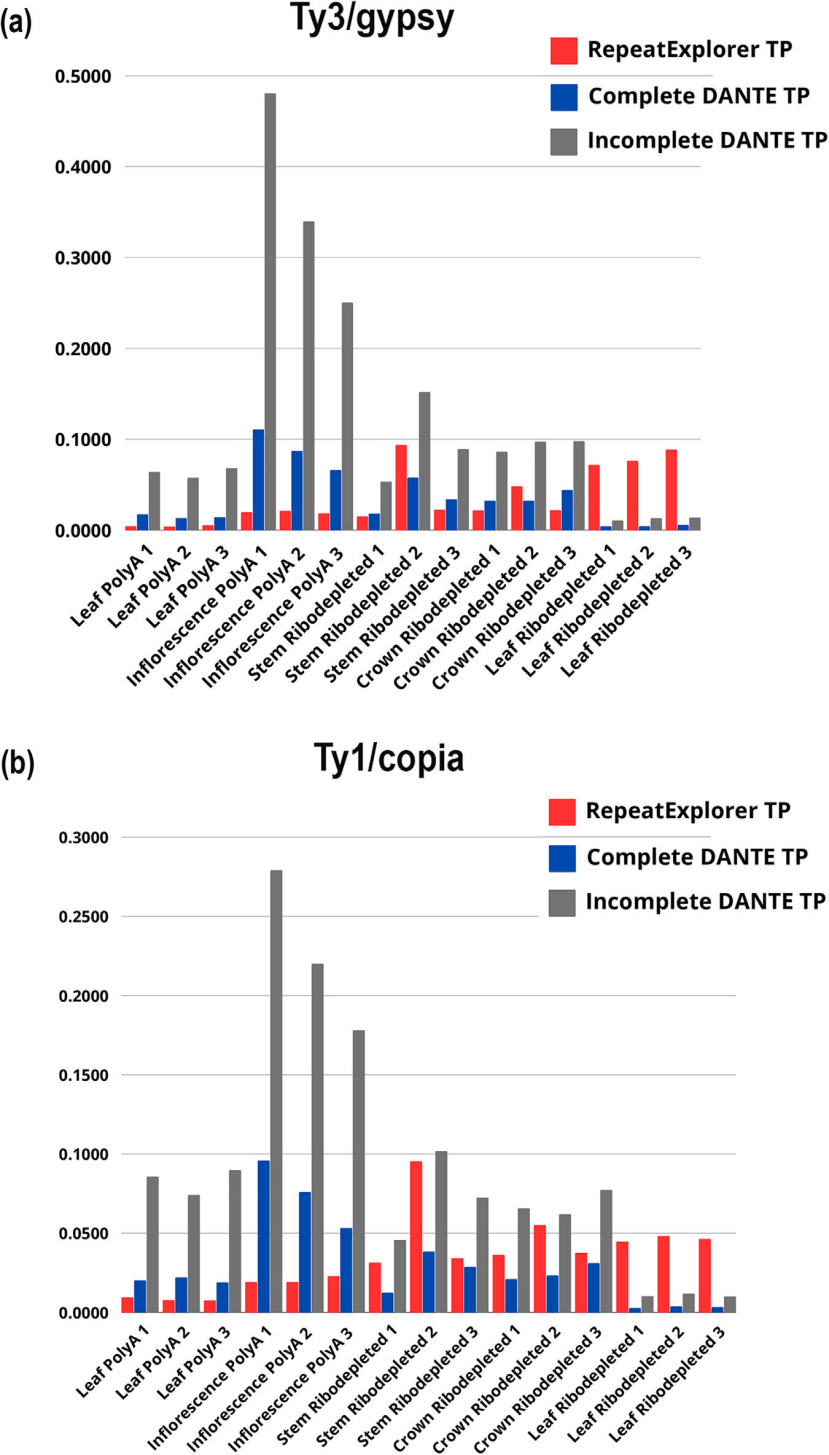
The transcriptome proportions (TP,%) of complete and incomplete Ty3/gypsy and Ty1/copia elements as detected by DANTE in various tissues and library types of *Setaria viridis* estimated by mapping RNA‐seq reads to retroelements. To the same RNA libraries, RepeatExplorer2 detects repeats without categorization to complete or incomplete elements.

We compared the TPs of RNA‐seq reads that aligned to Ty3/gypsy and Ty1/copia LTR retroelements identified by DANTE (i.e., DANTE‐identified LTR retroelements) with those that aligned to the repeat contigs identified by RepeatExplorer2 (=RepeatExplorer‐identified retroelements; Table 2). In all three ribo‐depleted libraries, the mean TP (%) of RepeatExplorer‐identified LTR retroelements was higher than the TP (%) for complete elements identified by DANTE (Table 2). However, when both complete and incomplete Ty3/gypsy and Ty1/copia LTR retroelements identified by DANTE were added together, the total TP (%) was mostly higher for DANTE‐identified LTR retroelements than RepeatExplorer‐identified LTR retroelements in both poly‐A and ribo‐depleted libraries, with the exception of the leaf ribo‐depleted transcriptomes, where the reverse was the case (Table 2). In these ribo‐depleted leaf transcriptomes, the RNA‐seq reads that aligned to all the DANTE‐identified Ty3/gypsy and Ty1/copia LTR retroelements (both complete and incomplete) were substantially underrepresented compared to the reads that aligned to the RepeatExplorer‐identified repeats (Figure 3).

**TABLE 2:** Comparisons of mean transcript proportion (TP) of all Ty1/copia and Ty3/gypsy retroelements in five RNA libraries (ribo = ribo‐depleted RNA library; poly‐A = poly‐A RNA library) analyzed using repeats identified either by RepeatExplorer2 or DANTE annotation.

		RepeatExplorer‐identified repeats	DANTE‐identified LTR retroelements			
Tissue	Library type	Mean TP of all LTR retroelements (%)	Mean TP of complete Ty1/copia and Ty3/gypsy retroelements (%)	Mean TP of incomplete Ty1/copia and Ty3/gypsy retroelements (%)	Mean TP for all Ty1/copia and Ty3/gypsy retroelements (%)	Ratio of incomplete:complete TP of Ty1/copia and Ty3/gypsy retroelements
Leaf	Poly‐A	0.013	0.035	0.146	0.182	0.806
Leaf	Ribo	0.125	0.008	0.023	0.031	0.739
Crown	Ribo	0.074	0.061	0.162	0.224	0.725
Stem	Ribo	0.097	0.063	0.172	0.235	0.731
Inflorescence	Poly‐A	0.040	0.163	0.583	0.746	0.781

Abbreviation: LTR, long terminal repeat.

The highest mean TP estimated for the DANTE‐identified complete and incomplete LTR retroelements was in poly‐A libraries, where they comprised a total mean TP of ∼0.75% of the inflorescence transcriptomes. In ribo‐depleted libraries, the highest DANTE‐identified mean repeat TP was 0.24% in stem transcriptomes (Table 2).

Using the integrative genomic viewer (IGV), we conducted a manual inspection of the chromosomal distribution of 210 complete LTR retroelements identified by DANTE that had a minimum of 100 mapped RNA‐seq reads from either the poly‐A or ribo‐depleted libraries to determine whether they were positioned adjacent to genes, within intronic regions, or in other genomic areas. Among these repeats, 142 were located in intronic regions (e.g., Ty1/copia Ale and Ivana elements, see Figure S7d), while the remaining 68 were situated between genes (e.g., Ty3/gypsy CRM element on chromosome 2, see Figure S7c).

We determined if there was differential expression of the 68 non‐intronic LTR retroelements identified using DANTE. In the leaf transcriptomes, 15 repeats exhibited differential expression between ribo‐depleted and poly‐A libraries, with ribo‐depleted libraries showing an upregulation in transcription of 11 LTR retroelements compared to poly‐A libraries (Figure S7b), while four elements showed the reverse. For example, a Ty1/copia Ivana on chromosome 8 and a Ty3/gypsy Tekay element on chromosome 9 (TE_00000924_Chr8 _Ivana and TE_00000780_Chr9 Tekay, respectively) were both highly expressed in ribo‐depleted libraries (Figure S7b). In contrast, a Ty1/copia Ikeros element on chromosome 8 (TE_00001047_Chr8_Ikeros) was upregulated in poly‐A libraries (Figure S7b).

### 3.3 Relationship between repeat TP and repeat GP in ribo‐depleted and poly‐A libraries

**FIGURE 4:**
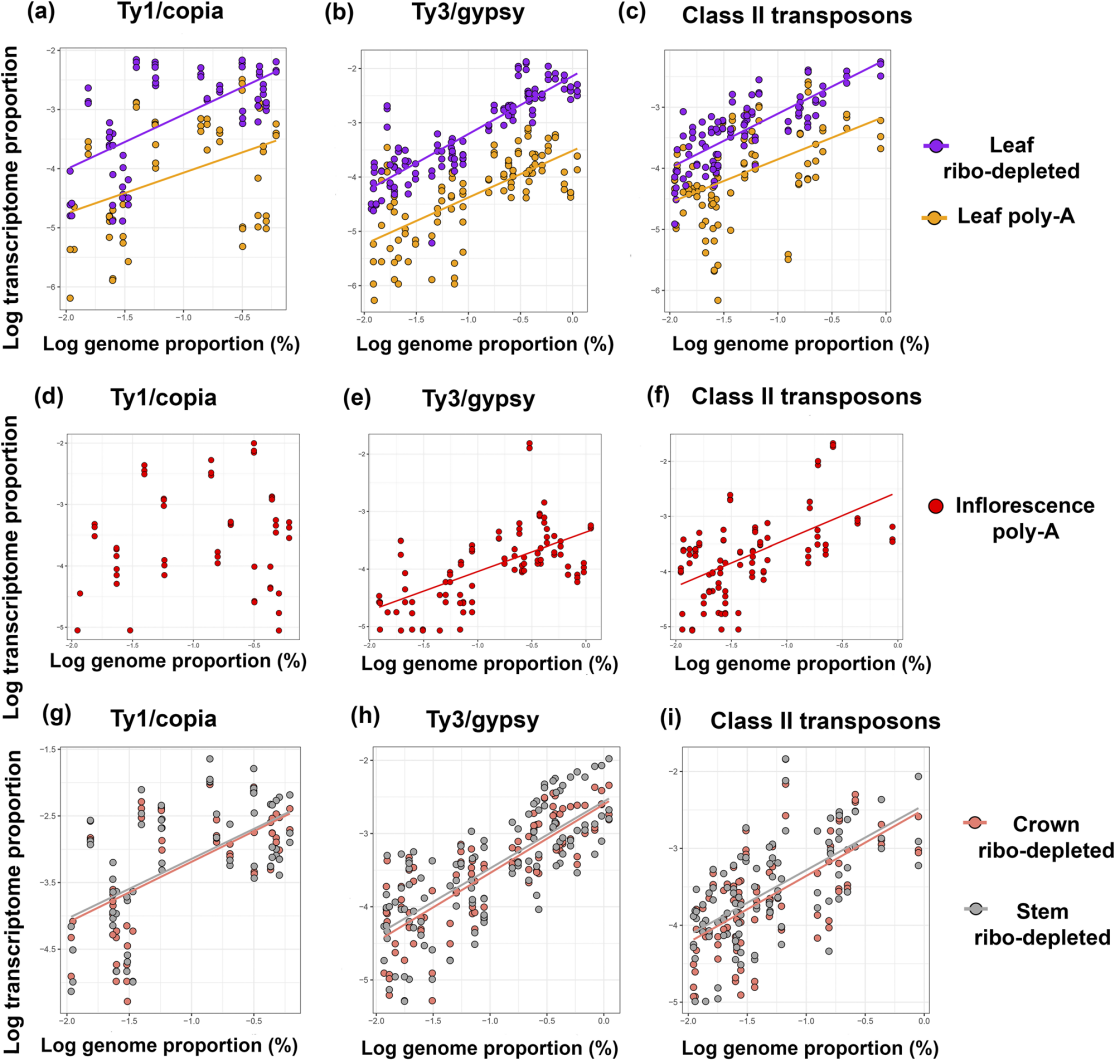
Scatterplots showing the relationship between transcriptome proportion and genome proportion in different tissues of *Setaria viridis*. (a, d, and g) Ty1/copia, (b, e, and h) Ty3/gypsy elements, and (c, f, and i) class II DNA transposons from (a–c) leaf ribo‐depleted and poly‐A libraries, (d–f) inflorescence poly‐A libraries, and (g–i) crown and stem ribo‐depleted ribo‐depleted libraries. Each data point corresponds to one repeat cluster (three replicates of each RNA library mapped). For coefficients and slopes of (a–c), see Table S6a–c; for (d–f), see Table S7a–c; and for (g–i), see Table S7g–i.

To address hypothesis 1 that the TP of individual repeat types reflects their abundance in the genome, especially in ribo‐depleted libraries, we quantified the TP of repeats identified by RepeatExplorer2 in the ribo‐depleted and poly‐A libraries of leaf (Tables S4 and S5, respectively). In both transcriptome types, we observed a significant positive relationship between repeat TP and GP for Ty3/gypsy and Ty1/copia retroelements and for DNA transposons (Figure 4a–c, *p* < 0.0001, Table S6a–c). This relationship was strongest for Ty3/gypsy in the ribo‐depleted transcriptome (*R* ^2^ = 0.742, Table S6b). Indeed, expression levels were significantly higher in ribo‐depleted libraries compared with poly‐A libraries for all repeat types analyzed (Table 3, Tables S4 and S5). In the leaf poly‐A transcriptome, the TP of Ty1/copia and DNA transposons (both TP = 0.008) exceeded the TP of Ty3/gypsy elements (TP = 0.005%) (Table 3, Table S5). However, in the ribo‐depleted libraries, the TP of Ty3/gypsy elements (TP = 0.08%) exceeded the TP of Ty1/copia (TP = 0.05%) and DNA transposons (TP = 0.03%) (Table 3).

**TABLE 3:** Mean transcriptome proportion (TP) of each repeat class and superfamily identified by RepeatExplorer2 from different tissues (three replicates of each) of ribo‐depleted and poly‐A libraries in *Setaria viridis* ‘A10’.

	Transcriptome proportion (%)				
Repeat	Ribo‐depleted libraries			Poly‐A libraries	
Class/superfamily	Crown	Stem	Leaf	Leaf	Inflorescence
**Class I—RNA retroelements**					
Ty3/gypsy	0.0306	0.0438	0.0788	0.0046	0.0200
Ty1/copia	0.0430	0.0537	0.0464	0.0083	0.0200
LINEs	0.0093	0.0088	0.0051	0.0022	0.0090
Unclassified LTR	0.0429	0.0422	0.0725	0.0099	0.0150
Pararetrovirus	0.0004	0.0002	0.0000	0.0000	0.0000
**Class II—DNA transposons**	0.0259	0.0291	0.0282	0.0084	0.0390
**Satellite repeats**	0.0010	0.0044	0.0086	0.0000	0.0000
**Unknown repeats**	0.0570	0.0653	0.0700	0.0387	0.0670
**Total**	0.210	0.248	0.310	0.072	0.170

Abbreviations: LTR, long terminal repeat; LINE, long interspersed nuclear element.

The ribo‐depleted libraries from crown and stem tissues (Tables S9 and S10, respectively) showed a positive relationship between TP and GP for Ty3/gypsy, Ty1/copia, and DNA transposons (*p* < 0.0001, Table S7g–i, Figure 4g–i). Nevertheless, despite Ty3/gypsy elements being the most abundant repeat type in the genome (Table 1), Ty1/copia elements were typically the most abundant in the poly‐A transcriptome (Table 3). In both crown and stem ribo‐depleted transcriptomes, the TPs from Ty3/gypsy, Ty1/copia, and unclassified LTR repeats were higher than the TPs from DNA transposons (Table 3). Satellite repeats showed very low TP in both these tissues, despite comprising nearly 4% of the genome.

In inflorescence tissue, there was a positive relationship between GP and TP in poly‐A libraries for Ty3/gypsy and DNA transposons, but not for Ty1/copia (Figure 4d–f, Tables S7a–c and S8). Comparing between tissue types, we observed that DNA transposons occurred in a higher proportion in the poly‐A transcriptome from inflorescence tissue (TP = 0.039%) than in leaf poly‐A (TP = 0.008%) or leaf ribo‐depleted (TP = 0.028%) transcriptomes (Table 3, Tables S4, S5, and S8).

### 3.4 Relationships between repeat TP and repeat GC and repeat sequence similarities

**FIGURE 5:**
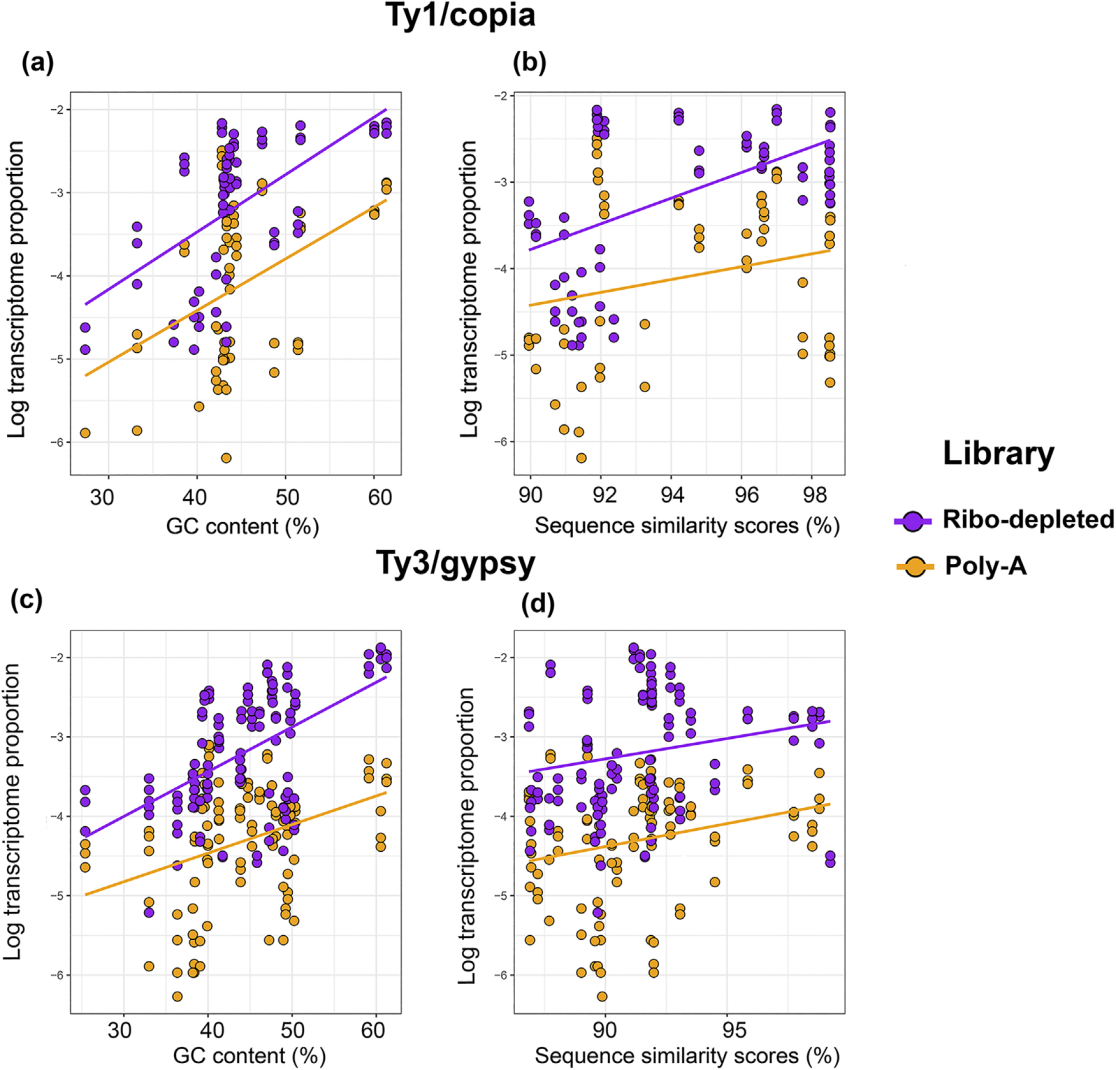
Scatterplot showing the relationship between the transcriptome proportion (TP) of Ty1/copia (a and b) and Ty3/gypsy repeats (c and d) in ribo‐depleted and poly‐A libraries of leaf transcriptomes and their genomic characteristics (i.e., guanine and cytosine [GC] content and sequence similarity scores). For coefficients and slopes, see Table S6d,e.

We used linear regression models to address hypothesis 2 that the highest repeat TP comes from genomic repeats with a high GC content and/or sequence similarities between copies. We observed that there were indeed significant positive relationships between repeat TP for repeats identified by RepeatExplorer2 and GC content for both Ty3/gypsy and Ty1/copia repeats in all library types and tissues (i.e., poly‐A libraries of leaf and inflorescence tissues; ribo‐depleted libraries of leaf, crown, and stem tissues, Figure 5a,b, Figures S4a,b and S5a,b). DNA transposons showed correlations between repeat TP and GC content only in the poly‐A libraries of inflorescence tissues (Figure S4c). For coefficients and slopes of the linear regression models, see Tables S6 and S7.

The relationship between repeat TP and repeat sequence similarity varied between RNA libraries. Only Ty1/copia showed significant positive relationships between these variables in all library types (Figure 5b, Figure S5b), except the poly‐A library of inflorescence tissues (Figure S4d). For Ty3/gypsy elements, a positive relationship was found between repeat TP and repeat sequence similarity in leaf poly‐A and ribo‐depleted libraries (Figure 5d) but not in the other transcriptome libraries analyzed (Figures S4e and S5d). We detected no significant correlations between TP and sequence similarity for DNA transposons in any transcriptome analyzed.

### 3.5 Contrasting repeat transcription profiles in different tissues

We used the repeats identified using RepeatExplorer2 and DANTE to address hypothesis 3 that the TP of repeats differs between tissues by comparing the repeat TP in libraries of *S. viridis* leaf, crown, stem, and inflorescence tissues.

**FIGURE 6:**
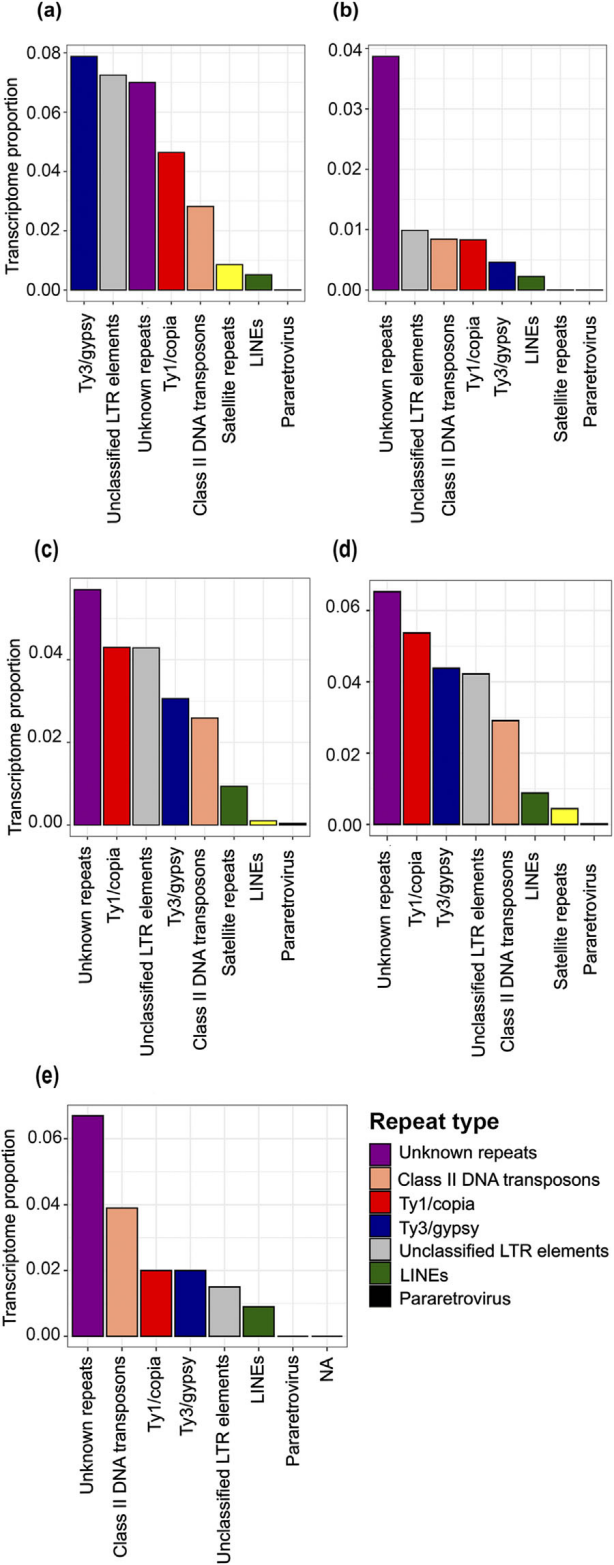
Transcriptome proportions (percentage of library) of different repeat lineages identified by RepeatExplorer2 in *S. viridis* transcriptomes showing differences in their abundance between tissues and types of transcriptomes analyzed. (a, c, and d) Ribo‐depleted libraries and (b and e) poly‐A libraries, from (a and b) leaf, (c) crown, (d) stem, and (e) inflorescence tissues (*n* = 3 for each transcriptome). Total TPs for each library and repeat lineage are shown in Table 3: (a) leaf ribo‐depleted = 0.31%, (b) leaf poly‐A = 0.07%, (c) crown ribo‐depleted = 0.21%, (d) stem ribo‐depleted = 0.25%, and (e) inflorescence poly‐A = 0.17%. LTR, long terminal repeat; LINE, long interspersed nuclear element.

In leaf ribo‐depleted libraries, the sum of all RepeatExplorer‐identified repeats had a TP of 0.31% (Table 3), with the most abundant repeat transcribed being Ty3/gypsy elements, followed by unclassified LTR elements, unknown repeats, Ty1/copia, and DNA transposons (Figure 6a). We observed no significant differences in the TP of all RepeatExplorer2‐identified repeats in crown (0.21%) and stem (0.25%) ribo‐depleted transcriptomes (Table 3), where the most expressed repeats were Ty1/copia elements, followed by Ty3/gypsy, with DNA transposons, LINEs, satellites, and pararetrovirus sequences (Figure 6c,d).

Similarly, of the transcribed RepeatExplorer‐identified repeats in the poly‐A libraries, a large fraction of the leaf transcriptome was from unknown repeats, and both DNA transposons and Ty1/copia were more expressed than Ty3/gypsy (Figure 6b). Nevertheless, there were significant differences in repeat content in the poly‐A libraries between leaf and inflorescence tissues, with the mean total RepeatExplorer‐identified repeats for inflorescence transcriptomes (0.17%) being more than twice that observed for leaf transcriptomes (0.072%, Table 3). The inflorescence poly‐A transcriptome also had highly abundant DNA transposons (Figures 6e), exceeding what was observed in the leaf poly‐A transcriptome and more abundant in the transcriptome than the identified Ty1/copia and Ty3/gypsy elements, despite DNA transposons being less abundant than these retroelements in the genome (Table 1).

Analysis of the RNA reads mapped against the DANTE‐annotated genome also showed a higher TP of reads that mapped to both complete and incomplete LTR retroelements in inflorescence poly‐A libraries (0.75%) compared with leaf poly‐A libraries (0.18%, Table 2).

## 4 DISCUSSION

### 4.1 The repeat content of the *S. viridis* genome

Our analysis of the repeat content of *S. viridis* ‘A10’ using RepeatExplorer2 estimated that c. 35% of the genome (including rDNA) comprised repeats. This is similar to previous estimates of c. 40%–46% in closely related *S. italica* assessed from genome annotations (Bennetzen et al., 2012; Thielen et al., 2020; Zhang et al., 2012), a species with a similar genome size (0.49 Gb/1*C*). The data are also consistent with the relationships between repeat GP and genome size reported in Novák, Guignard et al. (2020). The lower abundance of LTR retroelements in the RepeatExplorer2 analysis (i.e., RepeatExplorer‐identified repeats) in *S. viridis* compared with the analyses of the DANTE‐identified LTR retroelements in the annotated whole genome assembly of *S. viridis* may be because RepeatExplorer2 does not detect low‐copy (less than c. 200 copies in the genome) repeat sequences. Indeed, comparing repeat GP between species analyzed with different methods is problematic, a reason why Novák, Guignard et al. (2020) used the same approach to measure repeat content in over 100 species differing in genome size to ensure interpretations of the variation in repeat abundance between species were biologically meaningful.

As in most plants analyzed (Galindo‐González et al., 2017), the most abundant repeats in *S. viridis* were LTR retroelements belonging to the Ty3/gypsy (9.03% GP) and Ty1/copia (4.01% GP) superfamilies (Table 1). The c. 2.25:1 ratio of Ty3/gypsy to Ty1/copia‐like elements (RepeatExplorer2 data) is similar to that reported for LTR retroelements in *S. viridis* ‘ME034V’ (Thielen et al., 2020) and in *S. italica* (Zhang et al., 2012). Our analysis also detected DNA transposons (3.39% GP) and three satellite repeats, which together comprised a GP of 3.86%.

Linear modelling of repeats identified by RepeatExplorer2 showed that only Ty1/copia elements had a significant positive relationship between repeat GP and modal sequence similarity score (Figure S3b, Table S2f). In contrast, while a few RepeatExplorer‐identified Ty3/gypsy repeats did have high modal sequence similarity scores (i.e., >98% in CRM and Tekay elements), they comprised only a small proportion of the genome (Figure S3), and no relationship between GP and the modal sequence similarity score was observed. Among DANTE‐identified LTR retroelements, Ty3/gypsy CRM and Tekay elements did have high sequence identity between their two LTR repeats (Figure 2a). High sequence similarity scores likely reflect repeats that have recently amplified and hence have not had time to mutate and degrade in their sequence identities.

Linear modeling showing that the GP of Ty3/gypsy was positively associated with GC content (Figure S3a) suggests that the most amplified Ty3/gypsy elements are the youngest elements. This is consistent with the hypothesis that methylated cytosine deaminates to thymine at higher rates than other nucleotide transition/conversion events, leading to a decrease in repeat GC content over time once they have been methylated by the RdDM pathway (Kovařík et al., 1997).

### 4.2 Repeats in poly‐A and ribo‐depleted libraries

In comparing the expression of repeats identified by RepeatExplorer2 and DANTE, we showed that the TP of DANTE‐identified complete LTR retroelements in ribo‐depleted libraries was lower than observed for repeats identified using RepeatExplorer2. This is probably because RepeatExplorer2 repeat clusters are likely to include reads from a wider range of related repeats and from both complete and incomplete elements. However, in all but the leaf ribo‐depleted library, the proportion of both complete and incomplete DANTE‐identified LTR retroelements was higher than RepeatExplorer‐identified LTR retroelements (Table 2). This is likely to be because RepeatExplorer2 is not detecting transcriptionally active repeats in low copy numbers (<∼200 copies in the genome), especially low copy intronic repeats. Indeed, on analyzing the chromosomal distribution of the top 210 most transcribed complete LTR retroelements identified by DANTE, we showed that most were intronic (i.e., 142 of the top 210 repeats, Figure S7a) and are thus likely to have been transcribed with the genes and hence are in the poly‐A RNA polymerase II transcripts. TEs are known to generate new introns in many species spanning a large number of eukaryotic lineages (Gozashti et al., 2022). Gualandi et al. (2022) suggested that studies of repeats should distinguish between intronic and non‐intronic repeats because they occupy such distinctive genomic domains. However, TEs in intronic regions are likely to have arisen as a trade‐off between silencing TEs and safeguarding gene functionality (Saze, 2018). The ability to analyze the physical location of complete and incomplete LTR retroelements in relation to other genomic features (e.g., nearest genes, type of introns, gene function, and chromatin landscape) in high‐quality, annotated whole‐genome assemblies opens up exciting opportunities to gain a greater understanding of repeat transcription dynamics.

### 4.3 Hypothesis 1—A relationship between repeat TP and GP is partially supported

Since it has been proposed that abundant repeats in the genome are the most readily recognized for RNA silencing by RdDM (Lisch, 2013; Mascagni et al., 2020), we hypothesized that a repeat's TP would correlate with their GP in ribo‐depleted libraries. We did indeed observe this relationship in ribo‐depleted libraries for many repeat superfamilies identified using RepeatExplorer2 (e.g., Ty1/copia, Ty3/gypsy, and DNA transposons, Figure 4). However, some Ty1/copia elements had a higher TP in the ribo‐depleted libraries than Ty3/gypsy elements (e.g., in crown and stem tissues, Figure 6c,d, see also below), despite comprising less than half the abundance of Ty3/gypsy elements in the genome (i.e., GP of Ty3/gypsy and Ty1/copia = 9.03% and 4.01%, respectively, see Table 1).

We predicted a weaker relationship between the TP and GP of repeats in the poly‐A libraries. We reasoned that the escape of repeats from the silencing surveillance of RdDM may be independent of GP or only weakly related to it. We did find a positive relationship between GP and TP for Ty3/gypsy elements and DNA transposons identified by RepeatExplorer2 in the poly‐A libraries of leaf and inflorescence tissues (Figure 4 b,c,e,f), a relationship that was also found for Ty1/copia elements in leaf, but not inflorescence tissues (Figure 3a,d). (There were also tissue‐specific expression patterns that are discussed under hypothesis 3, below).

Of the three satellite clusters, collectively comprising nearly 4% of the genome, we observed that two of them showed very low levels of expression (ranging from 0.0010% of the transcriptome in crown tissue to 0.0086% of the transcriptome in leaf tissue; Table 3). Such low levels of expression are perhaps surprising given previous reports of the potential importance of satellite expression in genome organization and evolution. For example, it has been reported that satellite DNA transcripts can influence genome stability (Pezer et al., 2012; Plohl et al., 2008) and the pairing and segregation of chromosomes (Giraud et al., 2021). In addition, satellites may be involved in the epigenetic influence of heterochromatin and in some transcriptional responses to stress (Garrido‐Ramos, 2017; Plohl et al., 2012, 2008).

### 4.4 Hypothesis 2—Relationships between repeat TP, GC, and sequence similarity depend on repeat type

We suggested that the most transcribed repeats (i.e., those with the highest TP) would come from recently amplified, GC‐rich repeats, because GC content declines with repeat age at a rate of c. 1.03% per million years (Stritt et al., 2019). Previously, Bennetzen et al. (2012) reported evidence of a recent (few hundred thousand to 1 million years ago) burst of retroelement amplification in *S. italica*, as also shown more recently in *S. viridis* ‘ME034V’ (Thielen et al., 2020). Similarly, Zhang et al. (2012) reported that 4.7% of all LTR retroelements in *S. italica* had been active within the last 100,000 years. Such amplification results in elements with high sequence similarities between their two LTRs, as was found here for the Ty3/gypsy lineages Ogre, Tekay, and CRM and the Ty1/copia lineage Angela (Figure 2a). In *Helianthus* (sunflowers) too, transcriptionally active retroelements are the youngest elements (having the most recent insertion times), especially for Ty1/copia (Kirov et al., 2020). Such data are consistent with the results here, where we find positive correlations between modal repeat similarity and transcriptional activity (Figure 5, Figure S5). Potentially, older retroelements have mutated and degraded into “dark matter” when they become transcriptionally inactive and are not recognized by RdDM for targeting.

Further support for a relationship between retroelement age and transcriptional activity is seen in the relationship between TP and GC content across the different RNA libraries and tissues (Figure 5, Figures S4 and S5). Loss of GC content, driven by the deamination of methylated cytosine to thymine (Kovařík et al., 1997), can result in repeats losing their potential to (retro)transpose (i.e., loss of functionality).

Nevertheless, results for DNA transposons appeared to be different from those of LTR retroelements. Thus, while we did find a positive relationship between DNA transposons TP and GC in the poly‐A inflorescence transcriptomes, no other relationships between TP and GC or repeat similarity were observed for DNA transposons in the tissues and library types studied here (Tables S6 and S7). Such results highlight the importance of examining the dynamics of all types of repeats across the genome and transcriptome.

### 4.5 Hypothesis 3—Repeat expression varies with tissue type

We observed that the ribo‐depleted transcriptomes of leaf, stem, and crown tissues of *S. viridis* all had higher RepeatExplorer‐identified Ty3/gypsy and Ty1/copia repeat TPs than DNA transposon TP. Ty1/copia are relatively more transcriptionally active in poly‐A libraries than Ty3/gypsy repeats (Figure 4), which agrees with observations in other species, including *S. italica* (Yadav et al., 2015) and sunflower (*Helianthus*) species (Mascagni et al., 2020; Qiu & Ungerer, 2018). However, in the poly‐A libraries of inflorescence tissue, but not leaf tissue, the TP of DNA transposons was double that of Ty3/gypsy and Ty1/copia (Figure 6).

Similarly, LTR retroelements identified by DANTE were c. four times more abundant in the inflorescence poly‐A library than the leaf poly‐A library (Table 2), where we observed the highest repeat TP of any analysis. This increase in abundance of transcribed Ty1/copia elements in the poly‐A transcriptome greatly exceeded their 4% abundance in the genome (Table 1), suggestive of individual “selfishly” transcribing elements, perhaps taking advantage of the germ line to drive their mobility. In support of this hypothesis, Anderson et al. (2019) reported increased numbers of TE families expressed in the germ line of maize (endosperm, pollen) compared with other tissues.

### 4.6 Genome skimming and repeat annotation to study transcribed repeats

Understanding the dynamics of repeat transcription is important because of its role in retro‐element mobility and amplification, and the impact of transcribed repeats on the activity of downstream genes, and hence genome evolution (Lisch, 2013). Currently, there is no perfect tool for quantifying repeats, including TEs. This is due to their repetitive nature, and it has been suggested that approaches combining different methods, as conducted here, are beneficial (Lanciano & Cristofari, 2020).

We used RepeatExplorer2‐identified and DANTE‐identified repeats to study variations in LTR retroelement expression across different tissues and RNA library types. Both approaches gave comparable data in the directionality of changes, but the numbers were different, as both methods set different criteria for the definition of a repeat. So, while using RepeatExplorer2 gave an overview of all types of repeats, it is limited to the most abundant repeat types (>c. 200 copies), and it combines in the analysis both complete and incomplete LTR retroelements. In contrast, the identification of repeats by DANTE is limited to LTR retroelements that have been precisely defined and is not designed to identify any other type of repeat, for example, satellite repeats, DNA transposons, or indeed, previously unknown/uncharacterized types of repeats. For that, a full annotation of a well‐assembled genome, ideally at the chromosome level is needed.

Based on recent studies, the 1482 species of green plants with sequenced genomes represent only about 0.26%–0.29% of all plant species, indicating that only a small fraction has been sequenced so far (Bernal‐Gallardo & de Folter, 2024), and these vary widely in their assembly qualities. Among them, only 685 plants have genomes with well‐annotated sequences, which limits the species for which repeat expression can be studied using DANTE. In contrast, the approach with RepeatExplorer2 can be applied to any genome with DNA skimming data, irrespective of the availability of a well‐assembled and annotated genome. Applying this approach will enable an analysis of repeat expression across the 2500‐fold genome size spectrum known for vascular plants (Fernandez et al., 2024; Pellicer et al., 2018) and across the 40‐fold range of ploidy levels (i.e., 2*x* = 80*x*) encountered in angiosperms (Husband et al., 2013). We can ask if repeat transcription scales with genome size and/or repeat content, and/or GC content of the genome and if relationships are linear or curvilinear; the latter may be expected given the relationship between genome size and repeat content in genomes (Novak, Guignard et al., 2020). We can also show how repeats change with the divergence of species (e.g., Dodsworth et al., 2015; Kelly et al., 2015; Macas et al., 2015), enabling comparisons of the transcription of repeats dependent on genome size, ploidy level, repeat GC richness, and similarity, all analyzed within a phylogenetic context.

It is noted that neither RepeatExplorer2 nor DANTE are suitable methods to identify repetitive small RNAs (smRNAs), which are also found in plant transcriptomes, including in leaf and inflorescence tissue of *S. italica* (Bennetzen et al., 2012). This is because the library construction processes are not designed to isolate them. These smRNAs are an integral part of the RdDM pathway and include the degradation products of some of the longer RNA reads that will be present in the ribo‐depleted libraries analyzed here. In other variants of transcriptome studies, some approaches try to remove “noise” from their gene transcription analyses by removing reads that map to both TEs and genes (Ansaloni et al., 2019). However, in so doing, these approaches remove data on repeat transcripts that constitute an important part of the transcriptome, both in terms of understanding their role in regulating the expression of repeats and genes and hence driving evolution, as well as in assessing the metabolic and nutrient costs associated with maintaining the repeats present in the genome (Wang et al., 2021).

We present here a test case for using RepeatExplorer2 as a means to identify repeat transcription. The approach characterizes all types of repeats, including those never before encountered, and it can be applied to any species with genome skimming and transcriptomic data. It enables comparative repeat transcription analyses without the data being confounded by genome assembly and annotation quality or the variant parameters used to define a repeat. By keeping analytical settings constant in the RepeatExplorer2 analytical pipeline developed here, we will enable straightforward repeat transcription analyses across any species, including those without characterized genomes and with widely different genome sizes and/or in diploid/polyploid complexes.

## AUTHOR CONTRIBUTIONS

**Ana Luiza Franco**: Data curation; formal analysis; investigation; methodology; validation; visualization; writing—original draft. **Wenjia Gu**: Data curation; formal analysis; investigation; methodology; validation; visualization. **Petr Novak**: Data curation; investigation; methodology; software; validation. **Ilia J. Leitch**: Conceptualization; data curation; investigation; supervision; validation; writing—review and editing. **Lyderson F. Viccini**: Resources; supervision; visualization; writing—review and editing. **Andrew R. Leitch**: Conceptualization; data curation; funding acquisition; investigation; methodology; project administration; resources; supervision; validation; visualization; writing—original draft; writing—review and editing.

## CONFLICT OF INTEREST STATEMENT

The authors declare no conflicts of interest.

## Supporting information



Figure S1. Cluster graphs showing examples of repeat clusters (CL) identified in *Setaria viridis* using RepeatExplorer2.
Figure S2. Examples of repeat sequence similarity scores (%) of reads from two of the 208 repeat clusters generated by RepeatExplorer2 using genomic DNA of *Setaria viridis*.
Figure S3. Scatterplot showing the genomic characteristics of the repeat clusters.
Figure S4. Scatterplots showing the relationship between the transcriptome proportion (TP) and the genomic characteristics for repeat clusters in poly‐A libraries of inflorescence tissue.
Figure S5. Scatterplots showing the relationship between the transcriptome proportion (TP) and the genomic characteristics for repeat clusters in ribo‐depleted libraries from crown and stem tissues.
Figure S6. Pipelines used to quantify and characterize repeats in the genome and transcriptomes of *Setaria viridis*.
Figure S7. RNAseq reads mapped in genome assembly. (a) Number of full‐length and incomplete repeats annotated in the genome using DANTE and the proportion of those intronics and non‐intronics (b) Heatmap showing the repeats differentially expressed according to Deseq2 (c) IGV screenshot of CRM type up regulated in ribo‐depleted leaf (d) IGV screenshot of repeats in intronic regions

Table S1. NCBI accession numbers and sources of DNA and RNA sequence read datasets of *Setaria viridis* ‘A10’ used in this study.
Table S2. Relationships between GP, GC ratio and sequence similarities of repeat reads.
Table S3. Number of complete and incomplete elements annotated in the entire *Setaria viridis* genome by DANTE software.
Table S4. Transcript proportions (TP) of repeats in the three replicate ribo‐depleted RNA libraries prepared from leaf tissue of *Setaria viridis*.
Table S5. Transcript proportions (TP) of repeats in three‐replicate poly‐A RNA libraries prepared from leaf tissue of *Setaria viridis*.
Table S6. Linear regression models analysing the relationship between repeat (Ty1/copia, Ty3/gypsy and DNA transposon) transcriptome proportions (TP) in leaf poly‐A (intercept) and ribo‐depleted libraries and their genome proportion (GP), GC content and sequence similarities.
Table S7. Linear regression models analysing the relationship between repeat transcriptome proportions (TP) for different RNA libraries, tissues (stem, crown and inflorescence) and genomic features of Ty1/copia, Ty3/gypsy and DNA transposon repeats.
Table S8. Transcript proportions (TPs) of repeats in the three replicate inflorescence poly‐A RNA libraries of *Setaria viridis*.
Table S9. Transcript proportions (TPs) of repeats in the three replicate ribo‐depleted RNA libraries prepared from crown tissue of *Setaria viridis*.
Table S10. Transcript proportions (TPs) of repeats in the three replicate ribo‐depleted RNA libraries prepared from stem tissue of *Setaria viridis*.

## Data Availability

All raw sequencing files generated in this study are available on NCBI Sequence Read Archive (SRA) under BioProject accession number PRJNA1020582. The dataset supporting this paper is accessible in the Dryad Repository at https://doi.org/10.5061/dryad.rjdfn2znh.
