# Supplementary material for: Contrasting distributions and expression characteristics of transcribing repeats in Setaria viridis
Source: Plant Genome. 2025 Jan 9;18(1):e20551. doi: 10.1002/tpg2.20551 (PMC11718148; doi:10.1002/tpg2.20551)
Supplement: Supplementary file 1 [file TPG2-18-e20551-s001.docx]

[**Supplemental Tables**	2](#_Toc163168184)

[**Table S1.** NCBI accession numbers and sources of DNA and RNA sequence read datasets of *Setaria viridis* ‘A10’ used in this study.	2](#_Toc163168185)

[**Table S2.** Relationships between GP, GC ratio and sequence similarities of repeat reads.	4](#_Toc163168186)

[**Table S3**. Number of complete and incomplete elements annotated in the entire *Setaria viridis* genome by DANTE software.	6](#_Toc163168187)

[**Table S4**. Transcript proportions (TP) of repeats in the three replicate ribo-depleted RNA libraries prepared from leaf tissue of *Setaria viridis*.	7](#_Toc163168188)

[**Table S5**. Transcript proportions (TP) of repeats in three-replicate poly-A RNA libraries prepared from leaf tissue of *Setaria viridis*.	9](#_Toc163168189)

[**Table S6**. Linear regression models analysing the relationship between repeat (Ty1/copia, Ty3/gypsy and DNA transposon) transcriptome proportions (TP) in leaf poly-A (intercept) and ribo-depleted libraries and their genome proportion (GP), GC content and sequence similarities.	11](#_Toc163168190)

[**Table S7**. Linear regression models analysing the relationship between repeat transcriptome proportions (TP) for different RNA libraries, tissues (stem, crown and inflorescence) and genomic features of Ty1/copia, Ty3/gypsy and DNA transposon repeats.	13](#_Toc163168191)

[**Table S8**. Transcript proportions (TPs) of repeats in the three replicate inflorescence poly-A RNA libraries of *Setaria viridis*.	16](#_Toc163168192)

**[Table S9](#_Toc163168193)**[. Transcript proportions (TPs) of repeats in the three replicate ribo-depleted RNA libraries prepared from crown tissue of](#_Toc163168193) *[Setaria viridis](#_Toc163168193)*[.	18](#_Toc163168193)

**[Table S10](#_Toc163168194)**[. Transcript proportions (TPs) of repeats in the three replicate ribo-depleted RNA libraries prepared from stem tissue of](#_Toc163168194) *[Setaria viridis](#_Toc163168194)*[.	20](#_Toc163168194)

# **Supplemental Tables**

# **Table S1. NCBI accession numbers and sources of DNA and RNA sequence read datasets of *Setaria viridis* ‘A10’ used in this study.**

**The original sources of the data are given in the column ‘Reference’. Descriptions of the library (taken from the source papers), the library layout (single- or paired-end reads), the library type (genomic, ribo-depleted or poly-A), the tissue source of the library and the numbers of reads analysed are shown.**

| **Accession number in NCBI** | **Description** | **Library layout** | **Library Type** | **Tissue** | **Number of reads after filtering** | **Reference** |
| --- | --- | --- | --- | --- | --- | --- |
| SRR10051273 | TB_setaria_16_0782 | Paired-end | Genomic DNA | Leaf | 74,498,042 | Mamidi et al. 2020^1^ |
| SRR26158246 | wild-type rep1 | paired-end | Ribo-depleted | Leaf | 183,624,756 | This study |
| SRR26158245 | wild-type rep2 | paired-end | Ribo-depleted | Leaf | 115,123,222 | This study |
| SRR26158244 | wild-type rep3 | paired-end | Ribo-depleted | Leaf | 184,007,030 | This study |
| SRR26158243 | wild-type rep1 | paired-end | Poly-A | Leaf | 151,136,544 | This study |
| SRR26158242 | wild-type rep2 | paired-end | Poly-A | Leaf | 164,751,814 | This study |
| SRR26158241 | wild-type rep3 | paired-end | Poly-A | Leaf | 194,996,794 | This study |
| SRR3176660 | 9 DAS rep1 watered | paired-end | Ribo-depleted | Upper region (stem) | 34,113,920 | Sebastian et al. 2016^2^ |
| SRR3176668 | 9 DAS rep2 watered | paired-end | Ribo-depleted | Upper region (stem) | 20,053,518 | Sebastian et al. 2016^2^ |
| SRR3176676 | 9 DAS rep3 watered | paired-end | Ribo-depleted | Upper region (stem) | 30,755,030 | Sebastian et al. 2016^2^ |
| SRR3176661 | 9 DAS rep1 watered | paired-end | Ribo-depleted | Lower region (crown) | 22,280,606 | Sebastian et al. 2016^2^ |
| SRR3176669 | 9 DAS rep2 watered | paired-end | Ribo-depleted | Lower region (crown) | 14,717,318 | Sebastian et al. 2016^2^ |
| SRR3176677 | 9 DAS rep3 watered | paired-end | Ribo-depleted | Lower region (crown) | 21,292,550 | Sebastian et al. 2016^2^ |
| SRR5750472 | wild-type rep1 | single-end | Poly-A | Inflorescence primordia | 11,376,412 | Yang et al. 2018^3^ |
| SRR5750473 | wild-type rep2 | single-end | Poly-A | Inflorescence primordia | 11,519,902 | Yang et al. 2018^3^ |
| SRR5750474 | wild-type rep3 | single-end | Poly-A | Inflorescence primordia | 11,963,907 | Yang et al. 2018^3^ |

**^1^Mamidi, S., Healey, A., Huang, P., et al.** (2020) A genome resource for green millet *Setaria viridis* enables discovery of agronomically valuable loci. *Nat. Biotechnol.*, **38**, 1203–1210.

**^2^Sebastian, J., Yee, M., Goudinho, W., et al.** (2016) Grasses suppress shoot-borne roots to conserve water during drought. *Proc. Natl. Acad. Sci.*, **113**, 8861–8866.

**^3^Yang, J., Thames, S., Best, N.B., Jiang, H., Huang, P., Dilkes, B.P. and Eveland, A.L.** (2018) Brassinosteroids modulate meristem fate and differentiation of unique inflorescence morphology in *Setaria viridis*. *Plant Cell*, **30**, 48–66

# **Table S2. Relationships between GP, GC ratio and sequence similarities of repeat reads.**

**The baseline level (intercept) is the Ty3/gypsy element in each case. Std Error = standard error; df= degrees of freedom and adjusted R² of the models are reported.**

| ***a****) Log GP ~ GC content (DNA transposons)* |  |  |  |  |
| --- | --- | --- | --- | --- |
|  | Estimate | Std. Error | t value | Pr(>\|t\|) |
| (Intercept) | -2.60307 | 0.97929 | -2.658 | 0.0122 * |
| GC content | 0.03153 | 0.02417 | 1.304 | 0.2014 |
| Residual SE= 0.4993 on 32 on df, R²adjusted 0.0208 | |  |  |  |
|  |  |  |  |  |
| ***b)*** *Log GP ~ GC content (Ty3/gypsy)* |  |  |  |  |
|  | Estimate | Std. Error | t value | Pr(>\|t\|) |
| (Intercept) | -2.68956 | 0.59556 | -4.516 | 6.22e-05 *** |
| GC content | 0.0377 | 0.01332 | 2.831 | 0.00746 ** |
| Residual SE= 0.6002 on 37 df, adjusted R²= 0.1558 | |  |  |  |
|  |  |  |  |  |
| ***c)*** *Log GP ~ GC content (Ty1/copia )* |  |  |  |  |
|  | Estimate | Std. Error | t value | Pr(>\|t\|) |
| (Intercept) | -1.66968 | 0.79983 | -2.088 | 0.0486 * |
| GC content | 0.0118 | 0.01795 | 0.657 | 0.5178 |
| Residual SE= 0.6271 on 22 df, adjusted R²= -0.02532 | |  |  |  |
|  |  |  |  |  |
| **d)** *Log GP~ Similarity (DNA transposons)* |  |  |  |  |
|  | Estimate | Std. Error | t value | Pr(>\|t\|) |
| (Intercept) | 2.04369 | 3.24786 | 0.629 | 0.534 |
| Similarity | -0.03561 | 0.03426 | -1.039 | 0.306 |
| Residual SE= 0.504 on 32 df, adjusted R²= 0.002424 | |  |  |  |
|  |  |  |  |  |
| ***e)*** *Log GP ~ Similarity (Ty3/gypsy)* |  |  |  |  |
|  | Estimate | Std. Error | t value | Pr(>\|t\|) |
| (Intercept) | -3.75871 | 3.00232 | -1.252 | 0.218 |
| Similarity | 0.02988 | 0.03281 | 0.911 | 0.368 |
| Residual SE= 0.6548 on 37 df, adjusted R²= -0.004502 | |  |  |  |
|  |  |  |  |  |
| ***f)*** *Log GP ~ Similarity (Ty1/copia)* |  |  |  |  |
|  | Estimate | Std. Error | t value | Pr(>\|t\|) |
| (Intercept) | -13.79437 | 3.02696 | -4.557 | 0.000155 *** |
| Similarity | 0.13448 | 0.03218 | 4.179 | 0.000390 *** |
| Residual SE= 0.4728 on 22 df, adjusted R²= 0.4172 | |  |  |  |
|  |  |  |  |  |
| **g)** *GC content~ Similarity*Repeat Type* |  |  |  |  |
|  | Estimate | Std. Error | t value | Pr(>\|t\|) |
| (Intercept - Copia) | -7.122 | 46.6431 | -0.153 | 0.879 |
| Similarity | 0.5437 | 0.4659 | 1.096 | 0.277 |
| Repeat Type Gypsy | 16.5194 | 57.3704 | 0.288 | 0.774 |
| Similarity:Repeat Type Gypsy | 0.001639 | 0.6157 | -0.266 | 0.791 |
| Residual SE= 7.285 on 59 df, adjusted R²= -0.01158 | |  |  |  |
|  | |  |  |  |

# **Table S3. Number of complete and incomplete elements annotated in the entire *Setaria viridis* genome by DANTE software**

| **Lineage** | **LTR  mean length  [bp]** | **Element mean length  [bp]** | **Incomplete LTR retroelements** | | | | **Complete LTR retroelements** |
| --- | --- | --- | --- | --- | --- | --- | --- |
|  |  |  | Number with no LTR detected | Number with with just LTR | Number with just LTR & PBS | Number with just LTR & TSD | Number with LTR, PBS, TSD |
| Class_I\|LTR\|Ty1/copia\|Ale | 188 | 5053 | 500 | 130 | 136 | 322 | 81 |
| Class_I\|LTR\|Ty1/copia\|Alesia | 336 | 4889 | 6 | 0 | 2 | 0 | 0 |
| Class_I\|LTR\|Ty1/copia\|Angela | 1408 | 8080 | 723 | 136 | 697 | 1026 | 486 |
| Class_I\|LTR\|Ty1/copia\|Bianca | 232 | 6238 | 43 | 8 | 9 | 14 | 6 |
| Class_I\|LTR\|Ty1/copia\|Ikeros | 448 | 6957 | 77 | 23 | 67 | 128 | 51 |
| Class_I\|LTR\|Ty1/copia\|Ivana | 347 | 5466 | 122 | 20 | 64 | 122 | 48 |
| Class_I\|LTR\|Ty1/copia\|SIRE | 1415 | 10846 | 153 | 33 | 58 | 80 | 33 |
| Class_I\|LTR\|Ty1/copia\|TAR | 741 | 6201 | 126 | 37 | 58 | 88 | 31 |
| Class_I\|LTR\|Ty1/copia\|Tork | 471 | 5166 | 66 | 9 | 35 | 60 | 29 |
| Class_I\|LTR\|Ty3/gypsy\| chromovirus\|CRM | 667 | 7070 | 265 | 77 | 175 | 82 | 31 |
| Class_I\|LTR\|Ty3/gypsy\| chromovirus\|Reina | 318 | 5621 | 157 | 26 | 68 | 116 | 45 |
| Class_I\|LTR\|Ty3/gypsy\| chromovirus\|Tekay | 2468 | 12554 | 797 | 295 | 589 | 712 | 323 |
| Class_I\|LTR\|Ty3/gypsy\| non-chromovirus\|OTA\|Athila | 989 | 10024 | 259 | 8 | 4 | 10 | 0 |
| Class_I\|LTR\|Ty3/gypsy\|non-chromovirus\|OTA\|Tat\|Ogre | 1117 | 13404 | 678 | 222 | 86 | 666 | 17 |
| Class_I\|LTR\|Ty3/gypsy\|non-chromovirus\|OTA\|Tat\|Retand | 699 | 13062 | 2142 | 254 | 435 | 682 | 297 |

LTR = long terminal repeats; PBS = primer binding site, TSD = target site duplication, bp = base pairs.

# **Table S4. Transcript proportions (TP) of repeats in the three replicate ribo-depleted RNA libraries prepared from leaf tissue of *Setaria viridis*.**

**The material is the same as in Table S5, differing only in the type of library prepared.**

|  |  | **Leaf ribo-depleted** | | | | | |  |
| --- | --- | --- | --- | --- | --- | --- | --- | --- |
|  |  | Replicate 1 |  | Replicate 2 |  | Replicate 3 |  |  |
|  | **Total Reads** | 183,624,756 |  | 115,123,222 |  | 184,007,030 |  |  |
|  | **Total Organelles+ rDNA+ tRNA** | 167,246,165 |  | 107,420,926 |  | 171,470,160 |  |  |
| **Repeat Class/Superfamily** | **Total minus (Organelles+ rDNA  + tRNA)** | 16,378,591 |  | 7,702,296 |  | 12,536,870 |  |  |
| **Class I –**  **RNA retroelements** | **Repeat Lineage** | **Reads mapped** | **TP %** | **Reads mapped** | **TP %** | **Reads mapped** | **TP %** | **Mean TP %** |
| ***Ty3/gypsy*** |  | **11763** | **0.072** | **5866** | **0.076** | **11102** | **0.089** | **0.079** |
|  | *Athila* | 749 | 0.005 | 446 | 0.006 | 688 | 0.005 | 0.005 |
|  | *CRM* | 660 | 0.004 | 435 | 0.006 | 845 | 0.007 | 0.005 |
|  | *Ogre* | 1161 | 0.007 | 678 | 0.009 | 1323 | 0.011 | 0.009 |
|  | *Reina* | 46 | 0.000 | 21 | 0.000 | 35 | 0.000 | 0.000 |
|  | *Retand* | 5755 | 0.035 | 2195 | 0.028 | 4475 | 0.036 | 0.033 |
|  | *Tekay* | 3392 | 0.021 | 2091 | 0.027 | 3736 | 0.030 | 0.026 |
| ***Ty1/copia*** |  | **7325** | **0.045** | **3710** | **0.048** | **5813** | **0.046** | **0.046** |
|  | *Ale* | 1618 | 0.010 | 652 | 0.008 | 899 | 0.007 | 0.009 |
|  | *Angela* | 1409 | 0.009 | 801 | 0.010 | 1546 | 0.012 | 0.010 |
|  | *Bianca* | 13 | 0.000 | 30 | 0.000 | 31 | 0.000 | 0.000 |
|  | *Ikeros* | 236 | 0.001 | 112 | 0.001 | 194 | 0.002 | 0.001 |
|  | *Ivana* | 1662 | 0.010 | 777 | 0.010 | 1326 | 0.011 | 0.010 |
|  | *SIRE* | 1651 | 0.010 | 924 | 0.012 | 1114 | 0.009 | 0.010 |
|  | *TAR* | 322 | 0.002 | 196 | 0.003 | 274 | 0.002 | 0.002 |
|  | *Tork* | 414 | 0.003 | 218 | 0.003 | 429 | 0.003 | 0.003 |
| ***LINE*** |  | **1051** | **0.006** | **410** | **0.005** | **459** | **0.004** | **0.005** |
| ***Unclassified LTR*** |  | **11547** | **0.071** | **5349** | **0.069** | **9709** | **0.077** | **0.072** |
| ***Pararetrovirus*** |  | **7** | **0.000** | **2** | **0.000** | **9** | **0.000** | **0.000** |
| **Class II - DNA transposons** |  | **4012** | **0.024** | **2352** | **0.031** | **3693** | **0.029** | **0.028** |
|  | *CACTA* | 1480 | 0.009 | 964 | 0.013 | 1519 | 0.012 | 0.011 |
|  | *Helitron* | 11 | 0.000 | 30 | 0.000 | 106 | 0.001 | 0.000 |
|  | *Mariner* | 19 | 0.000 | 9 | 0.000 | 14 | 0.000 | 0.000 |
|  | *Mutator* | 1651 | 0.010 | 802 | 0.010 | 1307 | 0.010 | 0.010 |
|  | *Harbinger* | 507 | 0.003 | 341 | 0.004 | 423 | 0.003 | 0.004 |
|  | *HAT* | 344 | 0.002 | 206 | 0.003 | 324 | 0.003 | 0.002 |
| **Satellite repeats** |  | **960** | **0.006** | **652** | **0.008** | **1423** | **0.011** | **0.009** |
| **Unknown repeats** |  | **10696** | **0.065** | **5742** | **0.075** | **8785** | **0.070** | **0.070** |
| **Total** |  | **47,361** | **0.2892** | **24,083** | **0.31267** | **40,993** | **0.32698** | **0.310** |

# **Table S5. Transcript proportions (TP) of repeats in three-replicate poly-A RNA libraries prepared from leaf tissue of *Setaria viridis*.**

**The material is the same as in Table S4, differing only in the type of library prepared.**

|  |  |  |  | **Leaf poly-A** |  |  |  |  |
| --- | --- | --- | --- | --- | --- | --- | --- | --- |
|  |  | Replicate 1 |  | Replicate 2 |  | Replicate 3 |  |  |
|  | **Total Reads** | 151,136,544 |  | 164,751,814 |  | 194,996,794 |  |  |
|  | **Total Organelles + rDNA + tRNA** | 5,932,140 |  | 9,677,962 |  | 8,277,431 |  |  |
| **Repeat Class/Superfamily** | **Total minus (Organelles  + rDNA+ tRNA)** | 145,204,404 |  | 155,073,852 |  | 186,719,363 |  |  |
| **Class I – RNA retroelements** | **Repeat Lineage** | **Reads mapped** | **TP %** | **Reads mapped** | **TP %** | **Reads mapped** | **TP %** | **Mean TP %** |
| ***Ty3/gypsy*** |  | **6419** | **0.004** | **6278** | **0.004** | **9855** | **0.005** | **0.005** |
|  | *Athila* | 1205 | 0.001 | 990 | 0.001 | 1700 | 0.001 | 0.001 |
|  | *CRM* | 674 | 0.000 | 758 | 0.000 | 1214 | 0.001 | 0.001 |
|  | *Ogre* | 783 | 0.001 | 593 | 0.000 | 1087 | 0.001 | 0.001 |
|  | *Reina* | 49 | 0.000 | 64 | 0.000 | 85 | 0.000 | 0.000 |
|  | *Retand* | 1481 | 0.001 | 1704 | 0.001 | 2715 | 0.001 | 0.001 |
|  | *Tekay* | 2227 | 0.002 | 2169 | 0.001 | 3054 | 0.002 | 0.002 |
| ***Ty1/copia*** |  | **13778** | **0.009** | **12086** | **0.008** | **14214** | **0.008** | **0.008** |
|  | *Ale* | 2368 | 0.002 | 2308 | 0.001 | 2527 | 0.001 | 0.001 |
|  | *Angela* | 1259 | 0.001 | 984 | 0.001 | 1355 | 0.001 | 0.001 |
|  | *Bianca* | 2 | 0.000 | 21 | 0.000 | 37 | 0.000 | 0.000 |
|  | *Ikeros* | 1003 | 0.001 | 320 | 0.000 | 1044 | 0.001 | 0.000 |
|  | *Ivana* | 2768 | 0.002 | 2511 | 0.002 | 3424 | 0.002 | 0.002 |
|  | *SIRE* | 5433 | 0.004 | 5341 | 0.003 | 4756 | 0.003 | 0.003 |
|  | *TAR* | 576 | 0.000 | 443 | 0.000 | 839 | 0.000 | 0.000 |
|  | *Tork* | 369 | 0.000 | 158 | 0.000 | 232 | 0.000 | 0.000 |
| ***LINE*** |  | **3760** | **0.003** | **3134** | **0.002** | **3844** | **0.002** | **0.002** |
| ***Unclassified LTR*** |  | **13867** | **0.010** | **13257** | **0.009** | **21529** | **0.012** | **0.010** |
| ***Pararetrovirus*** |  | **5** | **0.000** | **0** | **0.000** | **14** | **0.000** | **0.000** |
| **Class II - DNA transposons** |  | **12994** | **0.009** | **10424** | **0.007** | **17967** | **0.010** | **0.008** |
|  | *CACTA* | 2534 | 0.002 | 2132 | 0.001 | 3919 | 0.002 | 0.002 |
|  | *Helitron* | 27 | 0.000 | 63 | 0.000 | 407 | 0.000 | 0.000 |
|  | *Mariner* | 60 | 0.000 | 53 | 0.000 | 97 | 0.000 | 0.000 |
|  | *Mutator* | 6901 | 0.005 | 4804 | 0.003 | 8574 | 0.005 | 0.004 |
|  | *Harbinger* | 2507 | 0.002 | 2809 | 0.002 | 3842 | 0.002 | 0.002 |
|  | *HAT* | 965 | 0.001 | 563 | 0.000 | 1128 | 0.001 | 0.001 |
| **Satellite repeats** |  | **15** | **0.000** | **23** | **0.000** | **26** | **0.000** | **0.000** |
| **Unknown repeats** |  | **56835** | **0.039** | **56859** | **0.037** | **74972** | **0.040** | **0.039** |
| **Total** |  | **107,673** | **0.0742** | **102,061** | **0.066** | **142,421** | **0.076** | **0.0721** |

# **Table S6. Linear regression models analysing the relationship between repeat (Ty1/copia, Ty3/gypsy and DNA transposon) transcriptome proportions (TP) in leaf poly-A (intercept) and ribo-depleted libraries and their genome proportion (GP), GC content and modal sequence similarities.**

| **a)** **Leaf** |  |  |  |  |
| --- | --- | --- | --- | --- |
| *Log TP ~ Log GP + Library type (Ty1/copia)* | |  |  |  |
|  | Estimate | Std. Error | t value | Pr(>\|t\|) |
| (Intercept) | -3.2526 | 0.1597 | -20.371 | < 2e-16 *** |
| Log GP | 0.8128 | 0.1198 | 6.783 | 4.29e-10 *** |
| Library Ribo-depleted | 0.9688 | 0.1398 | 6.932 | 2.01e-10 *** |
| Residual SE = 0.7853 on 124 df, adjusted R² = 0.4101 | | |  |  |
|  |  |  |  |  |
| **b)** **Leaf** |  |  |  |  |
| *Log TP ~ Log GP + Library type (Ty3/gypsy)* | |  |  |  |
|  | Estimate | Std. Error | t value | Pr(>\|t\|) |
| (Intercept) | -3.41208 | 0.06756 | -50.51 | <2e-16 *** |
| Log GP | 0.9739 | 0.05292 | 18.4 | <2e-16 *** |
| Library Ribo-depleted | 1.17896 | 0.06479 | 18.2 | <2e-16 *** |
| Residual SE = 0.4778 on 216 df, adjusted R² = 0.7421 | | |  |  |
|  |  |  |  |  |
| **c)** **Leaf** |  |  |  |  |
| *Log TP ~ Log GP + Library type (Class II DNA Transposons)* | | |  |  |
|  | Estimate | Std. Error | t value | Pr(>\|t\|) |
| (Intercept) | -3.02577 | 0.11417 | -26.502 | <2e-16 *** |
| Log GP | 0.79922 | 0.0769 | 10.394 | <2e-16 *** |
| Library Ribo-depleted | 0.69077 | 0.07491 | 9.221 | <2e-16 *** |
| Residual SE = 0.5344 on 193 df, adjusted R² = 0.4945 | | |  |  |
|  |  |  |  |  |
| **d)** **Leaf** |  |  |  |  |
| *Log TP ~ Similarity + GC ratio + Library type (Ty1/copia)* | | |  |  |
|  | Estimate | Std. Error | t value | Pr(>\|t\|) |
| (Intercept) | -15.5437 | 2.044506 | -7.603 | 6.43e-12 *** |
| Similarity | 0.092752 | 0.021843 | 4.246 | 4.25e-05 *** |
| GC ratio | 0.059425 | 0.009652 | 6.156 | 9.66e-09 *** |
| Library Ribo-depleted | 1.00714 | 0.132933 | 7.576 | 7.38e-12 *** |
| Residual SE = 0.7455 on 123 df, adjusted R² = 0.4684 | | |  |  |
|  |  |  |  |  |
| **e)** **Leaf** |  |  |  |  |
| *Log TP ~ Similarity + GC ratio + Library type (Ty3/gypsy)* | | |  |  |
|  | Estimate | Std. Error | t value | Pr(>\|t\|) |
| (Intercept) | -9.71802 | 1.33954 | -7.255 | 7.20e-12 *** |
| Similarity | 0.03794 | 0.01483 | 2.559 | 0.0112 * |
| GC ratio | 0.0439 | 0.00629 | 6.98 | 3.62e-11 *** |
| Library Ribo-depleted | 1.10705 | 0.09144 | 12.107 | < 2e-16 *** |
| Residual SE = 0.6755 on 215 df, adjusted R² = 0.4845 | | |  |  |
|  |  |  |  |  |
| **f)** **Leaf** |  |  |  |  |
| *Log TP ~ Similarity + GC ratio + Library type (Class II DNA transposons)* | | | |  |
|  | Estimate | Std. Error | t value | Pr(>\|t\|) |
| (Intercept) | -1.625 | 1.784305 | -0.911 | 0.364 |
| Similarity | -0.029468 | 0.018165 | -1.622 | 0.106 |
| GC ratio | 0.008457 | 0.012903 | 0.655 | 0.513 |
| Library Ribo-depleted | 0.689092 | 0.093078 | 7.403 | 4.06e-12 *** |
| Residual SE = 0.6515 on 192 df, adjusted R² = 0.2197 | | |  |  |
| Signif. codes: 0 ‘***’ 0.001 ‘**’ 0.01 ‘*’ 0.05 ‘.’ 0.1 ‘ ’ 1 | |  |  |  |

# **Table S7. Linear regression models analysing the relationship between repeat transcriptome proportions (TP) for different RNA libraries (ribo-depleted and poly-A), tissues (stem, crown and inflorescence) and genomic features (i.e. genome proportions, GP), GC content and modal sequence similarities for Ty1/copia, Ty3/gypsy and DNA transposon repeats identified using RepeatExplorer2.**

| **a) Inflorescence poly-A RNA:** *Log TP~ Log GP (Ty1/copia)* |  |  |  |  |
| --- | --- | --- | --- | --- |
|  | Estimate | Std. Error | t value | Pr(>\|t\|) |
| (Intercept) | -3.4123 | 0.2186 | -15.606 | <2e-16 *** |
| Log GP | 0.1478 | 0.2028 | 0.729 | 0.469 |
| Residual SE = 0.8135 on 49 df, adjusted R² = -0.009456 | | | |  |
|  |  |  |  |  |
| **b) Inflorescence poly-A RNA:** *Log TP~ Log GP (Ty3/gypsy)* |  |  |  |  |
|  | Estimate | Std. Error | t value | Pr(>\|t\|) |
| (Intercept) | -3.35971 | 0.09814 | -34.233 | < 2e-16 *** |
| Log GP | 0.68186 | 0.09397 | 7.256 | 1.03e-10 *** |
| Residual SE = 0.5387 on 96 df, adjusted R² = 0.3475 |  |  |  |  |
|  |  |  |  |  |
| **c) Inflorescence poly-A RNA:** *Log TP~ Log GP (DNA transposons)* |  |  |  |  |
|  | Estimate | Std. Error | t value | Pr(>\|t\|) |
| (Intercept) | -2.5673 | 0.1867 | -13.754 | < 2e-16 *** |
| Log GP | 0.845 | 0.1344 | 6.286 | 1.22e-08 *** |
| Residual SE = 0.625 on 88 df, adjusted R² = 0.302 | | |  |  |
|  |  |  |  |  |
| **d)** **Inflorescence poly-A RNA**: *Log TP~ Similarity + GC content (Ty1/copia)* |  |  |  |  |
|  | Estimate | Std. Error | t value | Pr(>\|t\|) |
| (Intercept) | -1.35152 | 3.53222 | -0.383 | 0.70369 |
| Similarity | -0.04597 | 0.03527 | -1.303 | 0.19866 |
| GC content | 0.04673 | 0.01696 | 2.754 | 0.00828 ** |
| Residual SE = 0.7501 on 48 df, adjusted R² = 0.1417 | | |  |  |
|  |  |  |  |  |
| **e)** **Inflorescence poly-A RNA**: *Log TP~ Similarity + GC content (Ty3/gypsy )* |  |  |  |  |
|  | Estimate | Std. Error | t value | Pr(>\|t\|) |
| (Intercept) | -6.20625 | 2.13271 | -2.91 | 0.00450 ** |
| Similarity | 0.01291 | 0.02418 | 0.534 | 0.59477 |
| GC content | 0.02436 | 0.88793 | 2.744 | 0.00727 ** |
| Residual SE = 0.6431 on 95 df, adjusted R² = 0.0701 | | |  |  |
|  |  |  |  |  |
| **f)** **Inflorescence poly-A RNA**: *Log TP~ Similarity + GC content  (DNA transposons)* |  |  |  |  |
|  | Estimate | Std. Error | t value | Pr(>\|t\|) |
| (Intercept) | -4.42139 | 2.88899 | -1.53 | 0.12934 |
| Similarity | -0.0163 | 0.02967 | -0.549 | 0.58402 |
| GC content | 0.05641 | 0.02082 | 2.71 | 0.00802 ** |
| Residual SE = 0.734 on 92 df, adjusted R² = 0.05529 | | |  |  |
|  |  |  |  |  |
| **g)** **Stem/Crown ribo-depleted:** *Log TP ~ Log GP + Tissue (Ty1/copia )* | |  |  |  |
|  | Estimate | Std. Error | t value | Pr(>\|t\|) |
| (Intercept) | -2.28065 | 0.15017 | -15.188 | < 2e-16 *** |
| Log GP | 0.9083 | 0.11366 | 7.992 | 8.52e-13 *** |
| Tissue Stem | 0.04487 | 0.13085 | 0.343 | 0.732 |
| Residual SE = 0.7314 on 122 df, adjusted R² = 0.3331 | | |  |  |
|  |  |  |  |  |
| **h)** **Stem/Crown ribo-depleted:** *Log TP ~ Log GP + Tissue (Ty3/gypsy )* | |  |  |  |
|  | Estimate | Std. Error | t value | Pr(>\|t\|) |
| (Intercept) | -2.61366 | 0.06617 | -39.502 | <2e-16 *** |
| Log GP | 0.92561 | 0.0505 | 18.329 | <2e-16 *** |
| Tissue Stem | 0.06299 | 0.0625 | 1.008 | 0.315 |
| Residual SE = 0.4667 on 220 df, adjusted R² = 0.6012 | | |  |  |
|  |  |  |  |  |
| **i)** **Stem/Crown ribo-depleted:** *Log TP ~ Log GP + Tissue (Class II DNA transposons )* | |  |  |  |
|  | Estimate | Std. Error | t value | Pr(>\|t\|) |
| (Intercept) | -2.50427 | 0.10983 | -22.801 | <2e-16 *** |
| Log GP | 0.85724 | 0.0739 | 11.601 | <2e-16 *** |
| Tissue Stem | 0.08104 | 0.07211 | 1.124 | 0.262 |
| Residual SE = 0.5099 on 197 df, adjusted R² = 0.4016 | | |  |  |
|  |  |  |  |  |
| **j) Stem/Crown ribo-depleted:** *Log TP ~ Similarity + GC content + Tissue (Ty1/copia)* | |  |  |  |
|  | | |  |  |
|  | Estimate | Std. Error | t value | Pr(>\|t\|) |
| (Intercept) | -16.795181 | 1.970463 | -8.523 | 5.14e-14 *** |
| Similarity | 0.121201 | 0.021111 | 5.741 | 7.12e-08 *** |
| GC content | 0.04771 | 0.009213 | 5.179 | 9.02e-07 *** |
| Tissue Stem | 0.037788 | 0.128718 | 0.294 | 0.77 |
| Residual SE = 0.7195 on 121 df, adjusted R² = 0.3545 | | |  |  |
|  |  |  |  |  |
| **k) Stem/Crown ribo-depleted:** *Log TP ~ Similarity + GC content + Tissue (Ty3/gypsy)* | |  |  |  |
|  | | |  |  |
|  | Estimate | Std. Error | t value | Pr(>\|t\|) |
| (Intercept) | -4.907623 | 1.407549 | -3.487 | 0.000591 *** |
| Similarity | 0.001531 | 0.015635 | 0.098 | 0.922099 |
| GC content | 0.028515 | 0.006634 | 4.298 | 2.59e-05 *** |
| Tissue Stem | 0.047935 | 0.095483 | 0.502 | 0.61615 |
| Residual SE = 0.7128 on 219 df, adjusted R² = 0.06951 | | | |  |
|  |  |  |  |  |
| **l) Stem/Crown ribo-depleted:**  *Log TP ~ Similarity + GC content + Tissue (Class II DNA transposons)* | |  |  |  |
|  | | |  |  |
|  | Estimate | Std. Error | t value | Pr(>\|t\|) |
| (Intercept) | -0.71615 | 1.80064 | -0.398 | 0.691 |
| Similarity | -0.02618 | 0.01832 | -1.429 | 0.155 |
| GC content | -0.01077 | 0.01302 | -0.827 | 0.409 |
| Tissue Stem | 0.07151 | 0.09313 | 0.768 | 0.443 |
| Residual SE = 0.6585 on 196 df, adjusted R² = 0.001895 | | | |  |
|  | | |  |  |

# **Table S8. Transcript proportions (TP) of repeats in the three replicate inflorescence poly-A RNA libraries of *Setaria viridis*.**

|  |  |  |  | **Inflorescence** |  |  |  |  |
| --- | --- | --- | --- | --- | --- | --- | --- | --- |
|  |  | Replicate 1 |  | Replicate 2 |  | Replicate 3 |  |  |
|  | **Total Reads** | 11,376,412 |  | 11,519,902 |  | 11,963,907 |  |  |
|  | **Total Organelles+ rDNA** | 136,153 |  | 215,602 |  | 232,897 |  |  |
| **Repeat Class/Superfamily** | **Total minus (Organelles+ rDNA)** | 11,240,259 |  | 11,304,300 |  | 11,731,010 |  |  |
| **Class I - Retrotransposons** | **Repeat Lineage** | **Reads mapped** | **TP %** | **Reads mapped** | **TP %** | **Reads mapped** | **TP %** | **Mean TP %** |
| ***Ty3/gypsy*** |  | **2213** | **0.020** | **2393** | **0.021** | **2182** | **0.019** | **0.020** |
|  | *Athila* | 168 | 0.001 | 141 | 0.001 | 165 | 0.001 | 0.001 |
|  | *CRM* | 247 | 0.002 | 140 | 0.001 | 176 | 0.002 | 0.002 |
|  | *Ogre* | 141 | 0.001 | 142 | 0.001 | 133 | 0.001 | 0.001 |
|  | *Reina* | 23 | 0.000 | 42 | 0.000 | 41 | 0.000 | 0.000 |
|  | *Retand* | 1485 | 0.013 | 1788 | 0.016 | 1545 | 0.013 | 0.014 |
|  | *Tekay* | 149 | 0.001 | 140 | 0.001 | 122 | 0.001 | 0.001 |
| ***Ty1/copia*** |  | **2154** | **0.019** | **2172** | **0.019** | **2696** | **0.023** | **0.020** |
|  | *Ale* | 555 | 0.005 | 487 | 0.004 | 437 | 0.004 | 0.004 |
|  | *Angela* | 102 | 0.001 | 106 | 0.001 | 135 | 0.001 | 0.001 |
|  | *Bianca* | 0 | 0.000 | 0 | 0.000 | 0 | 0.000 | 0.000 |
|  | *Ikeros* | 20 | 0.000 | 16 | 0.000 | 13 | 0.000 | 0.000 |
|  | *Ivana* | 290 | 0.003 | 279 | 0.002 | 252 | 0.002 | 0.002 |
|  | *SIRE* | 1121 | 0.010 | 1220 | 0.011 | 1786 | 0.015 | 0.012 |
|  | *TAR* | 52 | 0.000 | 56 | 0.000 | 61 | 0.001 | 0.000 |
|  | *Tork* | 14 | 0.000 | 8 | 0.000 | 12 | 0.000 | 0.000 |
| ***LINE*** |  | **884** | **0.008** | **957** | **0.008** | **1097** | **0.009** | **0.009** |
| ***Unclassified LTR*** |  | **1727** | **0.015** | **1748** | **0.015** | **1638** | **0.014** | **0.015** |
| ***Pararetrovirus*** |  | **0** | **0.000** | **2** | **0.000** | **3** | **0.000** | **0.000** |
| **Class II - DNA transposons** |  | **4093** | **0.036** | **4634** | **0.041** | **4788** | **0.041** | **0.039** |
|  | *CACTA* | 405 | 0.004 | 438 | 0.004 | 519 | 0.004 | 0.004 |
|  | *Helitron* | 16 | 0.000 | 24 | 0.000 | 31 | 0.000 | 0.000 |
|  | *Mariner* | 4 | 0.000 | 3 | 0.000 | 2 | 0.000 | 0.000 |
|  | *Mutator* | 3150 | 0.028 | 3636 | 0.032 | 3569 | 0.030 | 0.030 |
|  | *Harbinger* | 444 | 0.004 | 436 | 0.004 | 532 | 0.005 | 0.004 |
|  | *HAT* | 74 | 0.001 | 97 | 0.001 | 135 | 0.001 | 0.001 |
| **Satellite repeats** |  | **5** | **0.000** | **8** | **0.000** | **7** | **0.000** | **0.000** |
| **Unknown repeats** |  | **7073** | **0.063** | **7862** | **0.070** | **8123** | **0.069** | **0.067** |
| **Total** |  | **18149** | **0.161** | **19776** | **0.175** | **20534** | **0.175** | **0.170** |

# **Table S9. Transcript proportions (TP) of repeats in the three replicate ribo-depleted RNA libraries prepared from crown tissue of *Setaria viridis*.**

|  |  | **Crown ribo-depleted** | | | | |  |  |
| --- | --- | --- | --- | --- | --- | --- | --- | --- |
|  |  | Rep1 |  | Rep2 |  | Rep3 |  |  |
|  | **Total Reads** | 22,280,606 |  | 14,717,318 |  | 21,292,550 |  |  |
|  | **Total Organelles+ rDNA** | 6,125,278 |  | 6,253,478 |  | 2,063,390 |  |  |
| **Repeat Class/Superfamily** | **Total minus (Organelles+ rDNA)** | 16,155,328 |  | 8,463,840 |  | 19,229,160 |  |  |
| **Class I - Retrotransposons** | **Repeat Lineage** | **Reads mapped** | **TP %** | **Reads mapped** | **TP %** | **Reads mapped** | **TP %** | **Mean TP%** |
| ***Ty3/gypsy*** |  | **3502** | **0.022** | **4077** | **0.048** | **4219** | **0.022** | **0.031** |
|  | *Athila* | 448 | 0.003 | 439 | 0.005 | 409 | 0.002 | 0.003 |
|  | *CRM* | 289 | 0.002 | 461 | 0.005 | 414 | 0.002 | 0.003 |
|  | *Ogre* | 584 | 0.004 | 670 | 0.008 | 671 | 0.003 | 0.005 |
|  | *Reina* | 94 | 0.001 | 57 | 0.001 | 83 | 0.000 | 0.001 |
|  | *Retand* | 1434 | 0.009 | 1304 | 0.015 | 1815 | 0.009 | 0.011 |
|  | *Tekay* | 653 | 0.004 | 1146 | 0.014 | 827 | 0.004 | 0.007 |
| ***Ty1/copia*** |  | **5868** | **0.036** | **4660** | **0.055** | **7234** | **0.038** | **0.043** |
|  | *Ale* | 759 | 0.005 | 696 | 0.008 | 1105 | 0.006 | 0.006 |
|  | *Angela* | 879 | 0.005 | 1258 | 0.015 | 899 | 0.005 | 0.008 |
|  | *Bianca* | 3 | 0.000 | 5 | 0.000 | 0 | 0.000 | 0.000 |
|  | *Ikeros* | 258 | 0.002 | 241 | 0.003 | 246 | 0.001 | 0.002 |
|  | *Ivana* | 969 | 0.006 | 584 | 0.007 | 1069 | 0.006 | 0.006 |
|  | *SIRE* | 2644 | 0.016 | 1598 | 0.019 | 3578 | 0.019 | 0.018 |
|  | *TAR* | 138 | 0.001 | 101 | 0.001 | 150 | 0.001 | 0.001 |
|  | *Tork* | 218 | 0.001 | 177 | 0.002 | 187 | 0.001 | 0.001 |
| ***LINE*** |  | **1420** | **0.009** | **1033** | **0.012** | **1347** | **0.007** | **0.009** |
| ***Unclassified LTR*** |  | **6317** | **0.039** | **4788** | **0.057** | **6376** | **0.033** | **0.043** |
| ***Pararetrovirus*** |  | **48** | **0.000** | **70** | **0.001** | **29** | **0.000** | **0.000** |
| **Class II - DNA transposons** |  | **3566** | **0.022** | **3281** | **0.039** | **3238** | **0.017** | **0.026** |
|  | *CACTA* | 672 | 0.004 | 714 | 0.008 | 706 | 0.004 | 0.005 |
|  | *Helitron* | 14 | 0.000 | 20 | 0.000 | 17 | 0.000 | 0.000 |
|  | *Mariner* | 9 | 0.000 | 6 | 0.000 | 7 | 0.000 | 0.000 |
|  | *Mutator* | 2188 | 0.014 | 1981 | 0.023 | 1864 | 0.010 | 0.016 |
|  | *Harbinger* | 491 | 0.003 | 347 | 0.004 | 439 | 0.002 | 0.003 |
|  | *HAT* | 192 | 0.001 | 213 | 0.003 | 205 | 0.001 | 0.002 |
| **Satellite repeats** |  | **66** | **0.000** | **181** | **0.002** | **101** | **0.001** | **0.001** |
| **Unknown repeats** |  | **8792** | **0.054** | **5332** | **0.063** | **10320** | **0.054** | **0.057** |
| **Total** |  | **29579** | **0.183** | **23422** | **0.277** | **32864** | **0.171** | **0.2102** |

# **Table S10. Transcript proportions (TP) of repeats in the three replicate ribo-depleted RNA libraries prepared from stem tissue of *Setaria viridis*.**

|  |  | **Stem ribo-depleted** | | | | | |  |
| --- | --- | --- | --- | --- | --- | --- | --- | --- |
|  |  | Rep1 |  | Rep2 |  | Rep3 |  |  |
|  | **Total Reads** | 34,113,920 |  | 20,053,518 |  | 30,755,030 |  |  |
|  | **Total (Organelles+ rDNA)** | 14,533,132 |  | 7,080,727 |  | 3,581,976 |  |  |
| **Repeat Class/Superfamily** | **Total minus (Organelles+ rDNA)** | 19,580,788 |  | 12,972,791 |  | 27,173,054 |  |  |
| **Class I - Retrotransposons** | **Repeat Lineage** | **Reads mapped** | **TP %** | **Reads mapped** | **TP %** | **Reads mapped** | **TP %** | **Mean TP%** |
| ***Ty3/gypsy*** |  | **2961** | **0.015** | **12162** | **0.094** | **6136** | **0.023** | **0.044** |
|  | *Athila* | 391 | 0.002 | 1167 | 0.009 | 566 | 0.002 | 0.004 |
|  | *CRM* | 254 | 0.001 | 1358 | 0.010 | 602 | 0.002 | 0.005 |
|  | *Ogre* | 551 | 0.003 | 2205 | 0.017 | 1057 | 0.004 | 0.008 |
|  | *Reina* | 84 | 0.000 | 99 | 0.001 | 127 | 0.000 | 0.001 |
|  | *Retand* | 1179 | 0.006 | 3515 | 0.027 | 2366 | 0.009 | 0.014 |
|  | *Tekay* | 502 | 0.003 | 3818 | 0.029 | 1418 | 0.005 | 0.012 |
| ***Ty1/copia*** |  | **6139** | **0.031** | **12373** | **0.095** | **9304** | **0.034** | **0.054** |
|  | *Ale* | 751 | 0.004 | 1495 | 0.012 | 1343 | 0.005 | 0.007 |
|  | *Angela* | 669 | 0.003 | 3439 | 0.027 | 1255 | 0.005 | 0.012 |
|  | *Bianca* | 0 | 0.000 | 30 | 0.000 | 4 | 0.000 | 0.000 |
|  | *Ikeros* | 289 | 0.001 | 441 | 0.003 | 408 | 0.002 | 0.002 |
|  | *Ivana* | 552 | 0.003 | 1190 | 0.009 | 1037 | 0.004 | 0.005 |
|  | *SIRE* | 3645 | 0.019 | 5103 | 0.039 | 4939 | 0.018 | 0.025 |
|  | *TAR* | 112 | 0.001 | 308 | 0.002 | 186 | 0.001 | 0.001 |
|  | *Tork* | 121 | 0.001 | 367 | 0.003 | 132 | 0.000 | 0.001 |
| ***LINE*** |  | **1348** | **0.007** | **1678** | **0.013** | **1792** | **0.007** | **0.009** |
| ***Unclassified LTR*** |  | **4807** | **0.025** | **10153** | **0.078** | **6463** | **0.024** | **0.042** |
| ***Pararetrovirus*** |  | **10** | **0.000** | **53** | **0.000** | **12** | **0.000** | **0.000** |
| **Class II - DNA transposons** |  | **3403** | **0.017** | **6986** | **0.054** | **4332** | **0.016** | **0.029** |
|  | *CACTA* | 652 | 0.003 | 2734 | 0.021 | 1320 | 0.005 | 0.010 |
|  | *Helitron* | 18 | 0.000 | 39 | 0.000 | 28 | 0.000 | 0.000 |
|  | *Mariner* | 3 | 0.000 | 14 | 0.000 | 3 | 0.000 | 0.000 |
|  | *Mutator* | 2070 | 0.011 | 2965 | 0.023 | 2134 | 0.008 | 0.014 |
|  | *Harbinger* | 463 | 0.002 | 737 | 0.006 | 574 | 0.002 | 0.003 |
|  | *HAT* | 197 | 0.001 | 497 | 0.004 | 273 | 0.001 | 0.002 |
| **Satellite repeats** |  | **38** | **0.000** | **1312** | **0.010** | **816** | **0.003** | **0.004** |
| **Unknown repeats** |  | **9339** | **0.048** | **11995** | **0.092** | **15163** | **0.056** | **0.065** |
| **Total** |  | **28045** | **0.1432** | **56712** | **0.4372** | **44018** | **0.162** | **0.2475** |
