# Supplementary material for: Contrasting distributions and expression characteristics of transcribing repeats in Setaria viridis
Source: Plant Genome. 2025 Jan 9;18(1):e20551. doi: 10.1002/tpg2.20551 (PMC11718148; doi:10.1002/tpg2.20551)
Supplement: Supplementary file 1 — Figure S1. Cluster graphs showing examples of repeat clusters (CL) identified in Setaria viridis using RepeatExplorer2. Figure S2. Examples of repeat sequence similarity scores (%) of reads from two of the 208 repeat clusters generated by RepeatExplorer2 using genomic DNA of Setaria viridis. Figure S3. Scatterplot showing the genomic characteristics of the repeat clusters. Figure S4. Scatterplots showing the relationship between the transcriptome proportion (TP) and the genomic characteristics for repeat clusters in poly‐A libraries of inflorescence tissue. Figure S5. Scatterplots showing the relationship between the transcriptome proportion (TP) and the genomic characteristics for repeat clusters in ribo‐depleted libraries from crown and stem tissues. Figure S6. Pipelines used to quantify and characterize repeats in the genome and transcriptomes of Setaria viridis. Figure S7. RNAseq reads mapped in genome assembly. (a) Number of full‐length and incomplete repeats annotated in the genome using DANTE and the proportion of those intronics and non‐intronics (b) Heatmap showing the repeats differentially expressed according to Deseq2 (c) IGV screenshot of CRM type up regulated in ribo‐depleted leaf (d) IGV screenshot of repeats in intronic regions [file TPG2-18-e20551-s002.docx]

**Supplemental Figures**

[**Supplemental Figures** 2](#_Toc163303868)

[**Figure S1**. Cluster graphs showing examples of repeat clusters (CL) identified in *Setaria viridis* using RepeatExplorer2. 2](#_Toc163303869)

[**Figure S2.** Examples of repeat sequence similarity scores (%) of reads from two of the 208 repeat clusters generated by RepeatExplorer2 using genomic DNA of *Setaria viridis*. 3](#_Toc163303870)

[**Figure S3.** Scatterplot showing the genomic characteristics of the repeat clusters. 4](#_Toc163303871)

[**Figure S4**. Scatterplot showing the relationship between the transcriptome proportion (TP) and the genomic characteristics for repeat clusters in poly-A libraries of inflorescence tissue. 5](#_Toc163303872)

[**Figure S5.** Scatterplot showing the relationship between the transcriptome proportion (TP) and the genomic characteristics for repeat clusters in ribo-depleted libraries from crown and stem tissues. 6](#_Toc163303873)

[**Figure S6**. Pipelines used to quantify repeats in the genome and transcriptomes of *Setaria viridis*. 7](#_Toc163303874)

[**Figure S7**. RNAseq reads mapped in genome assembly. (a) Number of full-length and incomplete repeats annotated in the genome using DANTE and the proportion of those intronics and non-intronics (b) Heatmap showing the repeats differentially expressed according to Deseq2 (c) IGV screenshot of CRM type up regulated in ribo-depleted leaf (d) IGV screenshot of repeats in intronic regions. 8](#_Toc163303875)

# Supplemental Figures


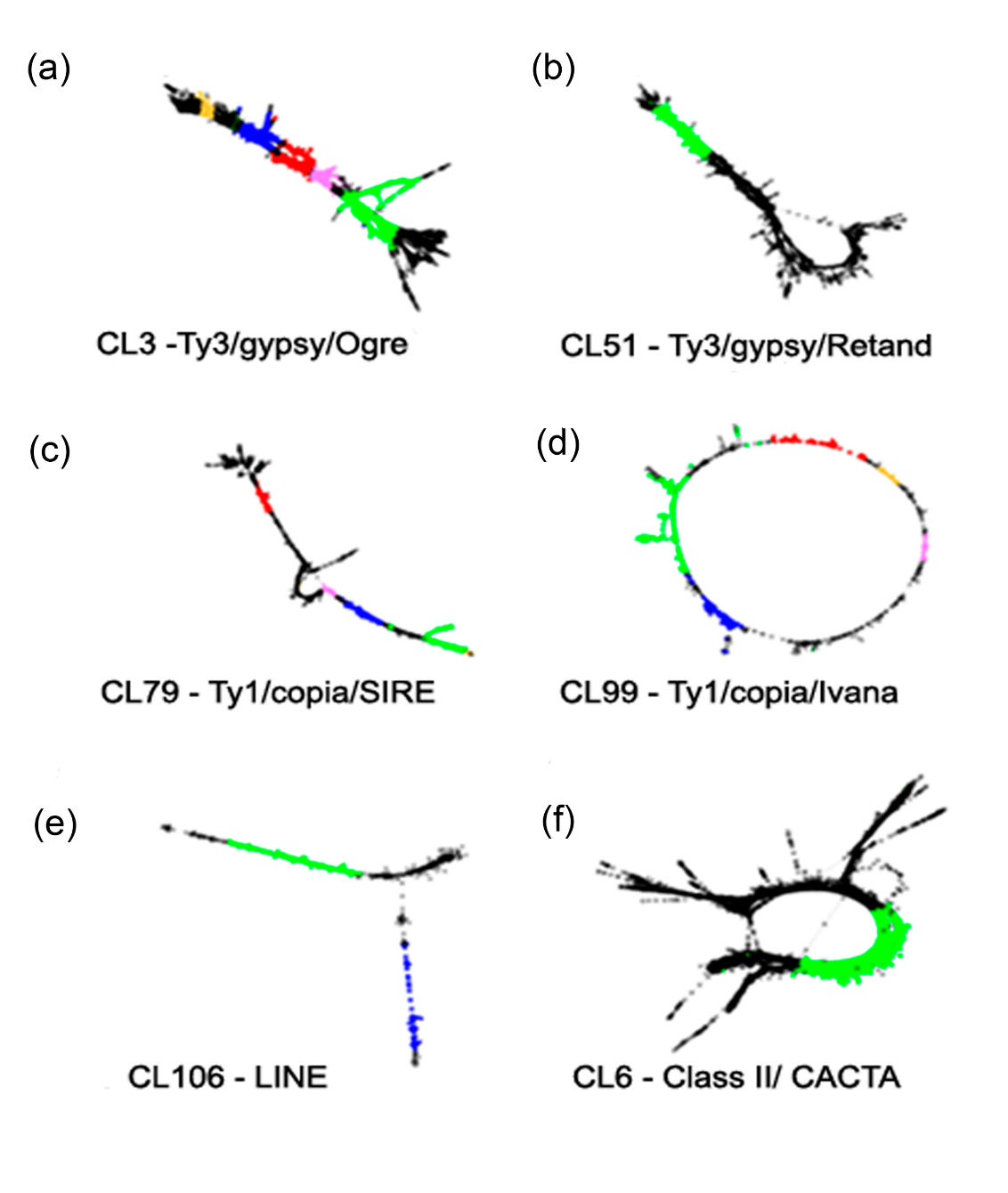


# Figure S1. Cluster graphs showing examples of repeat clusters (CL) identified in *Setaria viridis* using RepeatExplorer2. The clusters represent repeats that are transcribed in all RNA libraries analysed. The colours correspond to different protein domains. (a, b) Ty3/gypsy elements: green = INT (integrase), blue = RT (reverse transcriptase), red = RH (ribonuclease H), pink = aRH (archeal ribonuclease), yellow = PROT (protease). (c, d) Ty1/copia elements: green = RT, blue = RH, red = INT, pink = GAG, yellow = PROT. e) LINE element: green = LINE RT, blue = ENDO (endonuclease). (f) CACTA DNA transposon: green = transposase


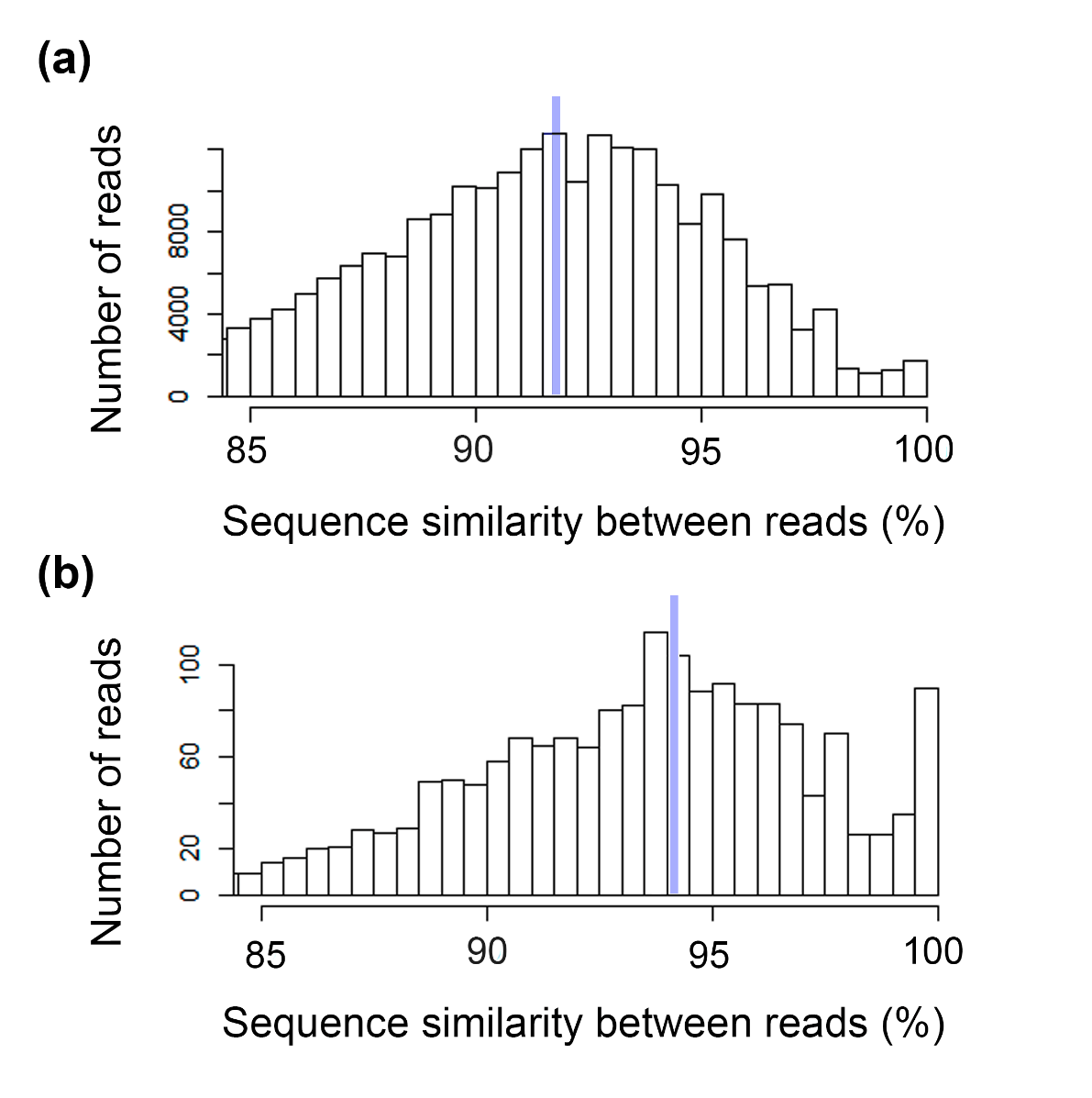


# Figure S2. Examples of repeat sequence similarity scores (%) of reads from two of the 208 repeat clusters generated by RepeatExplorer2 using genomic DNA of *Setaria viridis*.

(a) The third most abundant repeat (CL3) is a Ty3/gypsy Ogre element and has a repeat similarity score of 91.89% (modal value – indicated by the blue line). (b) Cluster 99 is a Ty1/copia Ivana element with a modal repeat similarity score of 94.21%.


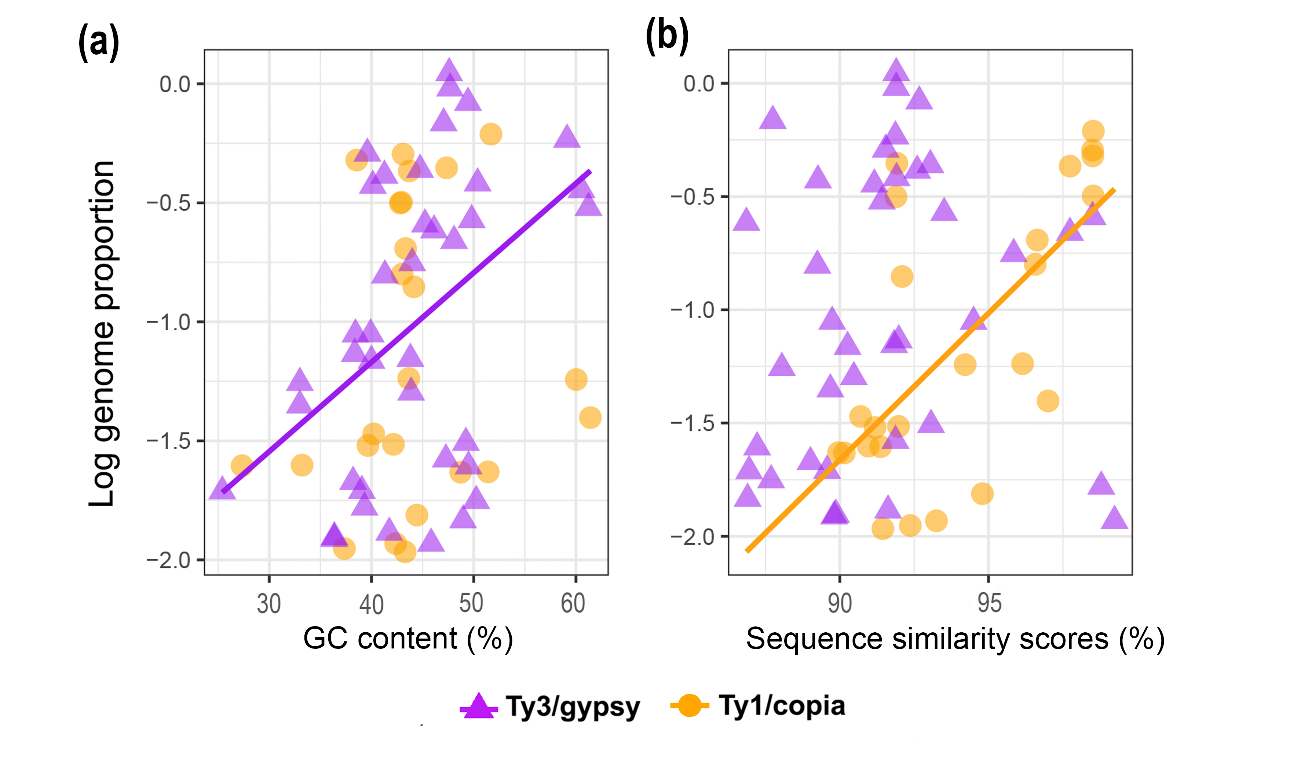



# Figure S3. Scatterplot showing the genomic characteristics of the repeat clusters identified using RepeatExplorer2. The relationships between (a) genome proportion (GP) and GC content, and (b) GP and repeat sequence similarity scores for Ty1/copia and Ty3/gypsy repeat clusters. Only significant slopes are shown (see Table S2).


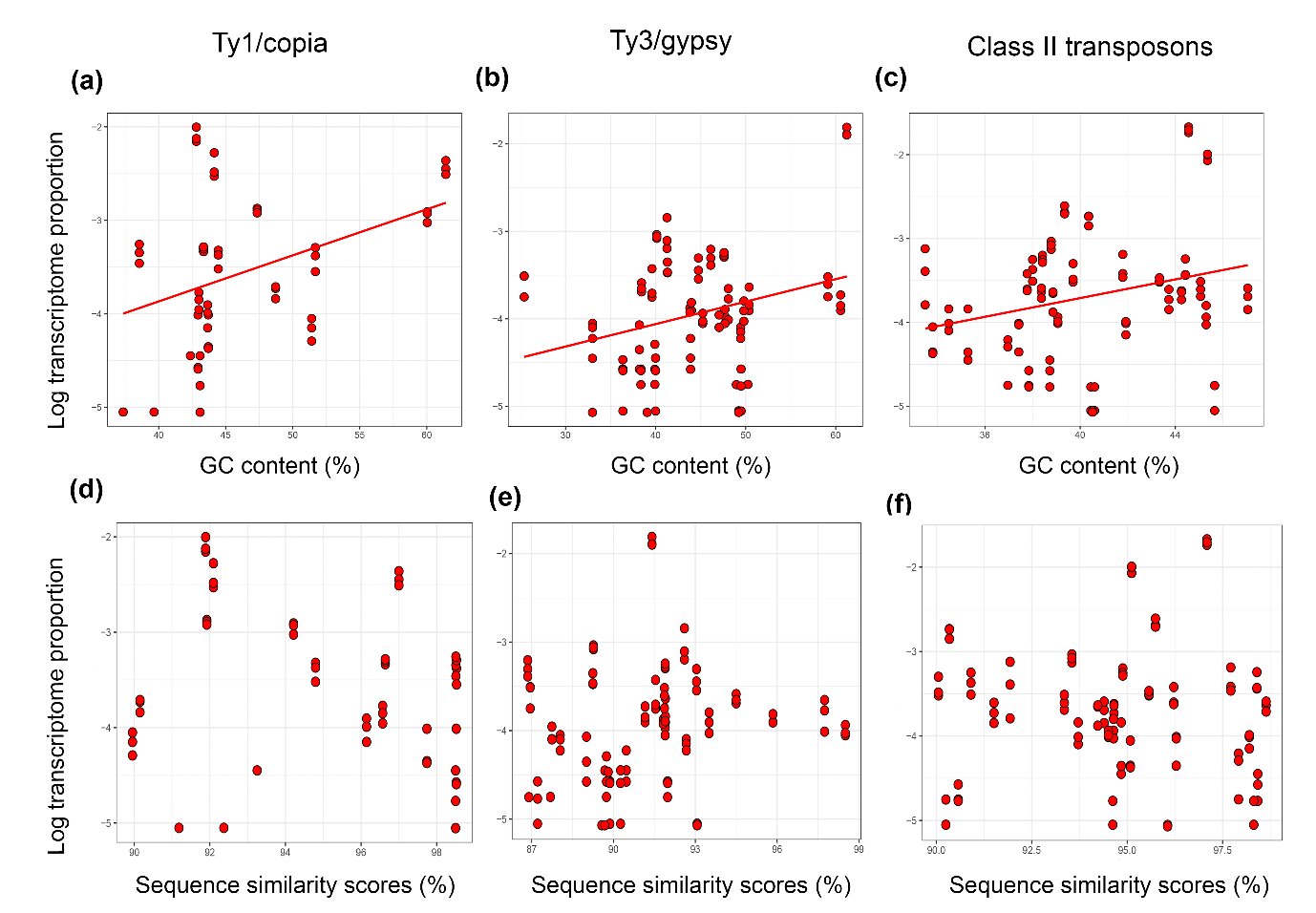


# Figure S4. Scatterplots showing the relationship between the transcriptome proportion (TP) and the genomic characteristics for repeat clusters in poly-A libraries of inflorescence tissue.

The relationship between TP of (a, d) Ty1/copia, (b, e) Ty3/gypsy and (c, f) class II DNA transposons and GC content (a-c) and modal repeat sequence similarity scores (d-f). For slopes and coefficients see Table S7d-f.


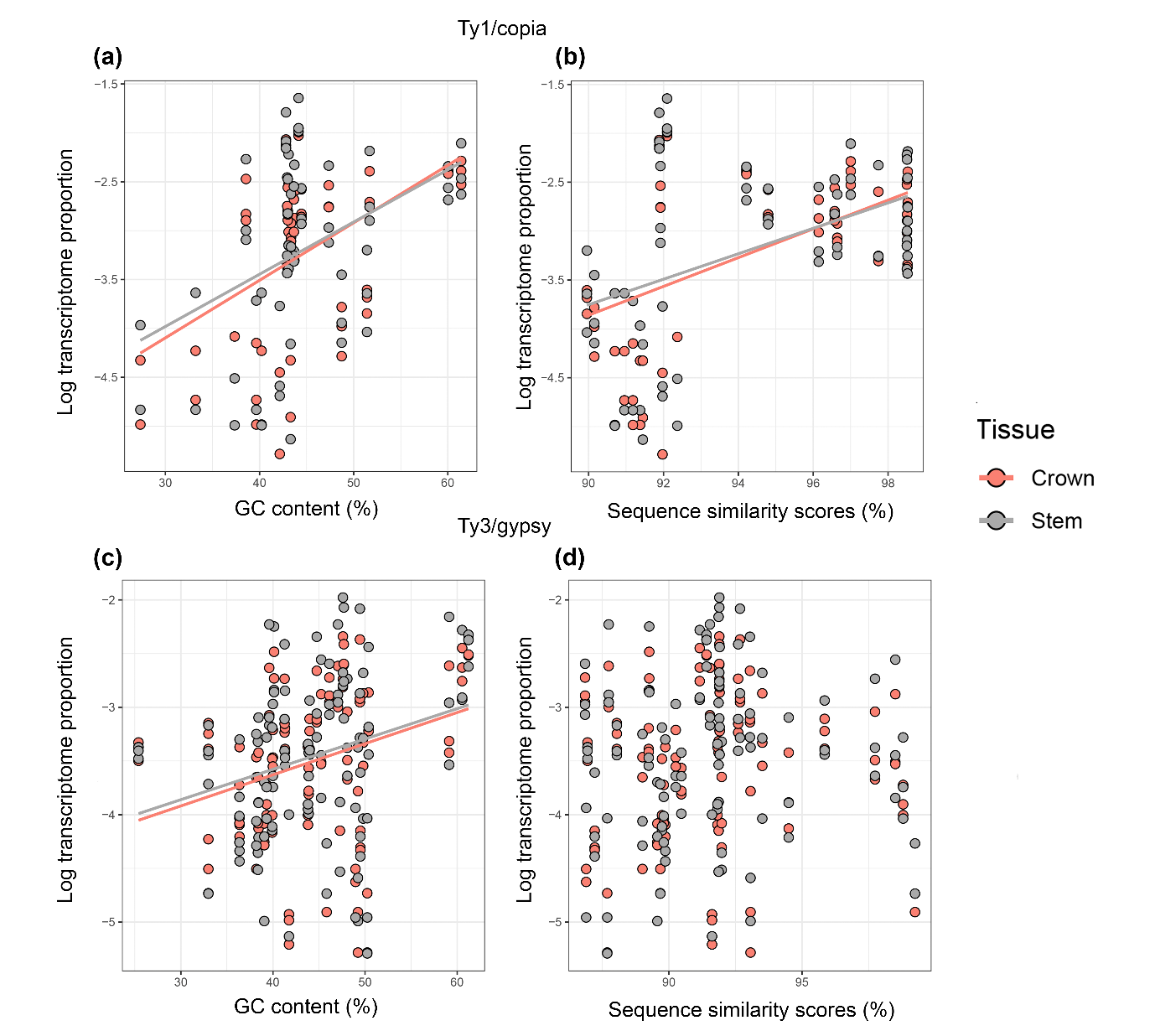


# Figure S5. Scatterplots showing the relationship between the transcriptome proportion (TP) and the genomic characteristics for repeat clusters in ribo-depleted libraries from crown and stem tissues. The relationship between TP of (a, b) Ty1/copia and (c, d) Ty3/gypsy and GC content (a, c) and modal repeat sequence similarity scores (b, d). For slopes and coefficients see Table S7 j-l.

# Figure S6. Pipelines used to quantify and characterize repeats in the genome and transcriptomes of *Setaria viridis*.

(a) Analysis pipeline for repeats in genome skimming data using RepeatExlorer2. (b Analysis pipeline for characterizing complete and incomplete LTR retroelements in the whole genome assembly data from Mamidi et al. (2020). Input data and generated data are in orange boxes while the bioinformatic tools used are in yellow boxes.
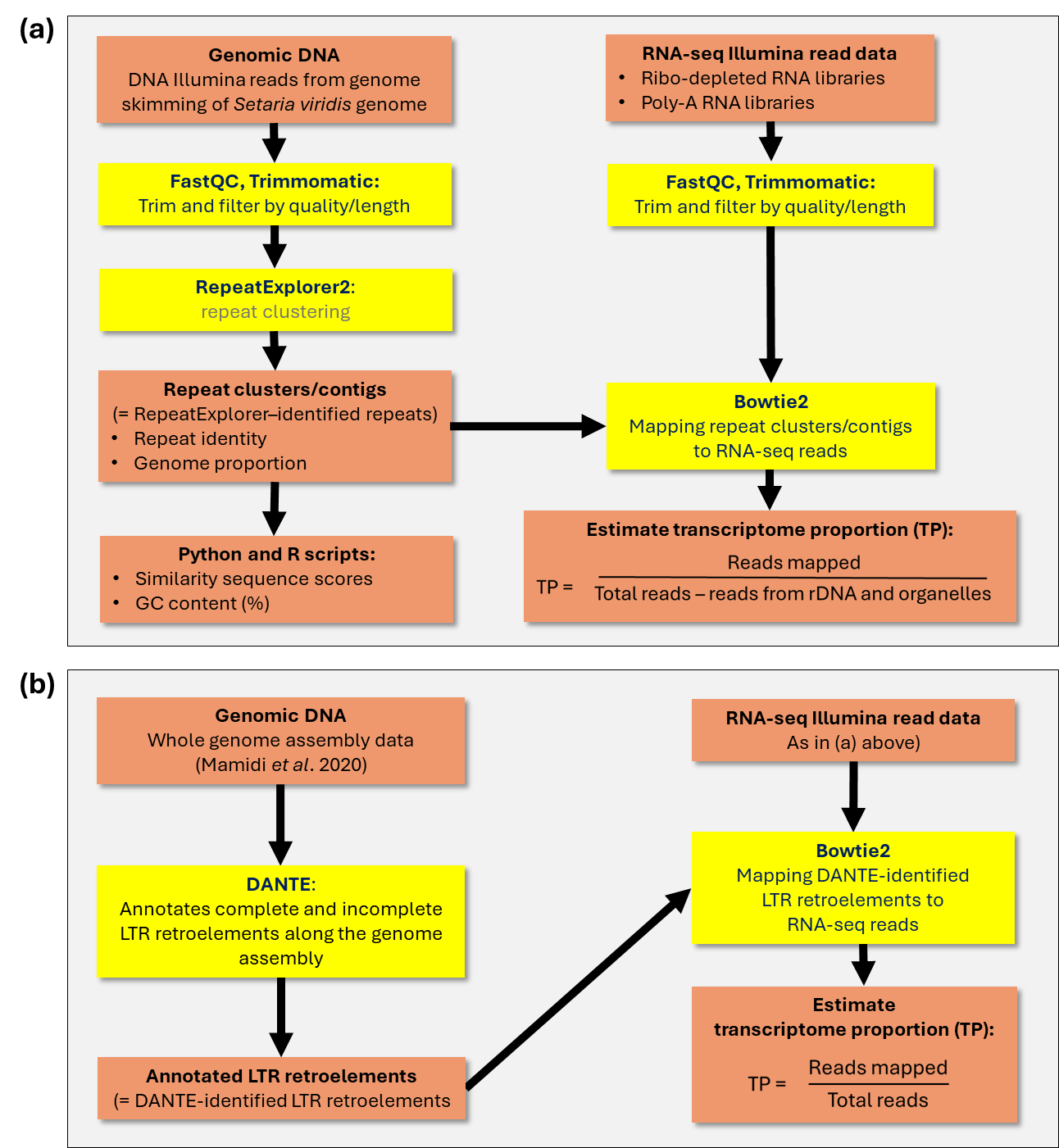


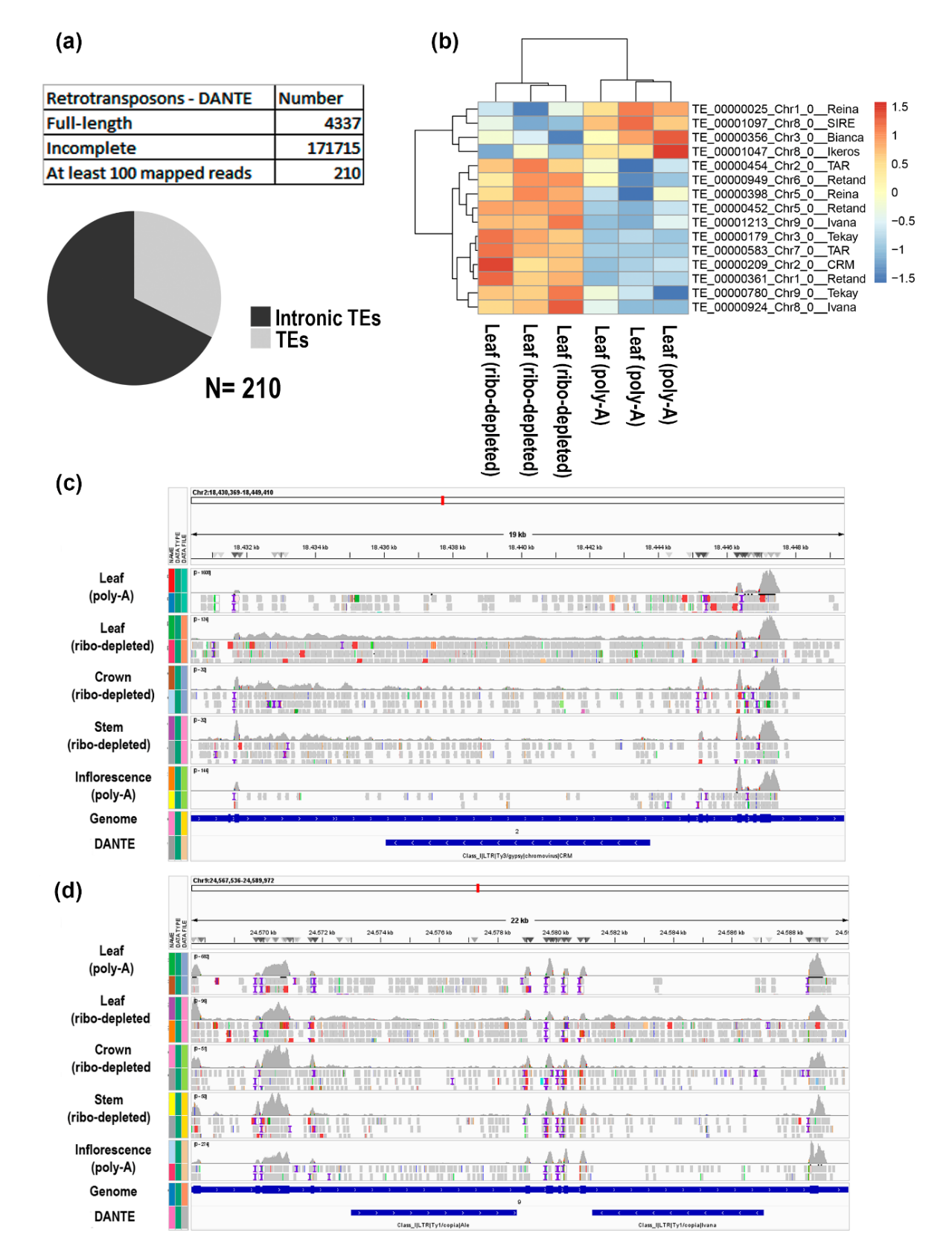


# Figure S7. RNA-seq reads mapped to the whole genome assembly of *Setaria viridis* ‘A10’. (a) Number of complete and incomplete LTR retroelements identified and annotated in the genome using DANTE, and the proportion of those that are intronic and non-intronic. (b) Heatmap showing the 15 LTR retroelements that are differentially expressed between the poly-A and ribo-depleted transcriptomes from leaf material (three replicas of each library type), analysed using Deseq2. (c) Integrative genomic viewer (IGV) screenshot of transcribed Ty3/gypsy CRM retroelements that are up-regulated in ribo-depleted leaf transcriptomes compared with the poly-A libraries from leaf material. (d) IGV screenshot of transcribed Ty1/copia Ale and Ivana retroelements in intronic regions.
