# Supplementary material for: Contrasting distributions and expression characteristics of transcribing repeats in Setaria viridis
Source: Plant Genome. 2025 Jan 9;18(1):e20551. doi: 10.1002/tpg2.20551 (PMC11718148; doi:10.1002/tpg2.20551)
Supplement: Supplementary file 2 — Table S1. NCBI accession numbers and sources of DNA and RNA sequence read datasets of Setaria viridis ‘A10’ used in this study. Table S2. Relationships between GP, GC ratio and sequence similarities of repeat reads. Table S3. Number of complete and incomplete elements annotated in the entire Setaria viridis genome by DANTE software. Table S4. Transcript proportions (TP) of repeats in the three replicate ribo‐depleted RNA libraries prepared from leaf tissue of Setaria viridis. Table S5. Transcript proportions (TP) of repeats in three‐replicate poly‐A RNA libraries prepared from leaf tissue of Setaria viridis. Table S6. Linear regression models analysing the relationship between repeat (Ty1/copia, Ty3/gypsy and DNA transposon) transcriptome proportions (TP) in leaf poly‐A (intercept) and ribo‐depleted libraries and their genome proportion (GP), GC content and sequence similarities. Table S7. Linear regression models analysing the relationship between repeat transcriptome proportions (TP) for different RNA libraries, tissues (stem, crown and inflorescence) and genomic features of Ty1/copia, Ty3/gypsy and DNA transposon repeats. Table S8. Transcript proportions (TPs) of repeats in the three replicate inflorescence poly‐A RNA libraries of Setaria viridis. Table S9. Transcript proportions (TPs) of repeats in the three replicate ribo‐depleted RNA libraries prepared from crown tissue of Setaria viridis. Table S10. Transcript proportions (TPs) of repeats in the three replicate ribo‐depleted RNA libraries prepared from stem tissue of Setaria viridis. [file TPG2-18-e20551-s001.docx]

[**Supplemental Tables** 2](#_Toc163168184)

[**Table S1.** NCBI accession numbers and sources of DNA and RNA sequence read datasets of *Setaria viridis* ‘A10’ used in this study. 2](#_Toc163168185)

[**Table S2.** Relationships between GP, GC ratio and sequence similarities of repeat reads. 4](#_Toc163168186)

[**Table S3**. Number of complete and incomplete elements annotated in the entire *Setaria viridis* genome by DANTE software. 6](#_Toc163168187)

[**Table S4**. Transcript proportions (TP) of repeats in the three replicate ribo-depleted RNA libraries prepared from leaf tissue of *Setaria viridis*. 7](#_Toc163168188)

[**Table S5**. Transcript proportions (TP) of repeats in three-replicate poly-A RNA libraries prepared from leaf tissue of *Setaria viridis*. 9](#_Toc163168189)

[**Table S6**. Linear regression models analysing the relationship between repeat (Ty1/copia, Ty3/gypsy and DNA transposon) transcriptome proportions (TP) in leaf poly-A (intercept) and ribo-depleted libraries and their genome proportion (GP), GC content and sequence similarities. 11](#_Toc163168190)

[**Table S7**. Linear regression models analysing the relationship between repeat transcriptome proportions (TP) for different RNA libraries, tissues (stem, crown and inflorescence) and genomic features of Ty1/copia, Ty3/gypsy and DNA transposon repeats. 13](#_Toc163168191)

[**Table S8**. Transcript proportions (TPs) of repeats in the three replicate inflorescence poly-A RNA libraries of *Setaria viridis*. 16](#_Toc163168192)

**[Table S9](#_Toc163168193)**[. Transcript proportions (TPs) of repeats in the three replicate ribo-depleted RNA libraries prepared from crown tissue of](#_Toc163168193) *[Setaria viridis](#_Toc163168193)*[. 18](#_Toc163168193)

**[Table S10](#_Toc163168194)**[. Transcript proportions (TPs) of repeats in the three replicate ribo-depleted RNA libraries prepared from stem tissue of](#_Toc163168194) *[Setaria viridis](#_Toc163168194)*[. 20](#_Toc163168194)

# **Supplemental Tables**

# **Table S1. NCBI accession numbers and sources of DNA and RNA sequence read datasets of *Setaria viridis* ‘A10’ used in this study.**

**The original sources of the data are given in the column ‘Reference’. Descriptions of the library (taken from the source papers), the library layout (single- or paired-end reads), the library type (genomic, ribo-depleted or poly-A), the tissue source of the library and the numbers of reads analysed are shown.**

| **Accession number in NCBI** | **Description** | **Library layout** | **Library Type** | **Tissue** | **Number of reads after filtering** | **Reference** |
| --- | --- | --- | --- | --- | --- | --- |
| SRR10051273 | TB_setaria_16_0782 | Paired-end | Genomic DNA | Leaf | 74,498,042 | Mamidi et al. 2020^1^ |
| SRR26158246 | wild-type rep1 | paired-end | Ribo-depleted | Leaf | 183,624,756 | This study |
| SRR26158245 | wild-type rep2 | paired-end | Ribo-depleted | Leaf | 115,123,222 | This study |
| SRR26158244 | wild-type rep3 | paired-end | Ribo-depleted | Leaf | 184,007,030 | This study |
| SRR26158243 | wild-type rep1 | paired-end | Poly-A | Leaf | 151,136,544 | This study |
| SRR26158242 | wild-type rep2 | paired-end | Poly-A | Leaf | 164,751,814 | This study |
| SRR26158241 | wild-type rep3 | paired-end | Poly-A | Leaf | 194,996,794 | This study |
| SRR3176660 | 9 DAS rep1 watered | paired-end | Ribo-depleted | Upper region (stem) | 34,113,920 | Sebastian et al. 2016^2^ |
| SRR3176668 | 9 DAS rep2 watered | paired-end | Ribo-depleted | Upper region (stem) | 20,053,518 | Sebastian et al. 2016^2^ |
| SRR3176676 | 9 DAS rep3 watered | paired-end | Ribo-depleted | Upper region (stem) | 30,755,030 | Sebastian et al. 2016^2^ |
| SRR3176661 | 9 DAS rep1 watered | paired-end | Ribo-depleted | Lower region (crown) | 22,280,606 | Sebastian et al. 2016^2^ |
| SRR3176669 | 9 DAS rep2 watered | paired-end | Ribo-depleted | Lower region (crown) | 14,717,318 | Sebastian et al. 2016^2^ |
| SRR3176677 | 9 DAS rep3 watered | paired-end | Ribo-depleted | Lower region (crown) | 21,292,550 | Sebastian et al. 2016^2^ |
| SRR5750472 | wild-type rep1 | single-end | Poly-A | Inflorescence primordia | 11,376,412 | Yang et al. 2018^3^ |
| SRR5750473 | wild-type rep2 | single-end | Poly-A | Inflorescence primordia | 11,519,902 | Yang et al. 2018^3^ |
| SRR5750474 | wild-type rep3 | single-end | Poly-A | Inflorescence primordia | 11,963,907 | Yang et al. 2018^3^ |

**^1^Mamidi, S., Healey, A., Huang, P., et al.** (2020) A genome resource for green millet *Setaria viridis* enables discovery of agronomically valuable loci. *Nat. Biotechnol.*, **38**, 1203–1210.

**^2^Sebastian, J., Yee, M., Goudinho, W., et al.** (2016) Grasses suppress shoot-borne roots to conserve water during drought. *Proc. Natl. Acad. Sci.*, **113**, 8861–8866.

**^3^Yang, J., Thames, S., Best, N.B., Jiang, H., Huang, P., Dilkes, B.P. and Eveland, A.L.** (2018) Brassinosteroids modulate meristem fate and differentiation of unique inflorescence morphology in *Setaria viridis*. *Plant Cell*, **30**, 48–66

# **Table S2. Relationships between GP, GC ratio and sequence similarities of repeat reads.**

**The baseline level (intercept) is the Ty3/gypsy element in each case. Std Error = standard error; df= degrees of freedom and adjusted R² of the models are reported.**

| ***a****) Log GP ~ GC content (DNA transposons)* |  |  |  |  |
| --- | --- | --- | --- | --- |
|  | Estimate | Std. Error | t value | Pr(>\|t\|) |
| (Intercept) | -2.60307 | 0.97929 | -2.658 | 0.0122 * |
| GC content | 0.03153 | 0.02417 | 1.304 | 0.2014 |
| Residual SE= 0.4993 on 32 on df, R²adjusted 0.0208 | |  |  |  |
|  |  |  |  |  |
| ***b)*** *Log GP ~ GC content (Ty3/gypsy)* |  |  |  |  |
|  | Estimate | Std. Error | t value | Pr(>\|t\|) |
| (Intercept) | -2.68956 | 0.59556 | -4.516 | 6.22e-05 *** |
| GC content | 0.0377 | 0.01332 | 2.831 | 0.00746 ** |
| Residual SE= 0.6002 on 37 df, adjusted R²= 0.1558 | |  |  |  |
|  |  |  |  |  |
| ***c)*** *Log GP ~ GC content (Ty1/copia )* |  |  |  |  |
|  | Estimate | Std. Error | t value | Pr(>\|t\|) |
| (Intercept) | -1.66968 | 0.79983 | -2.088 | 0.0486 * |
| GC content | 0.0118 | 0.01795 | 0.657 | 0.5178 |
| Residual SE= 0.6271 on 22 df, adjusted R²= -0.02532 | |  |  |  |
|  |  |  |  |  |
| **d)** *Log GP~ Similarity (DNA transposons)* |  |  |  |  |
|  | Estimate | Std. Error | t value | Pr(>\|t\|) |
| (Intercept) | 2.04369 | 3.24786 | 0.629 | 0.534 |
| Similarity | -0.03561 | 0.03426 | -1.039 | 0.306 |
| Residual SE= 0.504 on 32 df, adjusted R²= 0.002424 | |  |  |  |
|  |  |  |  |  |
| ***e)*** *Log GP ~ Similarity (Ty3/gypsy)* |  |  |  |  |
|  | Estimate | Std. Error | t value | Pr(>\|t\|) |
| (Intercept) | -3.75871 | 3.00232 | -1.252 | 0.218 |
| Similarity | 0.02988 | 0.03281 | 0.911 | 0.368 |
| Residual SE= 0.6548 on 37 df, adjusted R²= -0.004502 | |  |  |  |
|  |  |  |  |  |
| ***f)*** *Log GP ~ Similarity (Ty1/copia)* |  |  |  |  |
|  | Estimate | Std. Error | t value | Pr(>\|t\|) |
| (Intercept) | -13.79437 | 3.02696 | -4.557 | 0.000155 *** |
| Similarity | 0.13448 | 0.03218 | 4.179 | 0.000390 *** |
| Residual SE= 0.4728 on 22 df, adjusted R²= 0.4172 | |  |  |  |
|  |  |  |  |  |
| **g)** *GC content~ Similarity*Repeat Type* |  |  |  |  |
|  | Estimate | Std. Error | t value | Pr(>\|t\|) |
| (Intercept - Copia) | -7.122 | 46.6431 | -0.153 | 0.879 |
| Similarity | 0.5437 | 0.4659 | 1.096 | 0.277 |
| Repeat Type Gypsy | 16.5194 | 57.3704 | 0.288 | 0.774 |
| Similarity:Repeat Type Gypsy | 0.001639 | 0.6157 | -0.266 | 0.791 |
| Residual SE= 7.285 on 59 df, adjusted R²= -0.01158 | |  |  |  |
|  | |  |  |  |

# **Table S3. Number of complete and incomplete elements annotated in the entire *Setaria viridis* genome by DANTE software**

| **Lineage** | **LTR  mean length  [bp]** | **Element mean length  [bp]** | **Incomplete LTR retroelements** | | | | **Complete LTR retroelements** |
| --- | --- | --- | --- | --- | --- | --- | --- |
|  |  |  | Number with no LTR detected | Number with with just LTR | Number with just LTR & PBS | Number with just LTR & TSD | Number with LTR, PBS, TSD |
| Class_I\|LTR\|Ty1/copia\|Ale | 188 | 5053 | 500 | 130 | 136 | 322 | 81 |
| Class_I\|LTR\|Ty1/copia\|Alesia | 336 | 4889 | 6 | 0 | 2 | 0 | 0 |
| Class_I\|LTR\|Ty1/copia\|Angela | 1408 | 8080 | 723 | 136 | 697 | 1026 | 486 |
| Class_I\|LTR\|Ty1/copia\|Bianca | 232 | 6238 | 43 | 8 | 9 | 14 | 6 |
| Class_I\|LTR\|Ty1/copia\|Ikeros | 448 | 6957 | 77 | 23 | 67 | 128 | 51 |
| Class_I\|LTR\|Ty1/copia\|Ivana | 347 | 5466 | 122 | 20 | 64 | 122 | 48 |
| Class_I\|LTR\|Ty1/copia\|SIRE | 1415 | 10846 | 153 | 33 | 58 | 80 | 33 |
| Class_I\|LTR\|Ty1/copia\|TAR | 741 | 6201 | 126 | 37 | 58 | 88 | 31 |
| Class_I\|LTR\|Ty1/copia\|Tork | 471 | 5166 | 66 | 9 | 35 | 60 | 29 |
| Class_I\|LTR\|Ty3/gypsy\| chromovirus\|CRM | 667 | 7070 | 265 | 77 | 175 | 82 | 31 |
| Class_I\|LTR\|Ty3/gypsy\| chromovirus\|Reina | 318 | 5621 | 157 | 26 | 68 | 116 | 45 |
| Class_I\|LTR\|Ty3/gypsy\| chromovirus\|Tekay | 2468 | 12554 | 797 | 295 | 589 | 712 | 323 |
| Class_I\|LTR\|Ty3/gypsy\| non-chromovirus\|OTA\|Athila | 989 | 10024 | 259 | 8 | 4 | 10 | 0 |
| Class_I\|LTR\|Ty3/gypsy\|non-chromovirus\|OTA\|Tat\|Ogre | 1117 | 13404 | 678 | 222 | 86 | 666 | 17 |
| Class_I\|LTR\|Ty3/gypsy\|non-chromovirus\|OTA\|Tat\|Retand | 699 | 13062 | 2142 | 254 | 435 | 682 | 297 |

LTR = long terminal repeats; PBS = primer binding site, TSD = target site duplication, bp = base pairs.

# **Table S4. Transcript proportions (TP) of repeats in the three replicate ribo-depleted RNA libraries prepared from leaf tissue of *Setaria viridis*.**

**The material is the same as in Table S5, differing only in the type of library prepared.**

|  |  | **Leaf ribo-depleted** | | | | | |  |
| --- | --- | --- | --- | --- | --- | --- | --- | --- |
|  |  | Replicate 1 |  | Replicate 2 |  | Replicate 3 |  |  |
|  | **Total Reads** | 183,624,756 |  | 115,123,222 |  | 184,007,030 |  |  |
|  | **Total Organelles+ rDNA+ tRNA** | 167,246,165 |  | 107,420,926 |  | 171,470,160 |  |  |
| **Repeat Class/Superfamily** | **Total minus (Organelles+ rDNA  + tRNA)** | 16,378,591 |  | 7,702,296 |  | 12,536,870 |  |  |
| **Class I –**  **RNA retroelements** | **Repeat Lineage** | **Reads mapped** | **TP %** | **Reads mapped** | **TP %** | **Reads mapped** | **TP %** | **Mean TP %** |
| ***Ty3/gypsy*** |  | **11763** | **0.072** | **5866** | **0.076** | **11102** | **0.089** | **0.079** |
|  | *Athila* | 749 | 0.005 | 446 | 0.006 | 688 | 0.005 | 0.005 |
|  | *CRM* | 660 | 0.004 | 435 | 0.006 | 845 | 0.007 | 0.005 |
|  | *Ogre* | 1161 | 0.007 | 678 | 0.009 | 1323 | 0.011 | 0.009 |
|  | *Reina* | 46 | 0.000 | 21 | 0.000 | 35 | 0.000 | 0.000 |
|  | *Retand* | 5755 | 0.035 | 2195 | 0.028 | 4475 | 0.036 | 0.033 |
|  | *Tekay* | 3392 | 0.021 | 2091 | 0.027 | 3736 | 0.030 | 0.026 |
| ***Ty1/copia*** |  | **7325** | **0.045** | **3710** | **0.048** | **5813** | **0.046** | **0.046** |
|  | *Ale* | 1618 | 0.010 | 652 | 0.008 | 899 | 0.007 | 0.009 |
|  | *Angela* | 1409 | 0.009 | 801 | 0.010 | 1546 | 0.012 | 0.010 |
|  | *Bianca* | 13 | 0.000 | 30 | 0.000 | 31 | 0.000 | 0.000 |
|  | *Ikeros* | 236 | 0.001 | 112 | 0.001 | 194 | 0.002 | 0.001 |
|  | *Ivana* | 1662 | 0.010 | 777 | 0.010 | 1326 | 0.011 | 0.010 |
|  | *SIRE* | 1651 | 0.010 | 924 | 0.012 | 1114 | 0.009 | 0.010 |
|  | *TAR* | 322 | 0.002 | 196 | 0.003 | 274 | 0.002 | 0.002 |
|  | *Tork* | 414 | 0.003 | 218 | 0.003 | 429 | 0.003 | 0.003 |
| ***LINE*** |  | **1051** | **0.006** | **410** | **0.005** | **459** | **0.004** | **0.005** |
| ***Unclassified LTR*** |  | **11547** | **0.071** | **5349** | **0.069** | **9709** | **0.077** | **0.072** |
| ***Pararetrovirus*** |  | **7** | **0.000** | **2** | **0.000** | **9** | **0.000** | **0.000** |
| **Class II - DNA transposons** |  | **4012** | **0.024** | **2352** | **0.031** | **3693** | **0.029** | **0.028** |
|  | *CACTA* | 1480 | 0.009 | 964 | 0.013 | 1519 | 0.012 | 0.011 |
|  | *Helitron* | 11 | 0.000 | 30 | 0.000 | 106 | 0.001 | 0.000 |
|  | *Mariner* | 19 | 0.000 | 9 | 0.000 | 14 | 0.000 | 0.000 |
|  | *Mutator* | 1651 | 0.010 | 802 | 0.010 | 1307 | 0.010 | 0.010 |
|  | *Harbinger* | 507 | 0.003 | 341 | 0.004 | 423 | 0.003 | 0.004 |
|  | *HAT* | 344 | 0.002 | 206 | 0.003 | 324 | 0.003 | 0.002 |
| **Satellite repeats** |  | **960** | **0.006** | **652** | **0.008** | **1423** | **0.011** | **0.009** |
| **Unknown repeats** |  | **10696** | **0.065** | **5742** | **0.075** | **8785** | **0.070** | **0.070** |
| **Total** |  | **47,361** | **0.2892** | **24,083** | **0.31267** | **40,993** | **0.32698** | **0.310** |

# **Table S5. Transcript proportions (TP) of repeats in three-replicate poly-A RNA libraries prepared from leaf tissue of *Setaria viridis*.**

**The material is the same as in Table S4, differing only in the type of library prepared.**

|  |  |  |  | **Leaf poly-A** |  |  |  |  |
| --- | --- | --- | --- | --- | --- | --- | --- | --- |
|  |  | Replicate 1 |  | Replicate 2 |  | Replicate 3 |  |  |
|  | **Total Reads** | 151,136,544 |  | 164,751,814 |  | 194,996,794 |  |  |
|  | **Total Organelles + rDNA + tRNA** | 5,932,140 |  | 9,677,962 |  | 8,277,431 |  |  |
| **Repeat Class/Superfamily** | **Total minus (Organelles  + rDNA+ tRNA)** | 145,204,404 |  | 155,073,852 |  | 186,719,363 |  |  |
| **Class I – RNA retroelements** | **Repeat Lineage** | **Reads mapped** | **TP %** | **Reads mapped** | **TP %** | **Reads mapped** | **TP %** | **Mean TP %** |
| ***Ty3/gypsy*** |  | **6419** | **0.004** | **6278** | **0.004** | **9855** | **0.005** | **0.005** |
|  | *Athila* | 1205 | 0.001 | 990 | 0.001 | 1700 | 0.001 | 0.001 |
|  | *CRM* | 674 | 0.000 | 758 | 0.000 | 1214 | 0.001 | 0.001 |
|  | *Ogre* | 783 | 0.001 | 593 | 0.000 | 1087 | 0.001 | 0.001 |
|  | *Reina* | 49 | 0.000 | 64 | 0.000 | 85 | 0.000 | 0.000 |
|  | *Retand* | 1481 | 0.001 | 1704 | 0.001 | 2715 | 0.001 | 0.001 |
|  | *Tekay* | 2227 | 0.002 | 2169 | 0.001 | 3054 | 0.002 | 0.002 |
| ***Ty1/copia*** |  | **13778** | **0.009** | **12086** | **0.008** | **14214** | **0.008** | **0.008** |
|  | *Ale* | 2368 | 0.002 | 2308 | 0.001 | 2527 | 0.001 | 0.001 |
|  | *Angela* | 1259 | 0.001 | 984 | 0.001 | 1355 | 0.001 | 0.001 |
|  | *Bianca* | 2 | 0.000 | 21 | 0.000 | 37 | 0.000 | 0.000 |
|  | *Ikeros* | 1003 | 0.001 | 320 | 0.000 | 1044 | 0.001 | 0.000 |
|  | *Ivana* | 2768 | 0.002 | 2511 | 0.002 | 3424 | 0.002 | 0.002 |
|  | *SIRE* | 5433 | 0.004 | 5341 | 0.003 | 4756 | 0.003 | 0.003 |
|  | *TAR* | 576 | 0.000 | 443 | 0.000 | 839 | 0.000 | 0.000 |
|  | *Tork* | 369 | 0.000 | 158 | 0.000 | 232 | 0.000 | 0.000 |
| ***LINE*** |  | **3760** | **0.003** | **3134** | **0.002** | **3844** | **0.002** | **0.002** |
| ***Unclassified LTR*** |  | **13867** | **0.010** | **13257** | **0.009** | **21529** | **0.012** | **0.010** |
| ***Pararetrovirus*** |  | **5** | **0.000** | **0** | **0.000** | **14** | **0.000** | **0.000** |
| **Class II - DNA transposons** |  | **12994** | **0.009** | **10424** | **0.007** | **17967** | **0.010** | **0.008** |
|  | *CACTA* | 2534 | 0.002 | 2132 | 0.001 | 3919 | 0.002 | 0.002 |
|  | *Helitron* | 27 | 0.000 | 63 | 0.000 | 407 | 0.000 | 0.000 |
|  | *Mariner* | 60 | 0.000 | 53 | 0.000 | 97 | 0.000 | 0.000 |
|  | *Mutator* | 6901 | 0.005 | 4804 | 0.003 | 8574 | 0.005 | 0.004 |
|  | *Harbinger* | 2507 | 0.002 | 2809 | 0.002 | 3842 | 0.002 | 0.002 |
|  | *HAT* | 965 | 0.001 | 563 | 0.000 | 1128 | 0.001 | 0.001 |
| **Satellite repeats** |  | **15** | **0.000** | **23** | **0.000** | **26** | **0.000** | **0.000** |
| **Unknown repeats** |  | **56835** | **0.039** | **56859** | **0.037** | **74972** | **0.040** | **0.039** |
| **Total** |  | **107,673** | **0.0742** | **102,061** | **0.066** | **142,421** | **0.076** | **0.0721** |

# **Table S6. Linear regression models analysing the relationship between repeat (Ty1/copia, Ty3/gypsy and DNA transposon) transcriptome proportions (TP) in leaf poly-A (intercept) and ribo-depleted libraries and their genome proportion (GP), GC content and modal sequence similarities.**

| **a)** **Leaf** |  |  |  |  |
| --- | --- | --- | --- | --- |
| *Log TP ~ Log GP + Library type (Ty1/copia)* | |  |  |  |
|  | Estimate | Std. Error | t value | Pr(>\|t\|) |
| (Intercept) | -3.2526 | 0.1597 | -20.371 | < 2e-16 *** |
| Log GP | 0.8128 | 0.1198 | 6.783 | 4.29e-10 *** |
| Library Ribo-depleted | 0.9688 | 0.1398 | 6.932 | 2.01e-10 *** |
| Residual SE = 0.7853 on 124 df, adjusted R² = 0.4101 | | |  |  |
|  |  |  |  |  |
| **b)** **Leaf** |  |  |  |  |
| *Log TP ~ Log GP + Library type (Ty3/gypsy)* | |  |  |  |
|  | Estimate | Std. Error | t value | Pr(>\|t\|) |
| (Intercept) | -3.41208 | 0.06756 | -50.51 | <2e-16 *** |
| Log GP | 0.9739 | 0.05292 | 18.4 | <2e-16 *** |
| Library Ribo-depleted | 1.17896 | 0.06479 | 18.2 | <2e-16 *** |
| Residual SE = 0.4778 on 216 df, adjusted R² = 0.7421 | | |  |  |
|  |  |  |  |  |
| **c)** **Leaf** |  |  |  |  |
| *Log TP ~ Log GP + Library type (Class II DNA Transposons)* | | |  |  |
|  | Estimate | Std. Error | t value | Pr(>\|t\|) |
| (Intercept) | -3.02577 | 0.11417 | -26.502 | <2e-16 *** |
| Log GP | 0.79922 | 0.0769 | 10.394 | <2e-16 *** |
| Library Ribo-depleted | 0.69077 | 0.07491 | 9.221 | <2e-16 *** |
| Residual SE = 0.5344 on 193 df, adjusted R² = 0.4945 | | |  |  |
|  |  |  |  |  |
| **d)** **Leaf** |  |  |  |  |
| *Log TP ~ Similarity + GC ratio + Library type (Ty1/copia)* | | |  |  |
|  | Estimate | Std. Error | t value | Pr(>\|t\|) |
| (Intercept) | -15.5437 | 2.044506 | -7.603 | 6.43e-12 *** |
| Similarity | 0.092752 | 0.021843 | 4.246 | 4.25e-05 *** |
| GC ratio | 0.059425 | 0.009652 | 6.156 | 9.66e-09 *** |
| Library Ribo-depleted | 1.00714 | 0.132933 | 7.576 | 7.38e-12 *** |
| Residual SE = 0.7455 on 123 df, adjusted R² = 0.4684 | | |  |  |
|  |  |  |  |  |
| **e)** **Leaf** |  |  |  |  |
| *Log TP ~ Similarity + GC ratio + Library type (Ty3/gypsy)* | | |  |  |
|  | Estimate | Std. Error | t value | Pr(>\|t\|) |
| (Intercept) | -9.71802 | 1.33954 | -7.255 | 7.20e-12 *** |
| Similarity | 0.03794 | 0.01483 | 2.559 | 0.0112 * |
| GC ratio | 0.0439 | 0.00629 | 6.98 | 3.62e-11 *** |
| Library Ribo-depleted | 1.10705 | 0.09144 | 12.107 | < 2e-16 *** |
| Residual SE = 0.6755 on 215 df, adjusted R² = 0.4845 | | |  |  |
|  |  |  |  |  |
| **f)** **Leaf** |  |  |  |  |
| *Log TP ~ Similarity + GC ratio + Library type (Class II DNA transposons)* | | | |  |
|  | Estimate | Std. Error | t value | Pr(>\|t\|) |
| (Intercept) | -1.625 | 1.784305 | -0.911 | 0.364 |
| Similarity | -0.029468 | 0.018165 | -1.622 | 0.106 |
| GC ratio | 0.008457 | 0.012903 | 0.655 | 0.513 |
| Library Ribo-depleted | 0.689092 | 0.093078 | 7.403 | 4.06e-12 *** |
| Residual SE = 0.6515 on 192 df, adjusted R² = 0.2197 | | |  |  |
| Signif. codes: 0 ‘***’ 0.001 ‘**’ 0.01 ‘*’ 0.05 ‘.’ 0.1 ‘ ’ 1 | |  |  |  |

# **Table S7. Linear regression models analysing the relationship between repeat transcriptome proportions (TP) for different RNA libraries (ribo-depleted and poly-A), tissues (stem, crown and inflorescence) and genomic features (i.e. genome proportions, GP), GC content and modal sequence similarities for Ty1/copia, Ty3/gypsy and DNA transposon repeats identified using RepeatExplorer2.**

| **a) Inflorescence poly-A RNA:** *Log TP~ Log GP (Ty1/copia)* |  |  |  |  |
| --- | --- | --- | --- | --- |
|  | Estimate | Std. Error | t value | Pr(>\|t\|) |
| (Intercept) | -3.4123 | 0.2186 | -15.606 | <2e-16 *** |
| Log GP | 0.1478 | 0.2028 | 0.729 | 0.469 |
| Residual SE = 0.8135 on 49 df, adjusted R² = -0.009456 | | | |  |
|  |  |  |  |  |
| **b) Inflorescence poly-A RNA:** *Log TP~ Log GP (Ty3/gypsy)* |  |  |  |  |
|  | Estimate | Std. Error | t value | Pr(>\|t\|) |
| (Intercept) | -3.35971 | 0.09814 | -34.233 | < 2e-16 *** |
| Log GP | 0.68186 | 0.09397 | 7.256 | 1.03e-10 *** |
| Residual SE = 0.5387 on 96 df, adjusted R² = 0.3475 |  |  |  |  |
|  |  |  |  |  |
| **c) Inflorescence poly-A RNA:** *Log TP~ Log GP (DNA transposons)* |  |  |  |  |
|  | Estimate | Std. Error | t value | Pr(>\|t\|) |
| (Intercept) | -2.5673 | 0.1867 | -13.754 | < 2e-16 *** |
| Log GP | 0.845 | 0.1344 | 6.286 | 1.22e-08 *** |
| Residual SE = 0.625 on 88 df, adjusted R² = 0.302 | | |  |  |
|  |  |  |  |  |
| **d)** **Inflorescence poly-A RNA**: *Log TP~ Similarity + GC content (Ty1/copia)* |  |  |  |  |
|  | Estimate | Std. Error | t value | Pr(>\|t\|) |
| (Intercept) | -1.35152 | 3.53222 | -0.383 | 0.70369 |
| Similarity | -0.04597 | 0.03527 | -1.303 | 0.19866 |
| GC content | 0.04673 | 0.01696 | 2.754 | 0.00828 ** |
| Residual SE = 0.7501 on 48 df, adjusted R² = 0.1417 | | |  |  |
|  |  |  |  |  |
| **e)** **Inflorescence poly-A RNA**: *Log TP~ Similarity + GC content (Ty3/gypsy )* |  |  |  |  |
|  | Estimate | Std. Error | t value | Pr(>\|t\|) |
| (Intercept) | -6.20625 | 2.13271 | -2.91 | 0.00450 ** |
| Similarity | 0.01291 | 0.02418 | 0.534 | 0.59477 |
| GC content | 0.02436 | 0.88793 | 2.744 | 0.00727 ** |
| Residual SE = 0.6431 on 95 df, adjusted R² = 0.0701 | | |  |  |
|  |  |  |  |  |
| **f)** **Inflorescence poly-A RNA**: *Log TP~ Similarity + GC content  (DNA transposons)* |  |  |  |  |
|  | Estimate | Std. Error | t value | Pr(>\|t\|) |
| (Intercept) | -4.42139 | 2.88899 | -1.53 | 0.12934 |
| Similarity | -0.0163 | 0.02967 | -0.549 | 0.58402 |
| GC content | 0.05641 | 0.02082 | 2.71 | 0.00802 ** |
| Residual SE = 0.734 on 92 df, adjusted R² = 0.05529 | | |  |  |
|  |  |  |  |  |
| **g)** **Stem/Crown ribo-depleted:** *Log TP ~ Log GP + Tissue (Ty1/copia )* | |  |  |  |
|  | Estimate | Std. Error | t value | Pr(>\|t\|) |
| (Intercept) | -2.28065 | 0.15017 | -15.188 | < 2e-16 *** |
| Log GP | 0.9083 | 0.11366 | 7.992 | 8.52e-13 *** |
| Tissue Stem | 0.04487 | 0.13085 | 0.343 | 0.732 |
| Residual SE = 0.7314 on 122 df, adjusted R² = 0.3331 | | |  |  |
|  |  |  |  |  |
| **h)** **Stem/Crown ribo-depleted:** *Log TP ~ Log GP + Tissue (Ty3/gypsy )* | |  |  |  |
|  | Estimate | Std. Error | t value | Pr(>\|t\|) |
| (Intercept) | -2.61366 | 0.06617 | -39.502 | <2e-16 *** |
| Log GP | 0.92561 | 0.0505 | 18.329 | <2e-16 *** |
| Tissue Stem | 0.06299 | 0.0625 | 1.008 | 0.315 |
| Residual SE = 0.4667 on 220 df, adjusted R² = 0.6012 | | |  |  |
|  |  |  |  |  |
| **i)** **Stem/Crown ribo-depleted:** *Log TP ~ Log GP + Tissue (Class II DNA transposons )* | |  |  |  |
|  | Estimate | Std. Error | t value | Pr(>\|t\|) |
| (Intercept) | -2.50427 | 0.10983 | -22.801 | <2e-16 *** |
| Log GP | 0.85724 | 0.0739 | 11.601 | <2e-16 *** |
| Tissue Stem | 0.08104 | 0.07211 | 1.124 | 0.262 |
| Residual SE = 0.5099 on 197 df, adjusted R² = 0.4016 | | |  |  |
|  |  |  |  |  |
| **j) Stem/Crown ribo-depleted:** *Log TP ~ Similarity + GC content + Tissue (Ty1/copia)* | |  |  |  |
|  | | |  |  |
|  | Estimate | Std. Error | t value | Pr(>\|t\|) |
| (Intercept) | -16.795181 | 1.970463 | -8.523 | 5.14e-14 *** |
| Similarity | 0.121201 | 0.021111 | 5.741 | 7.12e-08 *** |
| GC content | 0.04771 | 0.009213 | 5.179 | 9.02e-07 *** |
| Tissue Stem | 0.037788 | 0.128718 | 0.294 | 0.77 |
| Residual SE = 0.7195 on 121 df, adjusted R² = 0.3545 | | |  |  |
|  |  |  |  |  |
| **k) Stem/Crown ribo-depleted:** *Log TP ~ Similarity + GC content + Tissue (Ty3/gypsy)* | |  |  |  |
|  | | |  |  |
|  | Estimate | Std. Error | t value | Pr(>\|t\|) |
| (Intercept) | -4.907623 | 1.407549 | -3.487 | 0.000591 *** |
| Similarity | 0.001531 | 0.015635 | 0.098 | 0.922099 |
| GC content | 0.028515 | 0.006634 | 4.298 | 2.59e-05 *** |
| Tissue Stem | 0.047935 | 0.095483 | 0.502 | 0.61615 |
| Residual SE = 0.7128 on 219 df, adjusted R² = 0.06951 | | | |  |
|  |  |  |  |  |
| **l) Stem/Crown ribo-depleted:** *Log TP ~ Similarity + GC content + Tissue (Class II DNA transposons)* | |  |  |  |
|  | | |  |  |
|  | Estimate | Std. Error | t value | Pr(>\|t\|) |
| (Intercept) | -0.71615 | 1.80064 | -0.398 | 0.691 |
| Similarity | -0.02618 | 0.01832 | -1.429 | 0.155 |
| GC content | -0.01077 | 0.01302 | -0.827 | 0.409 |
| Tissue Stem | 0.07151 | 0.09313 | 0.768 | 0.443 |
| Residual SE = 0.6585 on 196 df, adjusted R² = 0.001895 | | | |  |
|  | | |  |  |

# **Table S8. Transcript proportions (TP) of repeats in the three replicate inflorescence poly-A RNA libraries of *Setaria viridis*.**

|  |  |  |  | **Inflorescence** |  |  |  |  |
| --- | --- | --- | --- | --- | --- | --- | --- | --- |
|  |  | Replicate 1 |  | Replicate 2 |  | Replicate 3 |  |  |
|  | **Total Reads** | 11,376,412 |  | 11,519,902 |  | 11,963,907 |  |  |
|  | **Total Organelles+ rDNA** | 136,153 |  | 215,602 |  | 232,897 |  |  |
| **Repeat Class/Superfamily** | **Total minus (Organelles+ rDNA)** | 11,240,259 |  | 11,304,300 |  | 11,731,010 |  |  |
| **Class I - Retrotransposons** | **Repeat Lineage** | **Reads mapped** | **TP %** | **Reads mapped** | **TP %** | **Reads mapped** | **TP %** | **Mean TP %** |
| ***Ty3/gypsy*** |  | **2213** | **0.020** | **2393** | **0.021** | **2182** | **0.019** | **0.020** |
|  | *Athila* | 168 | 0.001 | 141 | 0.001 | 165 | 0.001 | 0.001 |
|  | *CRM* | 247 | 0.002 | 140 | 0.001 | 176 | 0.002 | 0.002 |
|  | *Ogre* | 141 | 0.001 | 142 | 0.001 | 133 | 0.001 | 0.001 |
|  | *Reina* | 23 | 0.000 | 42 | 0.000 | 41 | 0.000 | 0.000 |
|  | *Retand* | 1485 | 0.013 | 1788 | 0.016 | 1545 | 0.013 | 0.014 |
|  | *Tekay* | 149 | 0.001 | 140 | 0.001 | 122 | 0.001 | 0.001 |
| ***Ty1/copia*** |  | **2154** | **0.019** | **2172** | **0.019** | **2696** | **0.023** | **0.020** |
|  | *Ale* | 555 | 0.005 | 487 | 0.004 | 437 | 0.004 | 0.004 |
|  | *Angela* | 102 | 0.001 | 106 | 0.001 | 135 | 0.001 | 0.001 |
|  | *Bianca* | 0 | 0.000 | 0 | 0.000 | 0 | 0.000 | 0.000 |
|  | *Ikeros* | 20 | 0.000 | 16 | 0.000 | 13 | 0.000 | 0.000 |
|  | *Ivana* | 290 | 0.003 | 279 | 0.002 | 252 | 0.002 | 0.002 |
|  | *SIRE* | 1121 | 0.010 | 1220 | 0.011 | 1786 | 0.015 | 0.012 |
|  | *TAR* | 52 | 0.000 | 56 | 0.000 | 61 | 0.001 | 0.000 |
|  | *Tork* | 14 | 0.000 | 8 | 0.000 | 12 | 0.000 | 0.000 |
| ***LINE*** |  | **884** | **0.008** | **957** | **0.008** | **1097** | **0.009** | **0.009** |
| ***Unclassified LTR*** |  | **1727** | **0.015** | **1748** | **0.015** | **1638** | **0.014** | **0.015** |
| ***Pararetrovirus*** |  | **0** | **0.000** | **2** | **0.000** | **3** | **0.000** | **0.000** |
| **Class II - DNA transposons** |  | **4093** | **0.036** | **4634** | **0.041** | **4788** | **0.041** | **0.039** |
|  | *CACTA* | 405 | 0.004 | 438 | 0.004 | 519 | 0.004 | 0.004 |
|  | *Helitron* | 16 | 0.000 | 24 | 0.000 | 31 | 0.000 | 0.000 |
|  | *Mariner* | 4 | 0.000 | 3 | 0.000 | 2 | 0.000 | 0.000 |
|  | *Mutator* | 3150 | 0.028 | 3636 | 0.032 | 3569 | 0.030 | 0.030 |
|  | *Harbinger* | 444 | 0.004 | 436 | 0.004 | 532 | 0.005 | 0.004 |
|  | *HAT* | 74 | 0.001 | 97 | 0.001 | 135 | 0.001 | 0.001 |
| **Satellite repeats** |  | **5** | **0.000** | **8** | **0.000** | **7** | **0.000** | **0.000** |
| **Unknown repeats** |  | **7073** | **0.063** | **7862** | **0.070** | **8123** | **0.069** | **0.067** |
| **Total** |  | **18149** | **0.161** | **19776** | **0.175** | **20534** | **0.175** | **0.170** |

# **Table S9. Transcript proportions (TP) of repeats in the three replicate ribo-depleted RNA libraries prepared from crown tissue of *Setaria viridis*.**

|  |  | **Crown ribo-depleted** | | | | |  |  |
| --- | --- | --- | --- | --- | --- | --- | --- | --- |
|  |  | Rep1 |  | Rep2 |  | Rep3 |  |  |
|  | **Total Reads** | 22,280,606 |  | 14,717,318 |  | 21,292,550 |  |  |
|  | **Total Organelles+ rDNA** | 6,125,278 |  | 6,253,478 |  | 2,063,390 |  |  |
| **Repeat Class/Superfamily** | **Total minus (Organelles+ rDNA)** | 16,155,328 |  | 8,463,840 |  | 19,229,160 |  |  |
| **Class I - Retrotransposons** | **Repeat Lineage** | **Reads mapped** | **TP %** | **Reads mapped** | **TP %** | **Reads mapped** | **TP %** | **Mean TP%** |
| ***Ty3/gypsy*** |  | **3502** | **0.022** | **4077** | **0.048** | **4219** | **0.022** | **0.031** |
|  | *Athila* | 448 | 0.003 | 439 | 0.005 | 409 | 0.002 | 0.003 |
|  | *CRM* | 289 | 0.002 | 461 | 0.005 | 414 | 0.002 | 0.003 |
|  | *Ogre* | 584 | 0.004 | 670 | 0.008 | 671 | 0.003 | 0.005 |
|  | *Reina* | 94 | 0.001 | 57 | 0.001 | 83 | 0.000 | 0.001 |
|  | *Retand* | 1434 | 0.009 | 1304 | 0.015 | 1815 | 0.009 | 0.011 |
|  | *Tekay* | 653 | 0.004 | 1146 | 0.014 | 827 | 0.004 | 0.007 |
| ***Ty1/copia*** |  | **5868** | **0.036** | **4660** | **0.055** | **7234** | **0.038** | **0.043** |
|  | *Ale* | 759 | 0.005 | 696 | 0.008 | 1105 | 0.006 | 0.006 |
|  | *Angela* | 879 | 0.005 | 1258 | 0.015 | 899 | 0.005 | 0.008 |
|  | *Bianca* | 3 | 0.000 | 5 | 0.000 | 0 | 0.000 | 0.000 |
|  | *Ikeros* | 258 | 0.002 | 241 | 0.003 | 246 | 0.001 | 0.002 |
|  | *Ivana* | 969 | 0.006 | 584 | 0.007 | 1069 | 0.006 | 0.006 |
|  | *SIRE* | 2644 | 0.016 | 1598 | 0.019 | 3578 | 0.019 | 0.018 |
|  | *TAR* | 138 | 0.001 | 101 | 0.001 | 150 | 0.001 | 0.001 |
|  | *Tork* | 218 | 0.001 | 177 | 0.002 | 187 | 0.001 | 0.001 |
| ***LINE*** |  | **1420** | **0.009** | **1033** | **0.012** | **1347** | **0.007** | **0.009** |
| ***Unclassified LTR*** |  | **6317** | **0.039** | **4788** | **0.057** | **6376** | **0.033** | **0.043** |
| ***Pararetrovirus*** |  | **48** | **0.000** | **70** | **0.001** | **29** | **0.000** | **0.000** |
| **Class II - DNA transposons** |  | **3566** | **0.022** | **3281** | **0.039** | **3238** | **0.017** | **0.026** |
|  | *CACTA* | 672 | 0.004 | 714 | 0.008 | 706 | 0.004 | 0.005 |
|  | *Helitron* | 14 | 0.000 | 20 | 0.000 | 17 | 0.000 | 0.000 |
|  | *Mariner* | 9 | 0.000 | 6 | 0.000 | 7 | 0.000 | 0.000 |
|  | *Mutator* | 2188 | 0.014 | 1981 | 0.023 | 1864 | 0.010 | 0.016 |
|  | *Harbinger* | 491 | 0.003 | 347 | 0.004 | 439 | 0.002 | 0.003 |
|  | *HAT* | 192 | 0.001 | 213 | 0.003 | 205 | 0.001 | 0.002 |
| **Satellite repeats** |  | **66** | **0.000** | **181** | **0.002** | **101** | **0.001** | **0.001** |
| **Unknown repeats** |  | **8792** | **0.054** | **5332** | **0.063** | **10320** | **0.054** | **0.057** |
| **Total** |  | **29579** | **0.183** | **23422** | **0.277** | **32864** | **0.171** | **0.2102** |

# **Table S10. Transcript proportions (TP) of repeats in the three replicate ribo-depleted RNA libraries prepared from stem tissue of *Setaria viridis*.**

|  |  | **Stem ribo-depleted** | | | | | |  |
| --- | --- | --- | --- | --- | --- | --- | --- | --- |
|  |  | Rep1 |  | Rep2 |  | Rep3 |  |  |
|  | **Total Reads** | 34,113,920 |  | 20,053,518 |  | 30,755,030 |  |  |
|  | **Total (Organelles+ rDNA)** | 14,533,132 |  | 7,080,727 |  | 3,581,976 |  |  |
| **Repeat Class/Superfamily** | **Total minus (Organelles+ rDNA)** | 19,580,788 |  | 12,972,791 |  | 27,173,054 |  |  |
| **Class I - Retrotransposons** | **Repeat Lineage** | **Reads mapped** | **TP %** | **Reads mapped** | **TP %** | **Reads mapped** | **TP %** | **Mean TP%** |
| ***Ty3/gypsy*** |  | **2961** | **0.015** | **12162** | **0.094** | **6136** | **0.023** | **0.044** |
|  | *Athila* | 391 | 0.002 | 1167 | 0.009 | 566 | 0.002 | 0.004 |
|  | *CRM* | 254 | 0.001 | 1358 | 0.010 | 602 | 0.002 | 0.005 |
|  | *Ogre* | 551 | 0.003 | 2205 | 0.017 | 1057 | 0.004 | 0.008 |
|  | *Reina* | 84 | 0.000 | 99 | 0.001 | 127 | 0.000 | 0.001 |
|  | *Retand* | 1179 | 0.006 | 3515 | 0.027 | 2366 | 0.009 | 0.014 |
|  | *Tekay* | 502 | 0.003 | 3818 | 0.029 | 1418 | 0.005 | 0.012 |
| ***Ty1/copia*** |  | **6139** | **0.031** | **12373** | **0.095** | **9304** | **0.034** | **0.054** |
|  | *Ale* | 751 | 0.004 | 1495 | 0.012 | 1343 | 0.005 | 0.007 |
|  | *Angela* | 669 | 0.003 | 3439 | 0.027 | 1255 | 0.005 | 0.012 |
|  | *Bianca* | 0 | 0.000 | 30 | 0.000 | 4 | 0.000 | 0.000 |
|  | *Ikeros* | 289 | 0.001 | 441 | 0.003 | 408 | 0.002 | 0.002 |
|  | *Ivana* | 552 | 0.003 | 1190 | 0.009 | 1037 | 0.004 | 0.005 |
|  | *SIRE* | 3645 | 0.019 | 5103 | 0.039 | 4939 | 0.018 | 0.025 |
|  | *TAR* | 112 | 0.001 | 308 | 0.002 | 186 | 0.001 | 0.001 |
|  | *Tork* | 121 | 0.001 | 367 | 0.003 | 132 | 0.000 | 0.001 |
| ***LINE*** |  | **1348** | **0.007** | **1678** | **0.013** | **1792** | **0.007** | **0.009** |
| ***Unclassified LTR*** |  | **4807** | **0.025** | **10153** | **0.078** | **6463** | **0.024** | **0.042** |
| ***Pararetrovirus*** |  | **10** | **0.000** | **53** | **0.000** | **12** | **0.000** | **0.000** |
| **Class II - DNA transposons** |  | **3403** | **0.017** | **6986** | **0.054** | **4332** | **0.016** | **0.029** |
|  | *CACTA* | 652 | 0.003 | 2734 | 0.021 | 1320 | 0.005 | 0.010 |
|  | *Helitron* | 18 | 0.000 | 39 | 0.000 | 28 | 0.000 | 0.000 |
|  | *Mariner* | 3 | 0.000 | 14 | 0.000 | 3 | 0.000 | 0.000 |
|  | *Mutator* | 2070 | 0.011 | 2965 | 0.023 | 2134 | 0.008 | 0.014 |
|  | *Harbinger* | 463 | 0.002 | 737 | 0.006 | 574 | 0.002 | 0.003 |
|  | *HAT* | 197 | 0.001 | 497 | 0.004 | 273 | 0.001 | 0.002 |
| **Satellite repeats** |  | **38** | **0.000** | **1312** | **0.010** | **816** | **0.003** | **0.004** |
| **Unknown repeats** |  | **9339** | **0.048** | **11995** | **0.092** | **15163** | **0.056** | **0.065** |
| **Total** |  | **28045** | **0.1432** | **56712** | **0.4372** | **44018** | **0.162** | **0.2475** |
